# Design, Synthesis and Fungicidal Activity of *N*-(thiophen-2-yl) Nicotinamide Derivatives

**DOI:** 10.3390/molecules27248700

**Published:** 2022-12-08

**Authors:** Hongfei Wu, Xingxing Lu, Jingbo Xu, Xiaoming Zhang, Zhinian Li, Xinling Yang, Yun Ling

**Affiliations:** 1 Innovation Center of Pesticide Research, Department of Applied Chemistry, College of Science, China Agricultural University, Beijing 100193, China; 2 State Key Laboratory of the Discovery and Development of Novel Pesticide, Shenyang Sinochem Agrochemicals R&D Company Ltd., Shenyang 110021, China

**Keywords:** heterocycle, natural product, nicotinic acid, thiophene, fungicidal activity

## Abstract

Based on the modification of natural products and the active substructure splicing method, a series of new *N*-(thiophen-2-yl) nicotinamide derivatives were designed and synthesized by splicing the nitrogen-containing heterocycle natural molecule nicotinic acid and the sulfur-containing heterocycle thiophene. The structures of the target compounds were identified through ^1^H NMR, ^13^C NMR and HRMS spectra. The in vivo bioassay results of all the compounds against cucumber downy mildew (CDM, *Pseudoperonospora cubensis* (Berk.et Curt.) Rostov.) in a greenhouse indicated that compounds **4a** (EC_50_ = 4.69 mg/L) and **4f** (EC_50_ = 1.96 mg/L) exhibited excellent fungicidal activities which were higher than both diflumetorim (EC_50_ = 21.44 mg/L) and flumorph (EC_50_ = 7.55 mg/L). The bioassay results of the field trial against CDM demonstrated that the 10% EC formulation of compound **4f** displayed excellent efficacies (70% and 79% control efficacies, respectively, each at 100 mg/L and 200 mg/L) which were superior to those of the two commercial fungicides flumorph (56% control efficacy at 200 mg/L) and mancozeb (76% control efficacy at 1000 mg/L). *N*-(thiophen-2-yl) nicotinamide derivatives are significant lead compounds that can be used for further structural optimization, and compound **4f** is also a promising fungicide candidate against CDM that can be used for further development.

## 1. Introduction

The four main classes of fungal phytopathogens, including oomycetes, ascomycetes, basidiomycetes and deuteromycetes, severely threaten human health, food safety, and agriculture [1,2]. Fungicides are the main approaches to control plant diseases and play a critical role in modern agriculture by increasing both crop quality and yield. Nevertheless, with the widespread application of, especially overused, fungicides, the development of resistance is inevitable [3,4]. Therefore, there is an urgent demand to develop efficient, safe, and eco-friendly fungicides with innovative structures.

**Figure 1:**
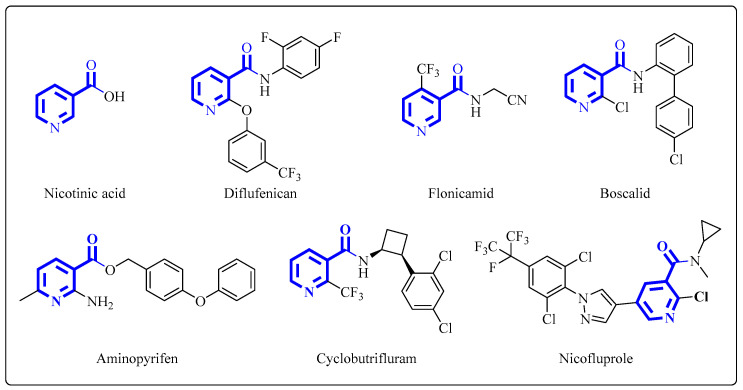
The structures of nicotinic acid and its derivatives.

Natural products bring bioinspiration to laboratories for the discovery of new weeds, plant pathogens and insect pest control agents, and they play an important role in the advancement of crop protection research [5,6,7,8,9]. Heterocycles are present in a large proportion of natural molecules and often contribute significantly to their structural and physical properties as well as to their biological activity [10,11,12,13]. Approximately 70% of all the agrochemicals that have been launched within the last 20 years bear at least one heterocyclic ring [14]. The nitrogen-containing heterocycle in the natural molecule nicotinic acid, vitamin B3, is the first lipid-lowering drug used for dyslipidemia treatment, and it has been applied for more than five decades [15]. Nicotinic acid and its derivatives play crucial roles as multifunctional pharmacophores in governing many biological activities related to physiological functions and pharmacological activities [16]. In agriculture, nicotinic acid derivatives display extensive applications as well. Agrochemicals, such as diflufenican as a herbicide, flonicamid as an insecticide and boscalid as a fungicide, have already been widely used for crop protection [17,18,19]. Currently, a few new nicotinic acid derivatives have been discovered and are under development as agrochemical candidates, for example, aminopyrifen as a fungicide candidate, cyclobutrifluram as a fungicide–nematicide candidate and nicofluprole as an insecticide candidate (Figure 1) [20,21,22]. Multidisciplinary interest has been focused on the study of nicotinic acid and its derivatives.

**Figure 2:**
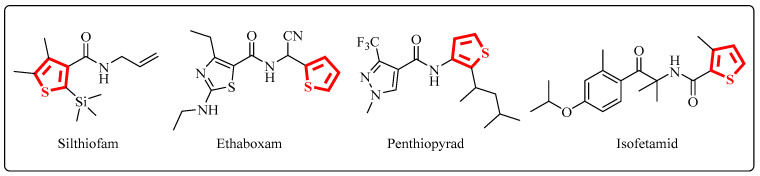
The structures of thiophene-containing agricultural fungicides.

Meanwhile, thiophene, a widely researched five-membered sulfur heterocycle, exists in commercialized agricultural fungicides, including silthiofam, ethaboxam, penthiopyrad and isofetamid (Figure 2) [23,24,25,26]. The extensive literature on thiophenes is indicative of the research on and the commercial interest in the heterocycle. Some hundreds of patents appear each year, many applying thiophene compounds as alternatives to benzenoid products, and, in many cases, the thiophene-containing molecule shows a higher activity than the benzene-containing one [27]. As an attractive small heterocycle molecule, thiophene has been widely studied for the development of novel fungicides because of its wide and satisfactory antifungal activity [28,29,30,31,32].

**Scheme 1:**
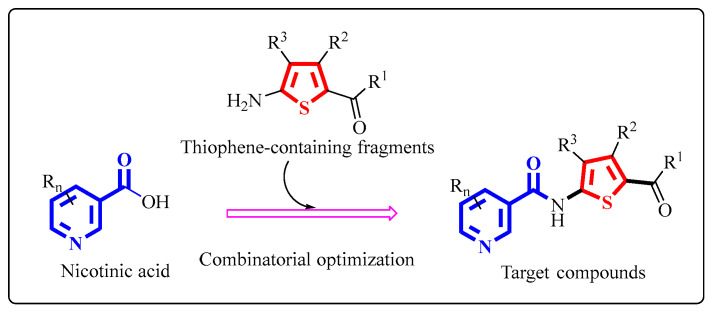
The design strategy of target compounds.

Obviously, based on the modification of natural products and the active substructure splicing method, combinations of the two active substructures of nicotinic acid and thiophene are significant for the discovery of agricultural fungicides with novel molecular structures. A series of *N*-(thiophen-2-yl) nicotinamide derivatives were designed to generate novel compounds with excellent fungicidal activity (Scheme 1).

## 2. Results and Discussion

### 2.1. Chemistry

**Scheme 2:**
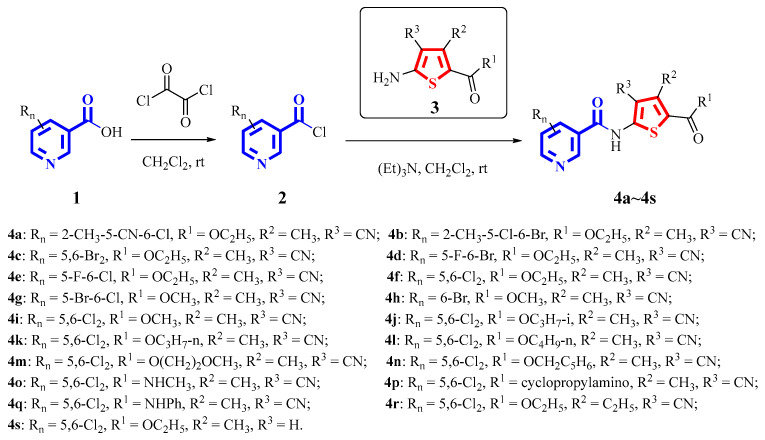
Synthetic routes of target compounds.

The synthetic pathways used to prepare target compounds **4a**~**4s** are shown in Scheme 2. The substituted nicotinic acid **1** was acyl chlorinated with oxalyl chloride to obtain acyl chloride **2**. The substituted thiophen-2-amine **3** was converted into the desired *N*-(thiophen-2-yl) nicotinamide derivatives **4a**~**4s** through acylation with the obtained acyl chloride **2** under basic conditions. Their structures were confirmed with ^1^H NMR, ^13^C NMR and HRMS spectra. Meanwhile, compound **4f** was confirmed with IR spectra (supplementary materials).

**Figure 3:**
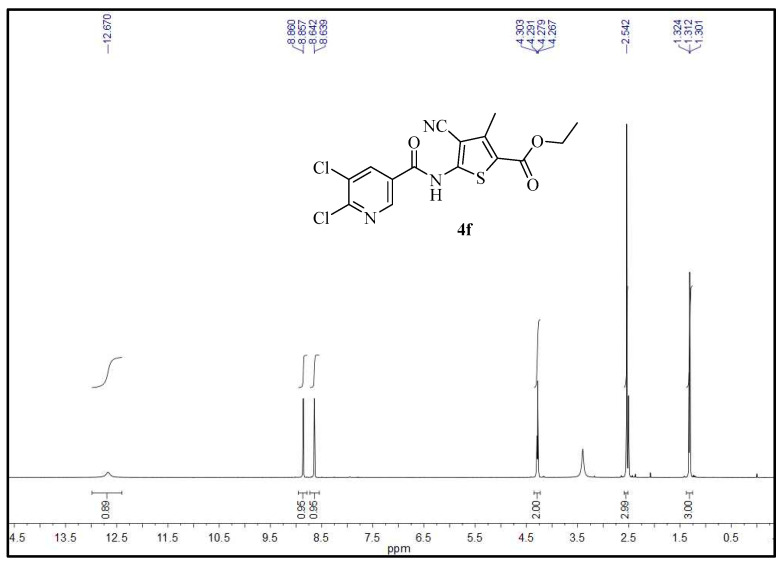
The ^1^H NMR spectrum of compound **4f**.

Compound **4f** (R_n_ = 5, 6-Cl_2_; R^1^ = OC_2_H_5_; R^2^ = CH_3_; R^3^ = CN) exhibited excellent fungicidal activities and was taken as an example to analyze the ^1^H NMR spectra data (Figure 3). The chemical shift as triplet was observed at δ 1.31 ppm with J = 7.2 Hz due to the protons of the CH_3_ of OC_2_H_5_. A singlet at δ 2.54 ppm was observed due to the protons of the CH_3_ on the thiophene ring. The chemical shift as quartet was observed at δ 4.29 ppm with J = 7.2 Hz due to the protons of the CH_2_ of OC_2_H_5_. The chemical shifts as doublet were observed at δ 8.64 ppm with J = 1.8 Hz and at δ 8.86 ppm with J = 1.8 Hz due to the protons at the fourth position and second position of pyridine, respectively. The spectrum showed a broad singlet at δ 12.67 ppm due to the proton of the CONH.

### 2.2. Fungicidal Activities In Vivo in a Greenhouse

The in vivo bioassay results shown in Table 1 indicated that, at 400 mg/L, a few compounds displayed fungicidal activities against wheat powdery mildew (WPM, *Blumeria graminis* (DC.) Speer) and southern corn rust (SCR, *Puccinia sorghi*); a few compounds had good fungicidal activities against cucumber anthracnose (CA, Colletotrichum orbiculare); and most compounds exhibited excellent fungicidal activities against cucumber downy mildew (CDM, *Pseudoperonospora cubensis* (Berk.et Curt.) Rostov.), which is one of the most destructive oomycete diseases. Further bioassay results in a greenhouse (Table 2) indicated that more than half of these compounds showed moderate to significant fungicidal activity against CDM, and there were six compounds (**4a**, **4b**, **4c**, **4f**, **4i** and **4r**) that displayed higher activities than diflumetorim (EC_50_ = 21.44 mg/L). Compounds **4a** (EC_50_ = 4.69 mg/L) and **4f** (EC_50_ = 1.96 mg/L) exhibited especially excellent activities, which were higher than flumorph (EC_50_ = 7.55 mg/L). The structure–activity relationships (SAR) were unfolded as follows.

Initially, the structural modification was mostly around the pyridine ring due to the use of the substituted nicotinic acids that were available at hand in compounds **4a**~**4h**. The testing results shown in Table 2 illustrated that compound **4f**, with a chloro at both the fifth position and sixth position of the pyridine ring, significantly indicated the highest fungicidal activity against CDM with an EC_50_ value of 1.96 mg/L. The other 5,6-dihalosubstitued pyridine compounds, **4c** (EC_50_ = 19.89 mg/L), **4d** (EC_50_ = 32.44 mg/L), **4e** (EC_50_ = 25.61 mg/L) and **4g** (EC_50_ = 34.29 mg/L), also showed good fungicidal activities, but they were much lower than those of **4f**. Additionally, **4a** (R_n_ = 2-CH_3_-5-CN-6-Cl), with a trisubstituted pyridine, had excellent fungicidal activities next to those of **4f**. It was beneficial to the increase of the fungicidal activity to have a chloro at the sixth position because the two best compounds, **4a** and **4f**, had a chloro at the sixth position.

Next, the 5,6-dichloro on the pyridine ring of compound **4f** was maintained, and the substitutions on the thiophene ring were changed to generate compounds **4i**~**4s**. Table 2 also showed that the EC_50_ values of the compounds **4i** (R^1^ = OCH_3_), **4f** (R^1^ = OC_2_H_5_), **4j** (R^1^ = OC_3_H_7_-i), **4k** (R^1^ = OC_3_H_7_-n) and **4l** (R^1^ = OC_4_H_9_-n) were 8.31 mg/L, 1.96 mg/L, 21.94 mg/L, 30.41 mg/L and 100–400 mg/L, respectively, which meant that the fungicidal activity first increased and then decreased dramatically with the increase of the carbon chain length of the alkyloxy in the R^1^ moiety and that **4f** (R^1^ = OC_2_H_5_) had the highest fungicidal activity. The fungicidal activity got much worse when R^1^ had an alkylamine instead of an alkyloxy; for example, the EC_50_ value of compound **4o** (R^1^ = NHCH_3_) was over 400 mg/L, which was much larger than that of compound **4i** (R^1^ = OCH_3_). Furthermore, when the methyl (R^2^ = CH_3_) of compound **4f** was replaced with an ethyl to generate compound **4r** (EC_50_ = 7.53 mg/L), the fungicidal activity reduced. When compound **4f** (R^3^ = CN) was decyanated to obtain compound **4s** (R^3^ = H), the fungicidal activity decreased significantly. Compound **4f** (R_n_ = 5,6-Cl_2_; R^1^ = OC_2_H_5_; R^2^ = CH_3_; R^3^ = CN) was still the most active one in all nineteen compounds.

### 2.3. Field Trials against CDM

A field trial of compound **4f** was conducted to see its fungicidal activity in the field environment (Table 3), which was a critical assessment indicator for the demonstration of whether the compound was worth further optimization or even commercial development as a fungicide candidate. The results of the field trial in 2021 against CDM demonstrated that compound **4f** displayed better control efficacies than the two commercial fungicides flumorph and mancozeb. Compound **4f** exhibited 70% and 79% control efficacies, respectively, each at a concentration of 100 mg/L and 200 mg/L, whereas flumorph at 200 mg/L and mancozeb at 1000 mg/L showed 56% and 76% control efficacies, respectively. However, the control efficacy of compound **4f** was inferior to that of the commercial fungicide cyazofamid, with a 91% control efficacy at 100 mg/L. Compared with flumorph, compound **4f**, with a much lower EC_50_ value against CDM in the greenhouse tests, was more active against CDM in the field trial.

## 3. Materials and Methods

### 3.1. Chemicals and Target Compounds

All starting materials and reagents were commercially available and used without further purification except as indicated. (The starting materials **1** and **3** were from Taizhou Jiakang Chemical Co., Ltd., a custom chemicals supplier in Zhejiang in China, and the reagents were from Sinopharm Chemical Reagent Co., Ltd., in Shanghai in China.) ^1^H nuclear magnetic resonance (NMR) spectra were obtained at 300 MHz using a Varian Mercury 300 spectrometer (Varian, Palo Alto, CA, USA), at 600 MHz using a Varian Unity Plus 600 spectrometer (Varian, Palo Alto, CA, USA) or at 600 MHz using a JEOL JNM-ECZ600R spectrometer (JEOL RESONANCE Inc., Akishima, Tokyo, Japan) with DMSO-*d_6_* as the solvent and tetramethylsilane (TMS) as the internal standard. ^13^C nuclear magnetic resonance (NMR) spectra were obtained at 150 MHz using a Varian Unity Plus 600 spectrometer (Varian, Palo Alto, CA, USA) or at 150 MHz using a JEOL JNM-ECZ600R spectrometer (JEOL RESONANCE Inc., Akishima, Tokyo, Japan) with DMSO-*d_6_* as the solvent and tetramethylsilane (TMS) as the internal standard. Chemical-shift values (*δ*) were given in parts per million (ppm). Mass spectra were acquired with a Thermo Scientific Q Exactive Focus mass spectrometer system (Thermo Fisher Scientific Inc., Waltham, MA, USA). Melting points were determined on an X-4 precision microscope melting point tester and were uncorrected. Petroleum ether used for column chromatography had a boiling range of 60~90 °C. Chemical names were generated using ChemDraw (Cambridge Soft, version 15.0). Yields were not optimized. An overview synthesis of *N*-(thiophen-2-yl)nicotinamide derivatives **4a**~**4s** is shown in Scheme 2.

#### 3.1.1. General Synthetic Procedures

General procedure for the preparation of compounds **4a**~**4s**: To a solution of acid **1** (2.3 mmol) in CH_2_Cl_2_ (20 mL), oxalyl chloride (6.9 mmol) was added dropwise, and then a drop of DMF was added. The mixture was stirred at room temperature for 6 h and concentrated under reduced pressure to obtain acyl chloride **2**. To a mixture of amine **3** (2.0 mmol) and triethylamine (2.4 mmol) in CH_2_Cl_2_ (20 mL), the solution of acyl chloride **2** in CH_2_Cl_2_ (10 mL) was added dropwise in an ice-water bath. The resulting mixture was stirred at room temperature until TLC indicated that the reaction was complete. To the mixture, water (10 mL) was added, and then the organic phase was washed with brine, dried over anhydrous MgSO_4_ and concentrated under reduced pressure to give a crude product. The crude product was purified through chromatography on a column of silica gel with petroleum ether and ethyl acetate to obtain the product amide **4**.

#### 3.1.2. Chemical Property of the Compounds

Ethyl 5-(6-chloro-5-cyano-2-methylnicotinamido)-4-cyano-3-methylthiophene-2-carboxylate (**4a**): White solid (0.58 g, yield 74%). Decomposition temperature > 175 °C. ^1^H NMR (600 MHz, DMSO-*d_6_*): *δ* 12.78 (bs, 1H, CONH), 8.77 (s, 1H, pyridine-4-H), 4.29 (q, *J* = 7.2 Hz, 2H, O*CH_2_*CH_3_), 2.63 (s, 3H, CH_3_), 2.56 (s, 3H, CH_3_) and 1.31 (t, *J* = 7.2 Hz, 3H, OCH_2_*CH_3_*). ^13^C NMR (150 MHz, DMSO*-d_6_*): *δ* 164.7, 162.3, 161.8, 151.8, 144.2, 144.1, 128.9, 118.1, 118.0, 115.1, 113.8, 106.8, 98.6, 61.4, 23.4, 14.7 and 14.5. HR-MS (*m*/*z*): calcd. for C_17_H_12_ClN_4_O_3_S [M-H]^−^ 387.0324; found, 387.0327.

Ethyl 5-(6-bromo-5-chloro-2-methylnicotinamido)-4-cyano-3-methylthiophene-2-carboxylate (**4b**): White solid (0.66 g, yield 74%). Decomposition temperature > 196 °C. ^1^H NMR (600 MHz, DMSO-*d_6_*): *δ* 12.71 (bs, 1H, CONH), 8.44 (s, 1H, pyridine-4-H), 4.24 (q, *J* = 7.2 Hz, 2H, O*CH_2_*CH_3_), 2.49 (s, 3H, CH_3_), 2.45 (s, 3H, CH_3_) and 1.27 (t, *J* = 7.2 Hz, 3H, OCH_2_*CH_3_*). ^13^C NMR (150 MHz, DMSO*-d_6_*): *δ* 165.0, 162.0, 156.6, 151.4, 150.3, 144.3, 143.2, 130.0, 118.2, 116.2, 113.9, 98.6, 61.5, 22.4, 14.8 and 14.7. HR-MS (*m*/*z*): calcd. for C_16_H_13_BrClN_3_NaO_3_S [M + Na]^+^ 465.9422; found, 465.9421.

Ethyl 4-cyano-5-(5,6-dibromonicotinamido)-3-methylthiophene-2-carboxylate (**4c**): White solid (0.55g, yield 57%). Decomposition temperature > 231 °C. ^1^H NMR (300 MHz, DMSO-*d_6_*): *δ* 12.56 (bs, 1H, CONH), 8.86 (d, *J* = 2.1 Hz, 1H, pyridine-2-H), 8.69 (d, *J* = 2.1 Hz, 1H, pyridine-4-H), 4.29 (q, *J* = 6.9 Hz, 2H, O*CH_2_*CH_3_), 2.54 (s, 3H, CH_3_) and 1.34 (t, *J* = 6.9 Hz, 3H, OCH_2_*CH_3_*). ^13^C NMR (150 MHz, DMSO*-d_6_*): *δ* 163.3, 162.0, 153.2, 151.4, 149.0, 144.3, 143.1, 129.5, 119.1, 118.5, 114.0, 99.0, 61.5, 14.9 and 14.7. HR-MS (*m*/*z*): calcd. for C_15_H_10_Br_2_N_3_O_3_S [M-H]^−^ 471.8800; found, 471.8795.

Ethyl 5-(6-bromo-5-fluoronicotinamido)-4-cyano-3-methylthiophene-2-carboxylate (**4d**): White solid (0.53 g, yield 64%). Decomposition temperature > 220 °C. ^1^H NMR (600 MHz, DMSO-*d_6_*): *δ* 12.66 (bs, 1H, CONH), 8.75 (s, 1H, pyridine-2-H), 8.42 (d, *J* = 9.0 Hz, 1H, pyridine-4-H), 4.24 (q, *J* = 7.2 Hz, 2H, O*CH_2_*CH_3_), 2.50 (s, 3H, CH_3_) and 1.27 (t, *J* = 7.2 Hz, 3H, OCH_2_*CH_3_*). ^13^C NMR (150 MHz, DMSO*-d_6_*): *δ* 163.4, 162.0, 153.9 (d, ^1^*J_CF_* = 258.6 Hz), 151.3, 145.9 (d, ^3^*J_CF_* = 6.3 Hz), 141.4 (d, ^2^*J_CF_* = 19.4 Hz), 144.4, 130.0, 120.2 (d, ^2^*J_CF_* = 20.9 Hz), 118.6, 113.9, 99.1, 61.5, 14.9 and 14.7. HR-MS (*m*/*z*): calcd. for C_15_H_10_BrFN_3_O_3_S [M-H]^−^ 409.9623; found, 409.9616.

Ethyl 5-(6-chloro-5-fluoronicotinamido)-4-cyano-3-methylthiophene-2-carboxylate (**4e**): White solid (0.52 g, yield 70%). Decomposition temperature > 275 °C. ^1^H NMR (300 MHz, DMSO-*d_6_*): *δ* 12.59 (bs, 1H, CONH), 8.80 (d, *J* = 2.1 Hz, 1H, pyridine-2-H), 8.43 (dd, *J* = 9.0, 2.1 Hz, 1H, pyridine-4-H), 4.30 (q, *J* = 7.2 Hz, 2H, O*CH_2_*CH_3_), 2.57 (s, 3H, CH_3_) and 1.34 (t, *J* = 7.2 Hz, 3H, OCH_2_*CH_3_*). ^13^C NMR (150 MHz, DMSO*-d_6_*): *δ* 163.5, 162.0, 153.9 (d, ^1^*J_CF_* = 258.6 Hz), 151.5, 145.9 (d, ^3^*J_CF_* = 6.6 Hz), 144.4, 141.3 (d, ^2^*J_CF_* = 19.7 Hz), 130.1, 126.2 (d, ^2^*J_CF_* = 21.6 Hz), 118.5, 114.0, 99.1, 61.5, 14.9 and 14.7. HR-MS (*m*/*z*): calcd. for C_15_H_10_ClFN_3_O_3_S [M-H]^−^ 366.0120; found, 366.0120.

Ethyl 4-cyano-5-(5,6-dichloronicotinamido)-3-methylthiophene-2-carboxylate (**4f**): White solid (0.58 g, yield 75%). Decomposition temperature > 262 °C. ^1^H NMR (600 MHz, DMSO-*d_6_*): *δ* 12.67 (bs, 1H, CONH), 8.86 (d, *J* = 1.8 Hz, 1H, pyridine-2-H), 8.64 (d, *J* = 1.8 Hz, 1H, pyridine-4-H), 4.29 (q, *J* = 7.2 Hz, 2H, O*CH_2_*CH_3_), 2.54 (s, 3H, CH_3_) and 1.31 (t, *J* = 7.2 Hz, 3H, OCH_2_*CH_3_*). ^13^C NMR (150 MHz, DMSO*-d_6_*): *δ* 163.2, 161.8, 151.3, 151.2, 148.3, 144.2, 139.7, 129.3, 129.2, 118.3, 113.8, 98.9, 61.3, 14.7 and 14.5. HR-MS (*m*/*z*): calcd. for C_15_H_11_Cl_2_N_3_NaO_3_S [M + Na]^+^ 405.9791; found, 405.9793.

Methyl 5-(5-bromo-6-chloronicotinamido)-4-cyano-3-methylthiophene-2-carboxylate (**4g**): White solid (0.54 g, yield 64%). Decomposition temperature > 238 °C. ^1^H NMR (600 MHz, DMSO-*d_6_*): *δ* 12.62 (bs, 1H, CONH), 8.82 (d, *J* = 1.8 Hz, 1H, pyridine-2-H), 8.68 (d, *J* = 1.8 Hz, 1H, pyridine-4-H), 3.78 (s, 3H, OCH_3_) and 2.49 (s, 3H, CH_3_). ^13^C NMR (150 MHz, DMSO*-d_6_*): *δ* 163.3, 162.4, 153.2, 151.5, 149.0, 144.5, 143.1, 129.1, 119.5, 118.2, 113.9, 99.0, 52.7 and 14.9. HR-MS (*m*/*z*): calcd. for C_14_H_10_BrClN_3_O_3_S [M + H]^+^ 415.9284; found, 415.9289.

Methyl 5-(6-bromonicotinamido)-4-cyano-3-methylthiophene-2-carboxylate (**4h**): White solid (0.55 g, yield 72%). Decomposition temperature > 261 °C. ^1^H NMR (300 MHz, DMSO-*d_6_*): *δ* 12.60 (bs, 1H, CONH), 8.91 (s, 1H, pyridine-2-H), 8.34 (d, *J* = 8.1 Hz, 1H, pyridine-4-H), 7.67 (d, *J* = 8.1 Hz, 1H, pyridine-5-H), 3.83 (s, 3H, OCH_3_) and 2.56 (s, 3H, CH_3_). ^13^C NMR (150 MHz, DMSO*-d_6_*): *δ* 164.7, 162.5, 154.2, 150.7, 149.8, 144.6, 140.5, 128.0, 124.6, 117.9, 114.0, 99.0, 52.7 and 14.9. HR-MS (*m*/*z*): calcd. for C_14_H_11_BrN_3_O_3_S [M + H]^+^ 381.9676; found, 381.9679.

Methyl 4-cyano-5-(5,6-dichloronicotinamido)-3-methylthiophene-2-carboxylate (**4i**): White solid (0.56 g, yield 75%). Decomposition temperature > 263 °C. ^1^H NMR (300 MHz, DMSO-*d_6_*): *δ* 12.55 (bs, 1H, CONH), 8.85 (d, *J* = 2.1 Hz, 1H, pyridine-2-H), 8.59 (d, *J* = 2.1 Hz, 1H, pyridine-4-H), 3.84 (s, 3H, OCH_3_) and 2.57 (s, 3H, CH_3_). ^13^C NMR (150 MHz, DMSO*-d_6_*): *δ* 163.4, 162.4, 151.6, 151.4, 148.5, 144.6, 139.9, 129.4, 129.3, 118.1, 114.0, 99.0, 52.7 and 14.9. HR-MS (*m*/*z*): calcd. for C_14_H_8_Cl_2_N_3_O_3_S [M-H]^−^ 367.9668; found, 367.9671.

Isopropyl 4-cyano-5-(5,6-dichloronicotinamido)-3-methylthiophene-2-carboxylate (**4j**): White solid (0.57 g, yield 71%). An m.p. of 239~240 °C. ^1^H NMR (300 MHz, DMSO-*d_6_*): *δ* 12.58 (bs, 1H, CONH), 8.86 (d, *J* = 2.1 Hz, 1H, pyridine-2-H), 8.61 (d, *J* = 2.1 Hz, 1H, pyridine-4-H), 5.11 (hept, *J* = 6.3 Hz, 1H, O*CH*(CH_3_)_2_), 2.56 (s, 3H, CH_3_) and 1.34 (d, *J* = 6.3 Hz, 6H, OCH*(CH_3_)_2_*). ^13^C NMR (150 MHz, DMSO*-d_6_*): *δ* 163.4, 161.6, 151.3 (2 C), 148.5, 144.2, 139.9, 129.4 (2 C), 118.8, 114.1, 99.4, 69.2, 22.2 (2 C) and 14.9. HR-MS (*m*/*z*): calcd. for C_16_H_12_Cl_2_N_3_O_3_S [M-H]^−^ 395.9981; found, 395.9986.

Propyl 4-cyano-5-(5,6-dichloronicotinamido)-3-methylthiophene-2-carboxylate (**4k**): White solid (0.56 g, yield 70%). An m.p. of 229~230 °C. ^1^H NMR (300 MHz, DMSO-*d_6_*): *δ* 12.61 (bs, 1H, CONH), 8.87 (d, *J* = 2.1 Hz, 1H, pyridine-2-H), 8.62 (d, *J* = 2.1 Hz, 1H, pyridine-4-H), 4.21 (t, *J* = 6.6 Hz, 1H, O*CH_2_*CH_2_CH_3_), 2.57 (s, 3H, CH_3_), 1.69–1.81 (m, 2H, OCH_2_*CH_2_*CH_3_) and 1.01 (t, *J* = 6.9 Hz, 3H, OCH_2_CH_2_*CH_3_*). ^13^C NMR (150 MHz, DMSO*-d_6_*): *δ* 163.5, 162.1, 151.3 (2 C), 148.5, 144.3, 139.9, 129.4 (2 C), 118.4, 114.1, 99.4, 66.8, 22.1, 14.9 and 10.9. HR-MS (*m*/*z*): calcd. for C_16_H_12_Cl_2_N_3_O_3_S [M-H]^−^ 395.9981; found, 395.9986.

Butyl 4-cyano-5-(5,6-dichloronicotinamido)-3-methylthiophene-2-carboxylate (**4l**): White solid (0.55 g, yield 66%). An m.p. of 215~216 °C. ^1^H NMR (600 MHz, DMSO-*d_6_*): *δ* 12.66 (bs, 1H, CONH), 8.83 (d, *J* = 2.1 Hz, 1H, pyridine-2-H), 8.62 (d, *J* = 2.1 Hz, 1H, pyridine-4-H), 4.21 (t, *J* = 6.6 Hz, 1H, O*CH_2_*CH_2_CH_2_CH_3_), 2.51 (s, 3H, CH_3_), 1.62–1.64 (m, 2H, OCH_2_*CH_2_*CH_2_CH_3_), 1.36–1.39 (m, 2H, OCH_2_CH_2_*CH_2_*CH_3_) and 0.89 (t, *J* = 7.2 Hz, 3H, OCH_2_CH_2_CH_2_*CH_3_*). ^13^C NMR (150 MHz, DMSO*-d_6_*): *δ* 163.5, 162.1, 151.3 (2 C), 148.5, 144.4, 139.9, 129.4 (2 C), 120.6, 114.1, 99.1, 65.1, 30.7, 19.3, 14.9 and 14.1. HR-MS (*m*/*z*): calcd. for C_17_H_14_Cl_2_N_3_O_3_S [M-H]^−^ 410.0138; found, 410.0145.

2-methoxyethyl 4-cyano-5-(5,6-dichloronicotinamido)-3-methylthiophene-2-carboxylate (**4m**): White solid (0.58 g, yield 69%). An m.p. of 213~214 °C. ^1^H NMR (300 MHz, DMSO-*d_6_*): *δ* 12.62 (bs, 1H, CONH), 8.86 (d, *J =* 2.1 Hz, 1H, pyridine-2-H), 8.62 (d, *J* = 2.1 Hz, 1H, pyridine-4-H), 4.34–4.38 (m, 2H, O*CH_2_*CH_2_OCH_3_), 3.62–3.65 (m, 2H, OCH_2_*CH_2_*OCH_3_), 3.33 (s, 3H, OCH_2_CH_2_O*CH_3_*) and 2.57 (s, 3H, CH_3_). ^13^C NMR (150 MHz, DMSO*-d_6_*): *δ* 163.4, 161.9, 151.7, 151.4, 148.5, 144.7, 139.9, 129.5, 129.3, 118.2, 114.0, 99.1, 70.2, 64.4, 58.7 and 14.9. HR-MS (*m*/*z*): calcd. for C_16_H_12_Cl_2_N_3_O_4_S [M-H]^−^ 411.9931; found, 411.9936.

Benzyl 4-cyano-5-(5,6-dichloronicotinamido)-3-methylthiophene-2-carboxylate (**4n**): White solid (0.57 g, yield 63%). Decomposition temperature > 213 °C. ^1^H NMR (300 MHz, DMSO-*d_6_*): *δ* 12.68 (bs, 1H, CONH), 8.85 (d, *J* = 2.1 Hz, 1H, pyridine-2-H), 8.63 (d, *J* = 2.1 Hz, 1H, pyridine-4-H), 7.36–7.45 (m, 5H, Ph-2,3,4,5,6-5H), 5.32 (s, 2H, OCH_2_) and 2.57 (s, 3H, CH_3_). ^13^C NMR (150 MHz, DMSO*-d_6_*): *δ* 163.5, 161.8, 151.7, 151.4, 148.5, 144.9, 139.9, 136.3, 129.5, 129.3, 129.1 (2 C), 128.8, 128.5 (2 C), 118.0, 113.9, 99.1, 66.8 and 14.9. HR-MS (*m*/*z*): calcd. for C_20_H_12_Cl_2_N_3_O_3_S [M-H]^−^ 443.9987; found, 443.9981.

5,6-dichloro-*N*-(3-cyano-4-methyl-5-(methylcarbamoyl)thiophen-2-yl)nicotinamide (**4o**): White solid (0.51 g, yield 68%). Decomposition temperature > 265 °C. ^1^H NMR (300 MHz, DMSO-*d_6_*): *δ* 12.44 (bs, 1H, CONH), 8.86 (d, *J* = 2.1 Hz, 1H, pyridine-2-H), 8.64 (d, *J* = 2.1 Hz, 1H, pyridine-4-H), 8.05 (q, *J* = 4.5 Hz, 3H, CO*NH*CH_3_), 2.75 (d, *J* = 4.5 Hz, 3H, CONH*CH_3_*) and 2.47 (s, 3H, CH_3_). ^13^C NMR (150 MHz, DMSO*-d_6_*): *δ* 163.0, 162.4, 151.3, 148.7, 148.4, 139.8, 137.5, 129.5, 129.4, 124.8, 114.3, 98.7, 26.9 and 14.7. HR-MS (*m*/*z*): calcd. for C_14_H_9_Cl_2_N_4_O_2_S [M-H]^−^ 366.9828; found, 366.9830.

5,6-dichloro-*N*-(3-cyano-5-(cyclopropylcarbamoyl)-4-methylthiophen-2-yl)nicotinamide (**4p**): White solid (0.55 g, yield 69%). Decomposition temperature > 258 °C; ^1^H NMR (300 MHz, DMSO-*d_6_*): *δ* 12.41 (bs, 1H, CONH), 8.86 (d, *J* = 2.1 Hz, 1H, pyridine-2-H), 8.63 (d, *J* = 2.1 Hz, 1H, pyridine-4-H), 8.19 (d, *J* = 6.9 Hz, 1H, CO*NH*CH), 2.74–2.83 (m, 1H, cyclopropane-H), 2.44 (s, 3H, CH_3_) and 0.55–0.72 (m, 4H, cyclopropane-H). ^13^C NMR (150 MHz, DMSO*-d_6_*): *δ* 163.3, 163.0, 151.3, 148.6, 148.4, 139.8, 137.7, 129.5 (2 C), 124.6, 114.3, 98.6, 23.6, 14.7 and 6.4. HR-MS (*m*/*z*): calcd. for C_16_H_13_Cl_2_N_4_O_2_S [M + H]^+^ 395.0131; found, 395.0123.

5,6-dichloro-*N*-(3-cyano-4-methyl-5-(phenylcarbamoyl)thiophen-2-yl)nicotinamide (**4q**): White solid (0.57 g, yield 65%). Decomposition temperature > 243 °C. ^1^H NMR (600 MHz, DMSO-*d_6_*): *δ* 12.56 (bs, 1H, CONH), 10.11 (s, 1H, CONH), 8.86 (d, *J* = 2.1 Hz, 1H, pyridine-2-H), 8.64 (d, *J* = 2.1 Hz, 1H, pyridine-4-H), 7.62–7.64 (m, 2H, Ph-H), 7.29–7.32 (m, 2H, Ph-H), 7.06–7.09 (m, 1H, Ph-H) and 2.47 (s, 3H, CH_3_). ^13^C NMR (150 MHz, DMSO*-d_6_*): *δ* 163.1, 160.8, 151.3, 148.5, 139.9, 139.2, 138.7, 129.5, 129.2 (2 C), 129.1, 124.8, 124.5, 121.0 (2 C), 120.8, 114.3, 98.6 and 14.9. HR-MS (*m*/*z*): calcd. for C_19_H_11_Cl_2_N_4_O_2_S [M-H]^−^ 428.9985; found, 428.9988.

Ethyl 4-cyano-5-(5,6-dichloronicotinamido)-3-ethylthiophene-2-carboxylate (**4r**): White solid (0.55 g, yield 68%). Decomposition temperature > 205 °C. ^1^H NMR (600 MHz, DMSO-*d_6_*): *δ* 12.63 (bs, 1H, CONH), 8.80 (d, *J* = 2.1 Hz, 1H, pyridine-2-H), 8.58 (d, *J* = 2.1 Hz, 1H, pyridine-4-H), 4.24 (q, *J* = 7.2 Hz, 2H, O*CH_2_*CH_3_), 2.95 (q, *J* = 7.2 Hz, 2H, *CH_2_*CH_3_), 1.26 (t, *J* = 7.2 Hz, 3H, OCH_2_*CH_3_*) and 1.12 (t, *J* = 7.2 Hz, 3H, CH_2_*CH_3_*). ^13^C NMR (150 MHz, DMSO*-d_6_*): *δ* 163.3, 161.6, 151.7, 151.4, 150.5, 148.5, 139.8, 129.5, 129.2, 118.1, 113.8, 98.1, 61.5, 22.0, 14.8 and 14.6. HR-MS (*m*/*z*): calcd. for C_16_H_14_Cl_2_N_3_O_3_S [M + H]^+^ 398.0128; found, 398.0120.

Ethyl 5-(5,6-dichloronicotinamido)-3-methylthiophene-2-carboxylate (**4s**): White solid (0.53 g, yield 73%). Decomposition temperature > 211 °C. ^1^H NMR (600 MHz, DMSO-*d_6_*): *δ* 12.04 (bs, 1H, CONH), 8.85 (d, *J* = 2.4 Hz, 1H, pyridine-2-H), 8.56 (d, *J* = 2.4 Hz, 1H, pyridine-4-H), 6.73 (s, 1H, thiophene-H), 4.18 (q, *J* = 7.2 Hz, 2H, O*CH_2_*CH_3_), 2.40 (s, 3H, CH_3_) and 1.25 (t, *J* = 7.2 Hz, 3H, OCH_2_*CH_3_*). ^13^C NMR (150 MHz, DMSO*-d_6_*): *δ* 163.1, 160.9, 151.2, 147.9, 144.5, 143.9, 138.9, 129.9, 129.5, 117.7, 117.5, 60.6, 16.3 and 14.8. HR-MS (*m*/*z*): calcd. for C_14_H_13_Cl_2_N_2_O_3_S [M + H]^+^ 359.0019; found, 359.0012.

### 3.2. Fungicidal Activities In Vivo in a Greenhouse

Each of the test compounds (4 mg) were first dissolved in 5 mL of dimethyl sulfoxide, and then 5 mL of water containing 0.1% Tween 80 was added to generate 10 mL stock solutions at a concentration of 400 mg/L. Serial test solutions were prepared by diluting the above solution (testing range of 1.5625~400 mg/L). Evaluations of the in vivo fungicidal activity of the synthesized compounds against *Blumeria graminis* (DC.) Speer, *Puccinia sorghi*, *Colletotrichum orbiculare* and *Pseudoperonospora cubensis* (Berk.et Curt.) Rostov. Were performed as follows: Seeds (wheat: *Triticum aestivum* L., maize: *Zea mays* L., and cucumber: *Cucumis sativus* L.) were grown to the one-leaf, two-leaf, and two- to three-leaf stages, and then the test solutions were sprayed on the host plants with a homemade sprayer. After 24 h, the leaves of the host plants were inoculated with sporangial suspensions of the fungi which were cultured by Shenyang Sinochem Agrochemicals R&D Company Ltd. (Shenyang, China), each at a concentration of 5 × 10^5^ spores/mL, using a PS289 Procon Boy WA double action 0.3 mm airbrush (GSI, Tokyo, Japan), but the fungi *Blumeria graminis* (DC.) Speer was inoculated by shaking off the spores directly onto the wheat leaves. The plants were stored in a humidity chamber (24 ± 1 °C, RH > 90%, dark) and then were transferred to a greenhouse (18~30 °C, RH > 50~60%) 24 h after infection. Three replicates were carried out. The activity of each compound was estimated through visual inspection after 7 d, and the screening results were reported in the range of 0% (no control of the fungus) to 100% (complete control of the fungus). The inhibitory activity (%) was estimated as:

Inhibitory activity (%) = [(viability of the blank control − viability of the treated plant)/viability of the blank control] × 100%

The EC_50_ values were calculated with Duncan’s new multiple-range test (DMRT) using DPS version 14.5.

### 3.3. Field Trials against CDM

Field trials were conducted in the open field owned by Shenyang Sinochem Agrochemicals R&D Company Ltd. in Shenyang, Liaoning province (25 m^2^, at the five-leaf stage). The test solution was sprayed on the host plant (*Cucumis sativus* L.) with a WS-15D knapsack electric sprayer (WishSprayer, Shandong, China). Two spays were carried out with an interval period of 6~7 d. Seven days after the second treatment, the incidence of disease spots in each plot was investigated by randomly selecting 4 samples per plot and 8 plants per sample. The incidence of the whole plant was recorded by counting the number of diseased leaves and by determining the incidence grade [33]. The grade scales were divided into six levels (ratio of leaf-spot area to leaf area): level 0 (no disease), level 1 (<5%), level 3 (6~10%), level 5 (11~25%), level 7 (26~50%), and level 9 (> 51%). The disease index (DI, %) was calculated as

DI (%) = [∑ (number of diseased leaves × relative level)/(total number of investigated leaves × the highest level)] × 100%

The inhibitory activity (%) was calculated as

Inhibitory activity (%) = [(DI of the blank control − DI of the treated plot)/DI of the blank control] × 100%

## 4. Conclusions

In summary, nineteen *N*-(thiophen-2-yl) nicotinamide derivatives were designed and synthesized. The in vivo bioassay results of all nineteen compounds against WPM, SCR, CA and CDM in a greenhouse indicated that over half of these compounds showed moderate to significant fungicidal activity against CDM, and there were six compounds (**4a**, **4b**, **4c**, **4f**, **4i** and **4r**) that displayed higher activities than diflumetorim (EC_50_ = 21.44 mg/L); compounds **4a** (EC_50_ = 4.69 mg/L) and **4f** (EC_50_ = 1.96 mg/L) exhibited especially excellent activities, which were higher than those of flumorph (EC_50_ = 7.55 mg/L). The bioassay results of the field trial against CDM demonstrated that compound **4f** displayed excellent control efficacies (70% and 79% control efficacies, respectively, each at 100 mg/L and 200 mg/L), which were superior to those of the two commercial fungicides flumorph (56% control efficacy at 200 mg/L) and mancozeb (76% control efficacy at 1000 mg/L). However, the control efficacy of compound **4f** was inferior to that of the commercial fungicide cyazofamid (91% control efficacy at 100 mg/L). This study illustrated that *N*-(thiophen-2-yl) nicotinamide derivatives are significant lead compounds that can be used for the further discovery of new compounds to control the oomycete disease CDM, and it illustrated that compound **4f** is also a promising fungicide candidate against CDM that can be used for further development.

## Figures and Tables

**Table 1:** The in vivo fungicidal activities of target compounds **4a**~**4s** in green house at 400 mg/L.

Compd.	R_n_	R^1^	R^2^	R^3^	WPM	SCR	CA	CDM
**4a**	2-CH_3_-5-CN-6-Cl	OC_2_H_5_	CH_3_	CN	0	0	0	100
**4b**	2-CH_3_-5-Cl-6-Br	OC_2_H_5_	CH_3_	CN	0	50	0	100
**4c**	5,6-Br_2_	OC_2_H_5_	CH_3_	CN	0	0	100	100
**4d**	5-F-6-Br	OC_2_H_5_	CH_3_	CN	0	0	0	100
**4e**	5-F-6-Cl	OC_2_H_5_	CH_3_	CN	0	0	0	100
**4f**	5,6-Cl_2_	OC_2_H_5_	CH_3_	CN	0	0	0	100
**4g**	5-Br-6-Cl	OCH_3_	CH_3_	CN	0	0	100	100
**4h**	6-Br	OCH_3_	CH_3_	CN	0	0	100	100
**4i**	5,6-Cl_2_	OCH_3_	CH_3_	CN	0	0	0	98
**4j**	5,6-Cl_2_	OC_3_H_7_-i	CH_3_	CN	0	0	40	100
**4k**	5,6-Cl_2_	OC_3_H_7_-n	CH_3_	CN	0	0	30	100
**4l**	5,6-Cl_2_	OC_4_H_9_-n	CH_3_	CN	0	0	0	100
**4m**	5,6-Cl_2_	OC_2_H_4_OCH_3_	CH_3_	CN	0	0	0	50
**4n**	5,6-Cl_2_	OCH_2_C_6_H_5_	CH_3_	CN	0	0	0	85
**4o**	5,6-Cl_2_	NHCH_3_	CH_3_	CN	0	0	30	0
**4p**	5,6-Cl_2_	Cyclopropylamino	CH_3_	CN	0	0	0	0
**4q**	5,6-Cl_2_	NHPh	CH_3_	CN	50	100	85	98
**4r**	5,6-Cl_2_	OC_2_H_5_	C_2_H_5_	CN	0	0	0	100
**4s**	5,6-Cl_2_	OC_2_H_5_	CH_3_	H	0	0	0	100
Azoxystrobin					100	100	100	/ ^a^
Diflumetorim					/ ^a^	/ ^a^	/ ^a^	100
Flumorph					/ ^a^	/ ^a^	/ ^a^	100

^a^: Not tested.

**Table 2:** EC_50_ values of target compounds **4a**~**4s** against cucumber downy mildew in green house.

Compd.	Y = ax + b	EC_50_ (mg/L)	95% CI ^a^	r
**4a**	y = 4.14x + 1.28	4.69	3.27–6.71	0.98
**4b**	y = 1.71x + 2.92	16.52	13.27–20.58	0.94
**4c**	y = 1.87x + 2.57	19.89	16.10–24.56	0.95
**4d**	y = 1.62x + 2.55	32.44	26.12–40.29	0.95
**4e**	y = 1.58x + 2.78	25.61	20.83–31.48	0.97
**4f**	y = 1.15x + 4.66	1.96	1.17–3.29	0.95
**4g**	y = 1.62x + 2.52	34.29	27.46–42.82	0.94
**4h**	y = 1.62x + 2.72	25.54	20.80–31.35	0.97
**4i**	y = 1.48x + 3.64	8.31	6.37–10.84	0.97
**4j**	y = 1.58x + 2.88	21.94	17.58–27.36	0.93
**4k**	y = 1.58x + 2.66	30.41	24.51–37.73	0.95
**4l**	/ ^b^	100–400	/ ^b^	/ ^b^
**4m**	/ ^b^	>400	/ ^b^	/ ^b^
**4n**	/ ^b^	100–400	/ ^b^	/ ^b^
**4o**	/ ^b^	> 400	/ ^b^	/ ^b^
**4p**	/ ^b^	>400	/ ^b^	/ ^b^
**4q**	/ ^b^	100–400	/ ^b^	/ ^b^
**4r**	y = 1.48x + 3.70	7.53	5.71–9.94	0.98
**4s**	y = 1.26x + 2.72	63.81	47.23–86.22	0.92
Diflumetorim	y = 1.43x + 3.10	21.44	17.21–26.70	0.96
Flumorph	y = 1.25x + 3.91	7.55	5.57–10.22	0.98

^a^: Confidence interval. ^b^: The value could not be measured accurately.

**Table 3:** The results of compound **4f** against CDM in field (2021, Shenyang, open field).

Compd.	Concentration (mg/L)	Control Efficacy (%)			
		r1	r2	r3	Mean
10% 4f EC	50	55	53	50	53
	100	74	66	69	70
	200	82	75	79	79
10% Flumorph EC	200	64	52	51	56
80% Mancozeb WP	1000	79	76	72	76
10% Cyazofamid SC	100	91	91	90	91
CK	(disease index)	69	61	60	63

## Data Availability

All data presented in this study are available in the article and in the Supplementary Material.

## Supplementary Materials

The following supporting information can be downloaded at https://www.mdpi.com/article/10.3390/molecules27248700/s1, ^1^H NMR, ^13^C NMR, HRMS and IR of the compound.

## Sample Availability

Samples of compounds **4a**~**4s** are available from the authors.

## References

[1] Rajasekaran K., Stromberg K.D., Cary J.W., Cleveland T.E. (2001). Broad-spectrum antimicrobial activity in vitro of the synthetic peptide D4E1. J. Agric. Food Chem..

[2] Zhang S., Liu S., Zhang J., Reiter R.J., Wang Y., Qiu D., Luo X., Khalid A.R., Wang H., Feng L. (2018). Synergistic anti-oomycete effect of melatonin with a biofungicide against oomycetic black shank disease. J. Pineal Res..

[3] Gould F., Brown Z.S., Kuzma J. (2018). Wicked evolution: Can we address the sociobiological dilemma of pesticide resistance?. Science.

[4] de Chaves M.A., Reginatto P., da Costa B.S., de Paschoal R.I., Teixeira M.L., Fuentefria A.M. (2022). Fungicide Resistance in Fusarium Graminearum Species Complex. Curr. Microbiol..

[5] Wang T., Yang S., Li H., Lu A., Wang Z., Yao Y., Wang Q. (2020). Discovery, structural optimization, and mode of action of essramycin alkaloid and its derivatives as anti-tobacco mosaic virus and anti-phytopathogenic fungus agents. J. Agric. Food Chem..

[6] Xia Q., Dong J., Li L., Wang Q., Liu Y., Wang Q. (2018). Discovery of glycosylated genipin derivatives as novel antiviral, insecticidal, and fungicidal agents. J. Agric. Food Chem..

[7] Lei P., Xu Y., Du J., Yang X.L., Yuan H.Z., Xu G.F., Ling Y. (2016). Design, synthesis and fungicidal activity of N-Substituted Benzoyl-1,2,3,4-Tetrahydroquinolyl-1-Carboxamide. Bioorg. Med. Chem. Lett..

[8] Loiseleur O. (2017). Natural products in the discovery of agrochemicals. Chimia.

[9] Sparks T.C., Hahn D.R., Garizi N.V. (2017). Natural products, their derivatives, mimics and synthetic equivalents: Role in agrochemical discovery. Pest Manag. Sci..

[10] Davison E.K., Sperry J. (2017). Natural products with heteroatom-rich ring systems. J. Nat. Prod..

[11] Taylor R.D., MacCoss M., Lawson A.D.G. (2014). Rings in drugs. J. Med. Chem..

[12] Pozharskii A.F., Soldatenkov A.T., Katritzky A.R. (2011). Heterocycles in Agriculture. Heterocycles in Life and Society.

[13] Hemmerling F., Hahn F. (2016). Biosynthesis of oxygen and nitrogen-containing heterocycles in polyketides. Beilstein J. Org. Chem..

[14] Lamberth C. (2013). Heterocyclic chemistry in crop protection. Pest Manag. Sci..

[15] Kang I., Kim S.W., Youn J.H. (2011). Effects of nicotinic acid on gene expression: Potential mechanisms and implications for wanted and unwanted effects of the lipid-lowering drug. J. Clin. Endocrinol. Metab..

[16] Sinthupoom N., Prachayasittikul V., Prachayasittikul S., Ruchirawat S., Prachayasittikul V. (2015). Nicotinic acid and derivatives as multifunctional pharmacophores for medical applications. Eur. Food Res. Technol..

[17] Ashton I.P., Abulnaja K.O., Pallett K.E., Cole D.J., Harwood J.L. (1992). Diflufenican, a carotenogenesis inhibitor, also reduces acyl lipid synthesis. Pestic. Biochem. Physiol..

[18] Morita M., Ueda T., Yoneda T., Koyanagi T., Haga T. (2007). Flonicamid, a novel insecticide with a rapid inhibitory effect on aphid feeding. Pest Manag. Sci..

[19] Ma S., Ji R., Wang X., Yu C., Yu Y., Yang X. (2019). Fluorescence detection of boscalid pesticide residues in grape juice. Optik.

[20] Hatamoto M., Aizawa R., Kobayashi Y., Fujimura M. (2019). A novel fungicide Aminopyrifen Inhibits GWT-1 protein in Glycosylphosphatidylinositol-Anchor biosynthesis in neurospora crassa. Pestic. Biochem. Physiol..

[21] O’Sullivan A.C., Loiseleur O., Staiger R., Luksch T., Pitterna T. (2013). Preparation of N-Cyclylamides as Nematicides. Patent WO.

[22] Hallenbach W., Schwarz H.G., Ilg K., Goergens U., Koebberling J., Turberg A., Boehnke N., Maue M., Velten R., Harschneck T. (2015). Preparation of Substituted Benzamides for Treating Arthropodes. Patent WO.

[23] Phillips G., Fevig T.L., Lau P.H., Klemm G.H., Mao M.K., Ma C., Gloeckner J.A., Clark A.S. (2002). Process research on the synthesis of silthiofam: A novel fungicide for wheat. Org. Process. Res. Dev..

[24] Kim D.S., Chun S.J., Jeon J.J., Lee S.W., Joe G.H. (2004). Synthesis and fungicidal activity of ethaboxam against oomycetes. Pest Manag. Sci..

[25] Yanase Y., Katsuta H., Tomiya K., Enomoto M., Sakamoto O. (2013). Development of a novel fungicide, penthiopyrad. J. Pestic. Sci..

[26] Zuniga A.I., Oliveira M.S., Rebello C.S., Peres N.A. (2020). Baseline sensitivity of botrytis cinerea isolates from strawberry to isofetamid compared to other SDHIs. Plant Dis..

[27] Swanston J. (2006). Thiophene. Ullmann’s Encyclopedia of Industrial Chemistry.

[28] Wang B., Shi Y., Zhan Y., Zhang L., Zhang Y., Wang L., Zhang X., Li Y., Li Z., Li B. (2015). Synthesis and biological activity of novel furan/thiophene and piperazine-containing (Bis)1,2,4-Triazole mannich bases. Chin. J. Chem..

[29] Zhang B., Li Y.H., Liu Y., Chen Y.R., Pan E.S., You W.W., Zhao P.L. (2015). Design, synthesis and biological evaluation of novel 1,2,4-Triazolo [3,4-b][1,3,4] Thiadiazines bearing furan and Thiophene nucleus. Eur. J. Med. Chem..

[30] Wang X., Ren Z., Mei Y., Liu M., Chen M., Si W., Yang C., Song Y. (2019). Design, synthesis, and antifungal activity of 3-(Thiophen-2-Yl)-1,5-Dihydro-2H-Pyrrol-2-One derivatives bearing a carbonic ester group. J. Heterocycl. Chem..

[31] Wu H.B., Kuang M.S., Lan H.P., Wen Y.X., Liu T.T. (2020). Novel bithiophene dimers from Echinops Latifolius as potential antifungal and nematicidal agents. J. Agric. Food Chem..

[32] Yang Z., Sun Y., Liu Q., Li A., Wang W., Gu W. (2021). Design, synthesis, and antifungal activity of novel Thiophene/Furan-1,3,4-Oxadiazole carboxamides as potent succinate dehydrogenase inhibitors. J. Agric. Food Chem..

[33] Guan A., Wang M., Yang J., Wang L., Xie Y., Lan J., Liu C. (2017). Discovery of a new fungicide candidate through lead optimization of Pyrimidinamine derivatives and its activity against cucumber downy mildew. J. Agric. Food Chem..

