# Supplementary material for: Design, Synthesis and Fungicidal Activity of N-(thiophen-2-yl) Nicotinamide Derivatives
Source: Molecules. 2022 Dec 8;27(24):8700. doi: 10.3390/molecules27248700 (PMC9783666; doi:10.3390/molecules27248700)

Supporting information

Design, Synthesis and Fungicidal Activity of *N*-(thiophen-2-yl) Nicotinamide Derivatives

Hongfei Wu, Xingxing Lu, Jingbo Xu, Xiaoming Zhang, Zhinian Li, Xinling Yang, *, Yun Ling *

^{1}H NMR SPECTRAL GRAPH

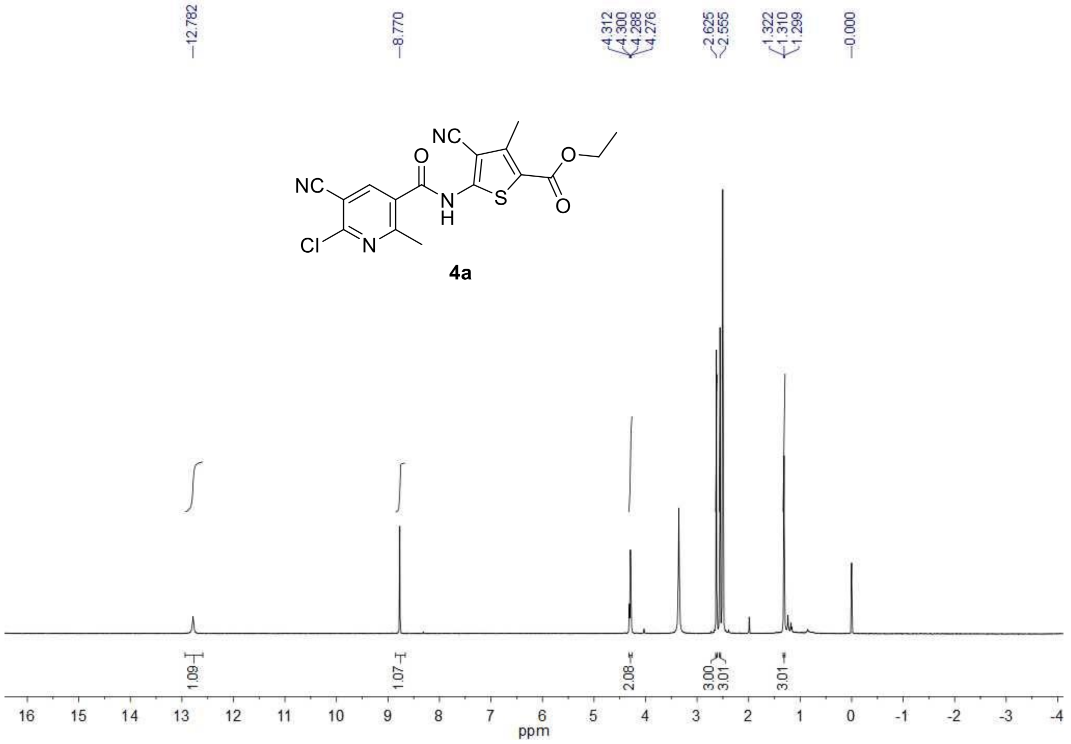

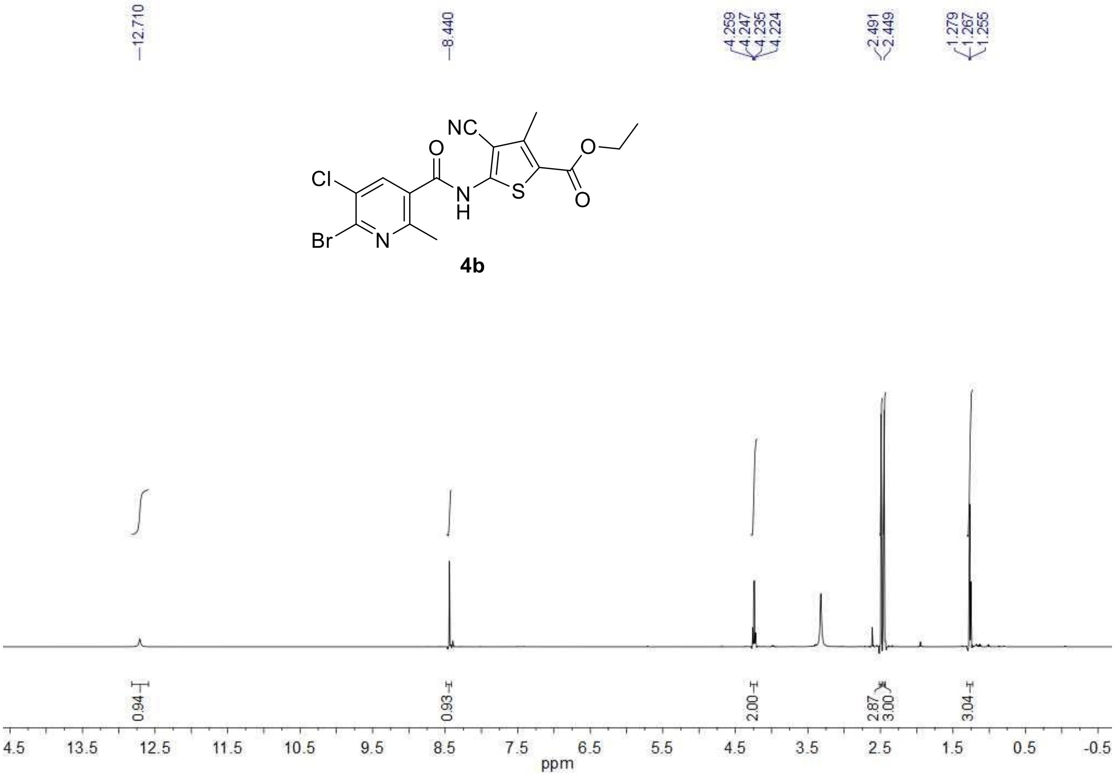

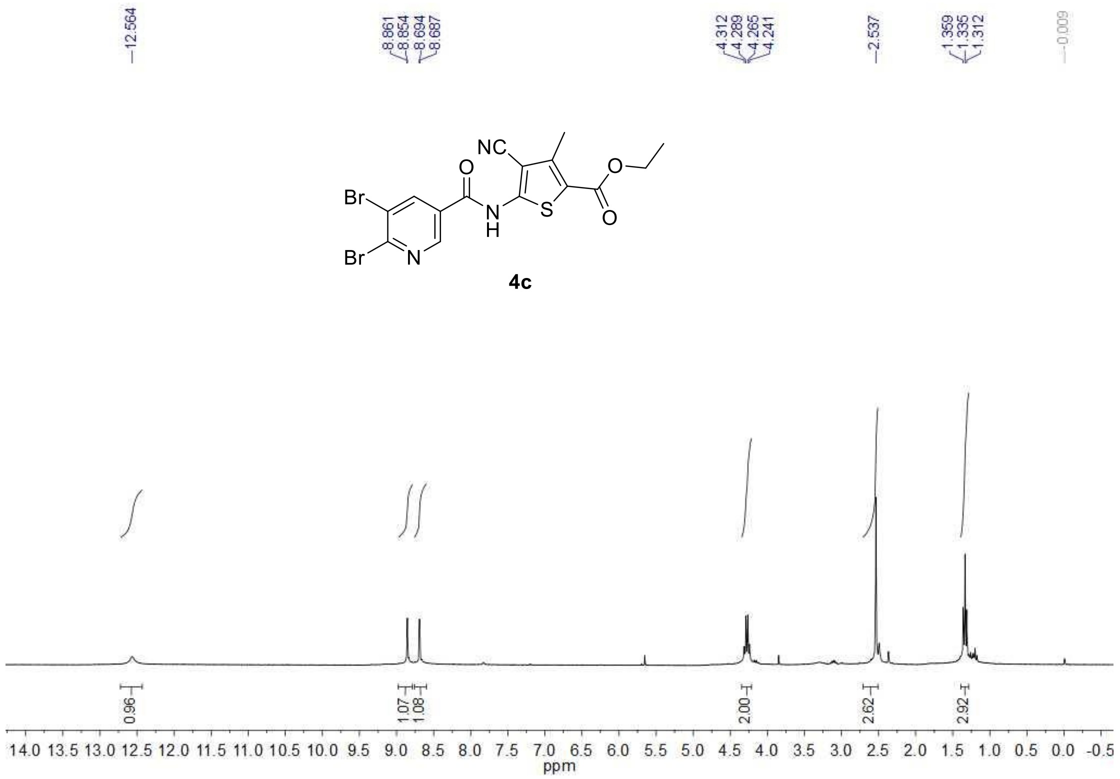

12.564
8.861
8.854
8.694
8.687
4.312
4.289
4.265
4.241
2.537
1.359
1.335
1.312
0.009
NC
O
Br
N
H
S
O
O
Br
N
0.96
1.07
1.08
2.00
2.62
2.92
14.0 13.5 13.0 12.5 12.0 11.5 11.0 10.5 10.0 9.5 9.0 8.5 8.0 7.5 7.0 6.5 6.0 5.5 5.0 4.5 4.0 3.5 3.0 2.5 2.0 1.5 1.0 0.5 0.0 -0.5
ppm

4c

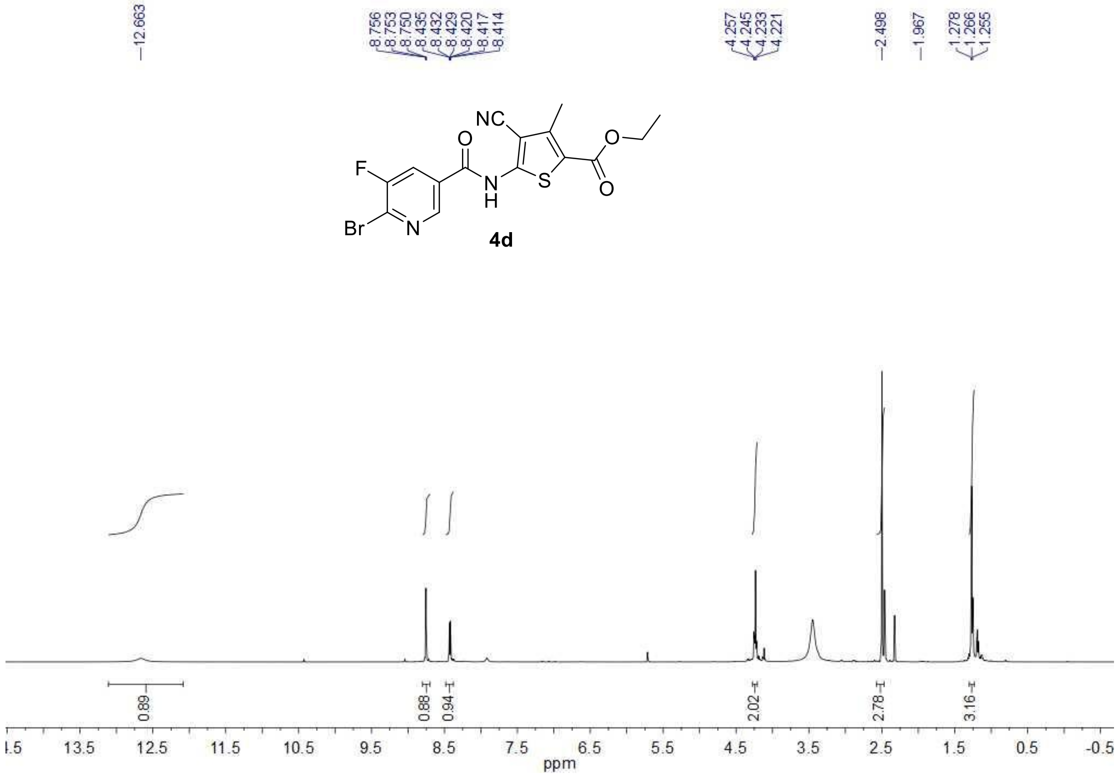

12.663
8.756
8.753
8.750
8.435
8.432
8.429
8.420
8.417
8.414
4.257
4.245
4.233
4.221
2.498
1.967
1.278
1.266
1.255
NC
O
F
N
H
S
O
O
Br
N
0.89
0.88
0.94
2.02
2.78
3.16
13.5 12.5 11.5 10.5 9.5 8.5 7.5 6.5 5.5 4.5 3.5 2.5 1.5 0.5 -0.5
ppm

4d

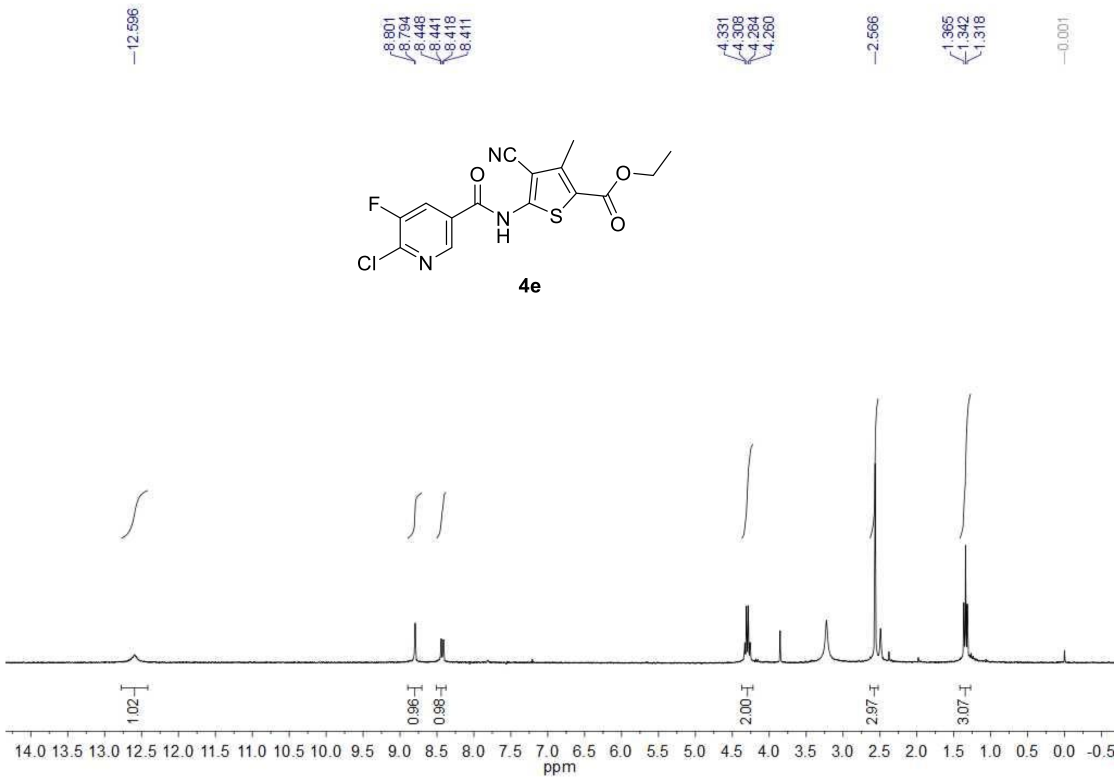
12.596
8.801
8.794
8.448
8.441
8.418
8.411
4.331
4.308
4.284
4.260
2.566
1.365
1.342
1.318
0.001
NC
F
O
N
H
S
O
O
Cl
N
1.02
0.96
0.98
2.00
2.97
3.07
14.0 13.5 13.0 12.5 12.0 11.5 11.0 10.5 10.0 9.5 9.0 8.5 8.0 7.5 7.0 6.5 6.0 5.5 5.0 4.5 4.0 3.5 3.0 2.5 2.0 1.5 1.0 0.5 0.0 -0.5
ppm

4e

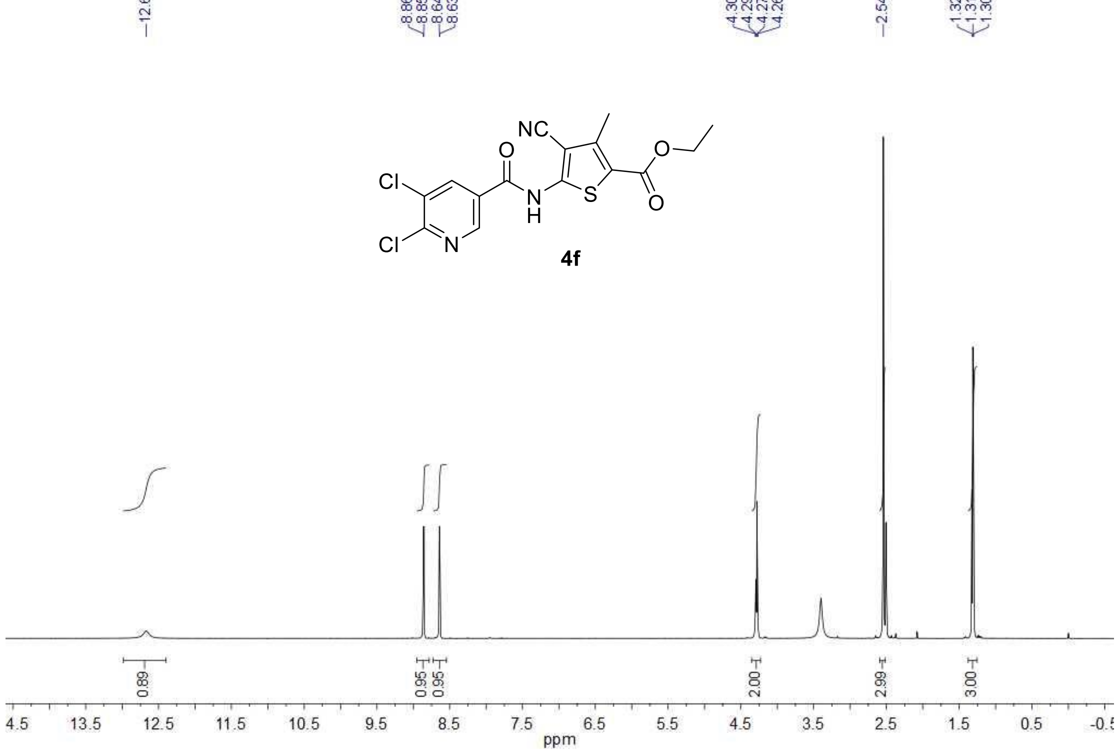
12.670
8.860
8.857
8.642
8.639
4.303
4.291
4.279
4.267
2.542
1.324
1.312
1.301
NC
Cl
O
N
H
S
O
O
Cl
N
0.89
0.95
0.95
2.00
2.99
3.00
4.5 13.5 12.5 11.5 10.5 9.5 8.5 7.5 6.5 5.5 4.5 3.5 2.5 1.5 0.5 -0.5
ppm

4f

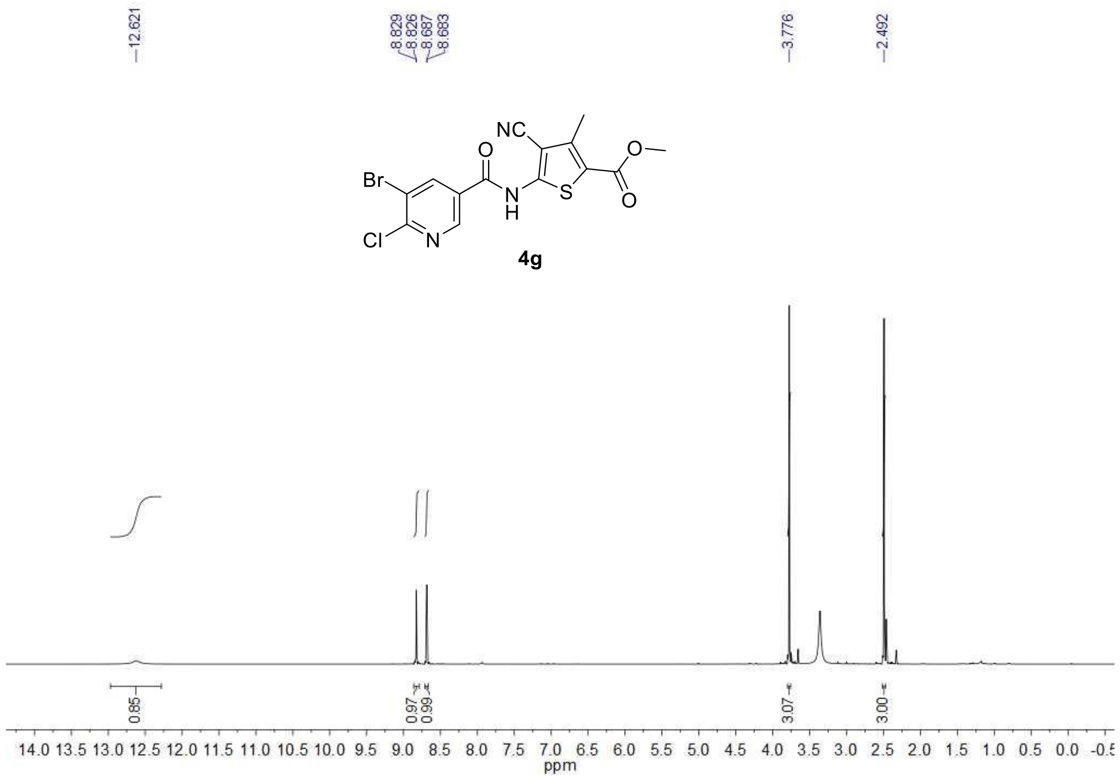
12.621
8.829
8.826
8.687
8.683
3.776
2.492
NC
O
Br
N
H
S
O
O
Cl
N
4g
0.85
0.97
0.99
3.07
3.00
14.0 13.5 13.0 12.5 12.0 11.5 11.0 10.5 10.0 9.5 9.0 8.5 8.0 7.5 7.0 6.5 6.0 5.5 5.0 4.5 4.0 3.5 3.0 2.5 2.0 1.5 1.0 0.5 0.0 -0.5
ppm

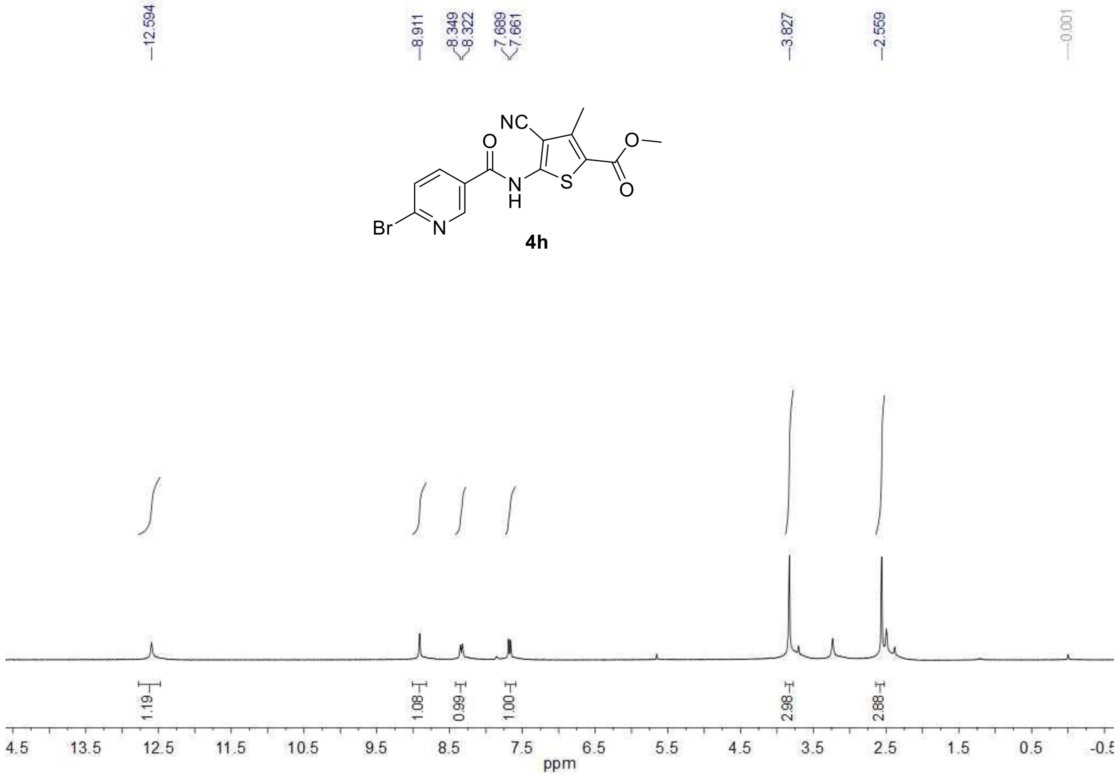
12.594
8.911
8.349
8.322
7.689
7.661
3.827
2.559
0.001
NC
O
N
H
S
O
O
Br
N
4h
1.19
1.08
0.99
1.00
2.98
2.88
13.5 12.5 11.5 10.5 9.5 8.5 7.5 6.5 5.5 4.5 3.5 2.5 1.5 0.5 -0.5
ppm

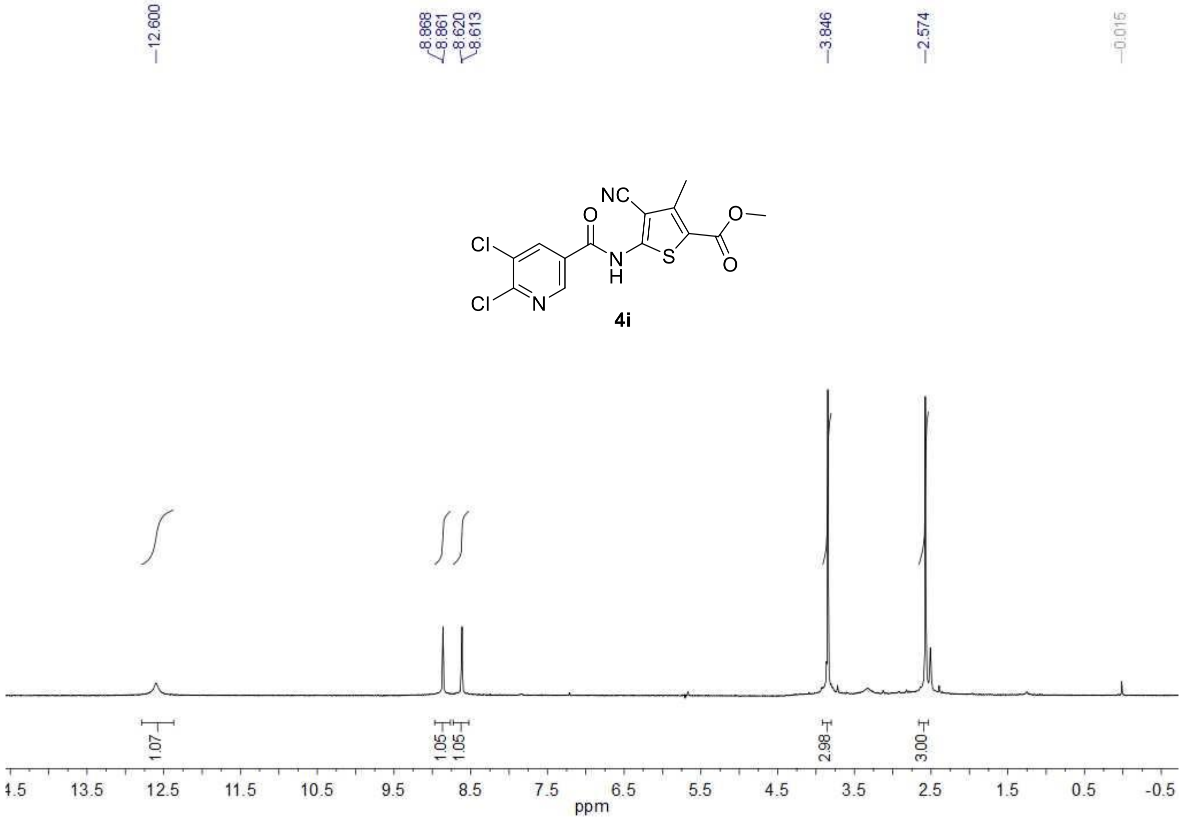
12.600
8.868
8.861
8.620
8.613
3.846
2.574
0.015
NC
O
O
O
Cl
Cl
N
NH
S
4i
1.07
1.05
1.05
2.98
3.00
ppm

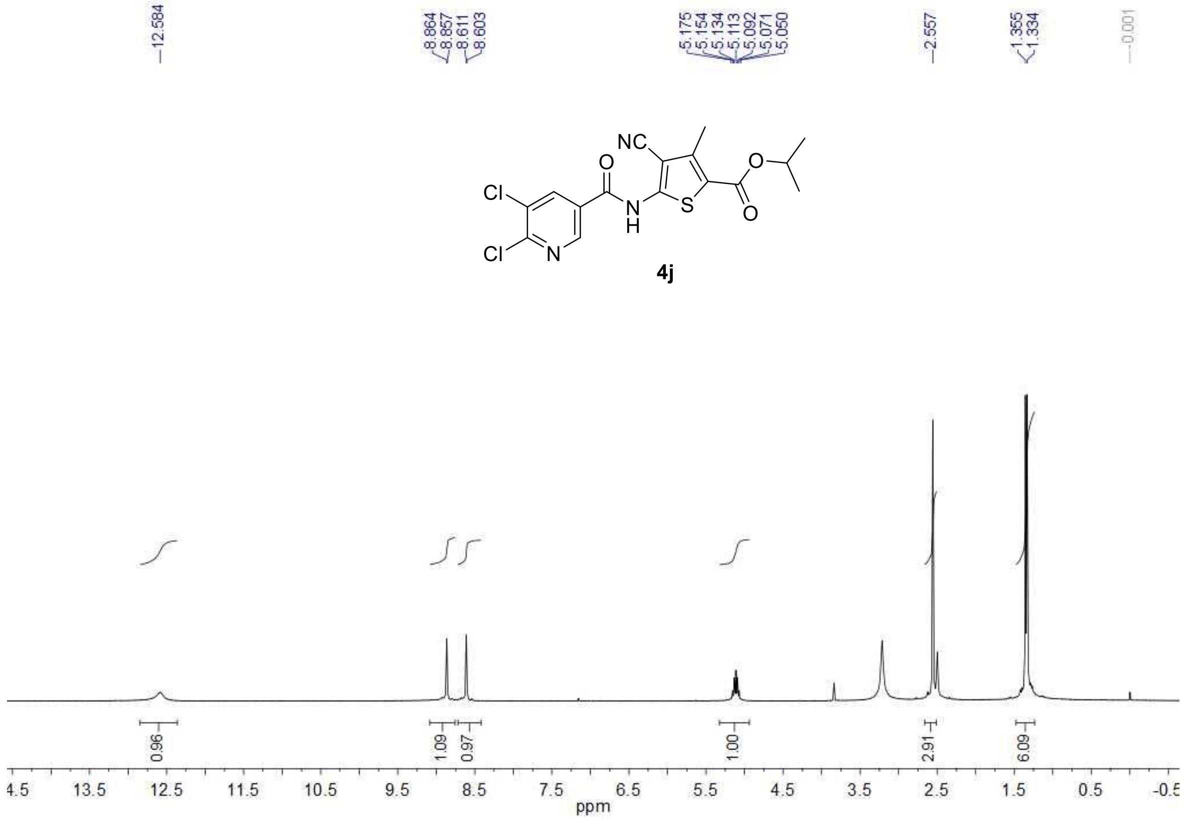
12.584
8.864
8.857
8.611
8.603
5.175
5.154
5.134
5.113
5.092
5.071
5.050
2.557
1.355
1.334
0.001
NC
O
O
O
Cl
Cl
N
NH
S
4j
0.96
1.09
0.97
1.00
2.91
6.09
ppm

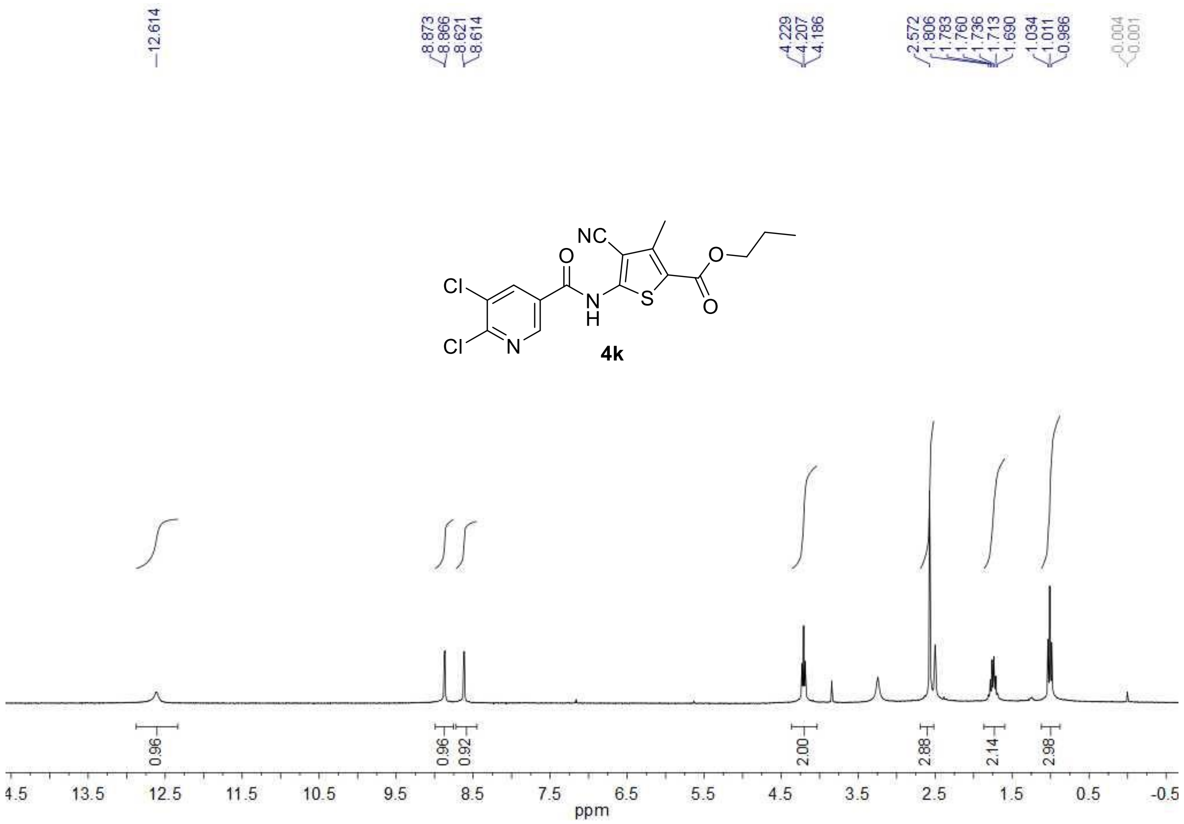
12.614
8.873
8.866
8.621
8.614
4.229
4.207
4.186
2.572
1.806
1.783
1.760
1.736
1.713
1.690
1.034
1.011
0.986
0.004
0.001
NC
O
Cl
Cl
N
N
H
S
O
O
4k
0.96
0.96
0.92
2.00
2.88
2.14
2.98
ppm

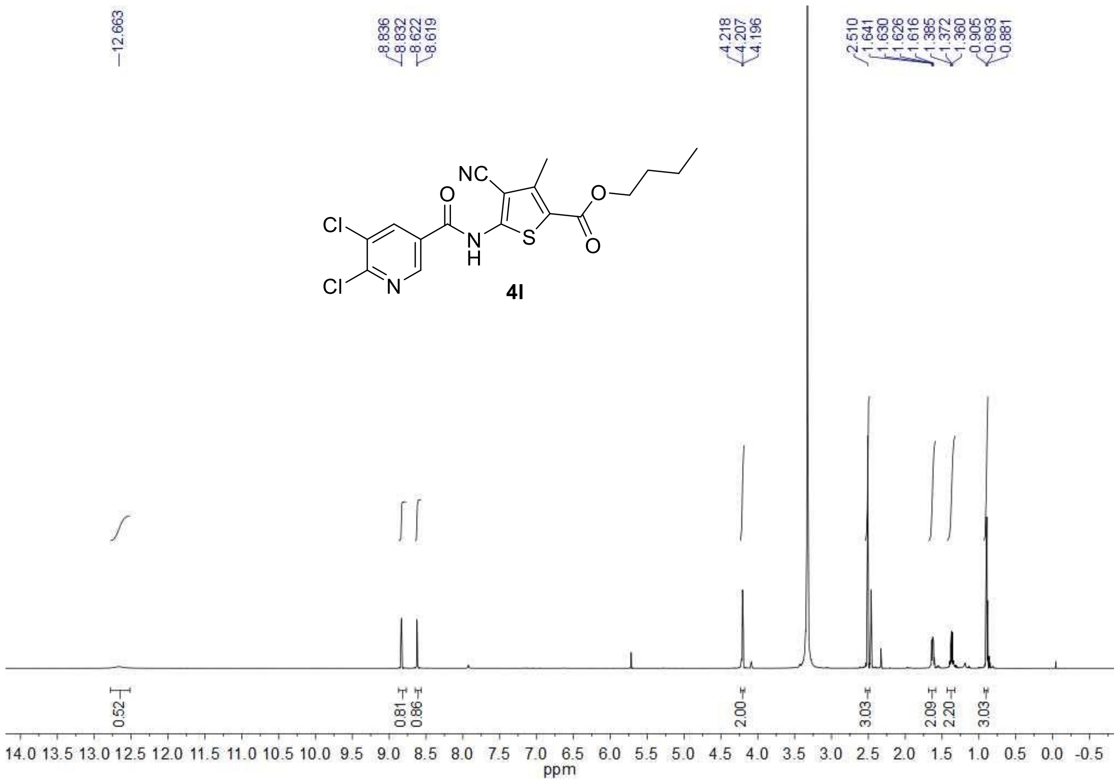
12.663
8.836
8.832
8.622
8.619
4.218
4.207
4.196
2.510
1.641
1.630
1.626
1.616
1.385
1.372
1.360
0.905
0.893
0.881
NC
O
Cl
Cl
N
N
H
S
O
O
4l
0.52
0.81
0.86
2.00
3.03
2.09
2.20
3.03
ppm

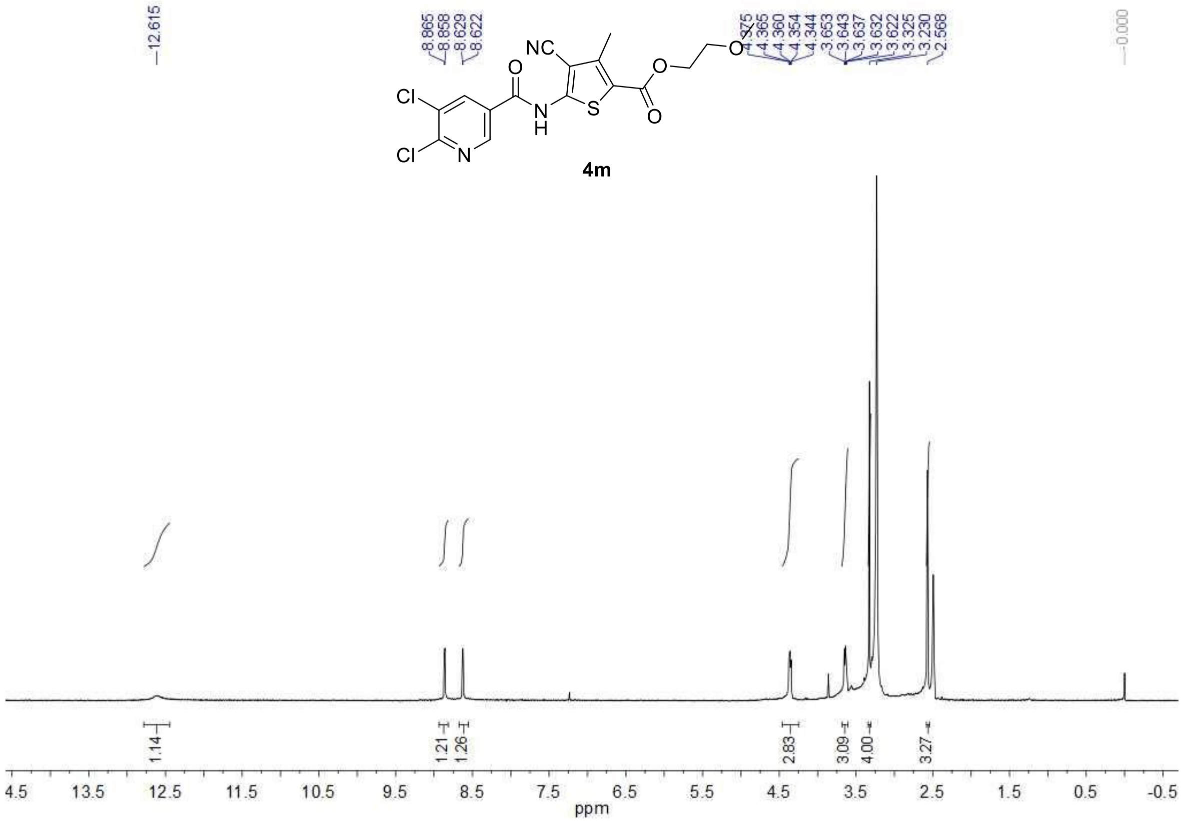
12.615
8.865
8.858
8.629
8.622
4.375
4.365
4.360
4.354
4.344
3.653
3.643
3.637
3.632
3.622
3.325
3.230
2.568
0.000
NC
O
Cl
Cl
N
N
H
S
O
O
O
4m
1.14
1.21
1.26
2.83
3.09
4.00
3.27
ppm

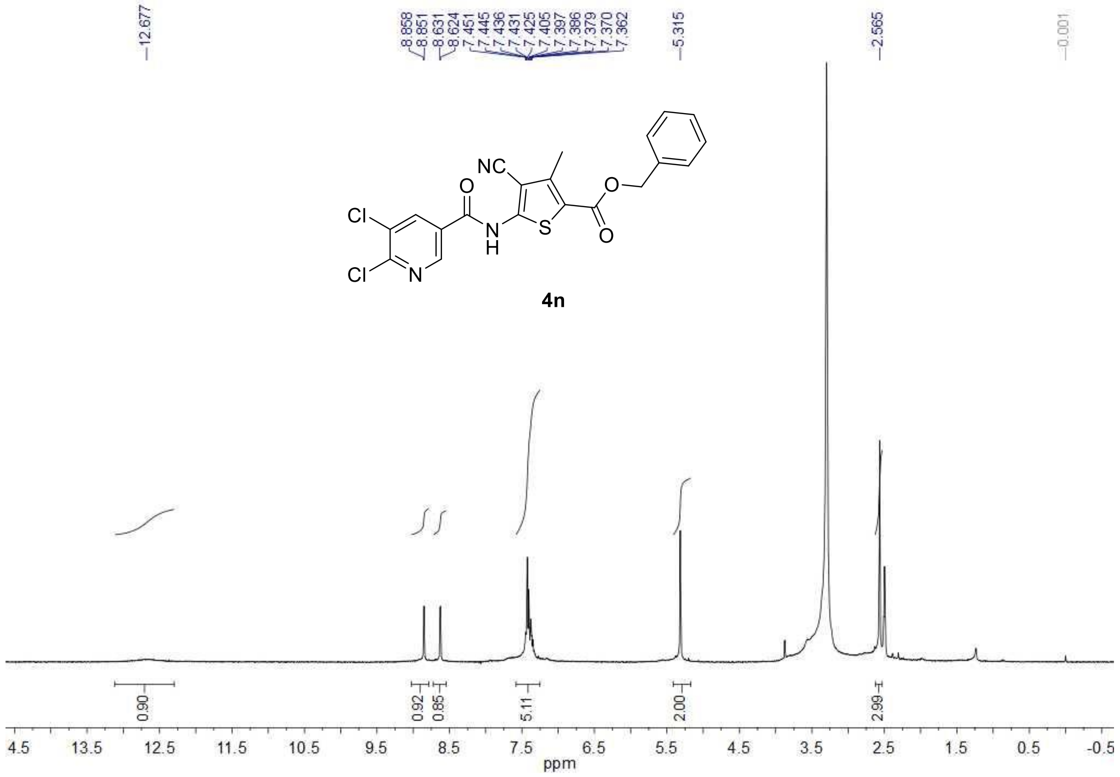
12.677
8.858
8.851
8.631
8.624
7.451
7.445
7.436
7.431
7.425
7.405
7.397
7.386
7.379
7.370
7.362
5.315
2.565
-0.001
NC
O
Cl
Cl
N
N
H
S
O
O
4n
0.90
0.92
0.85
5.11
2.00
2.99
ppm

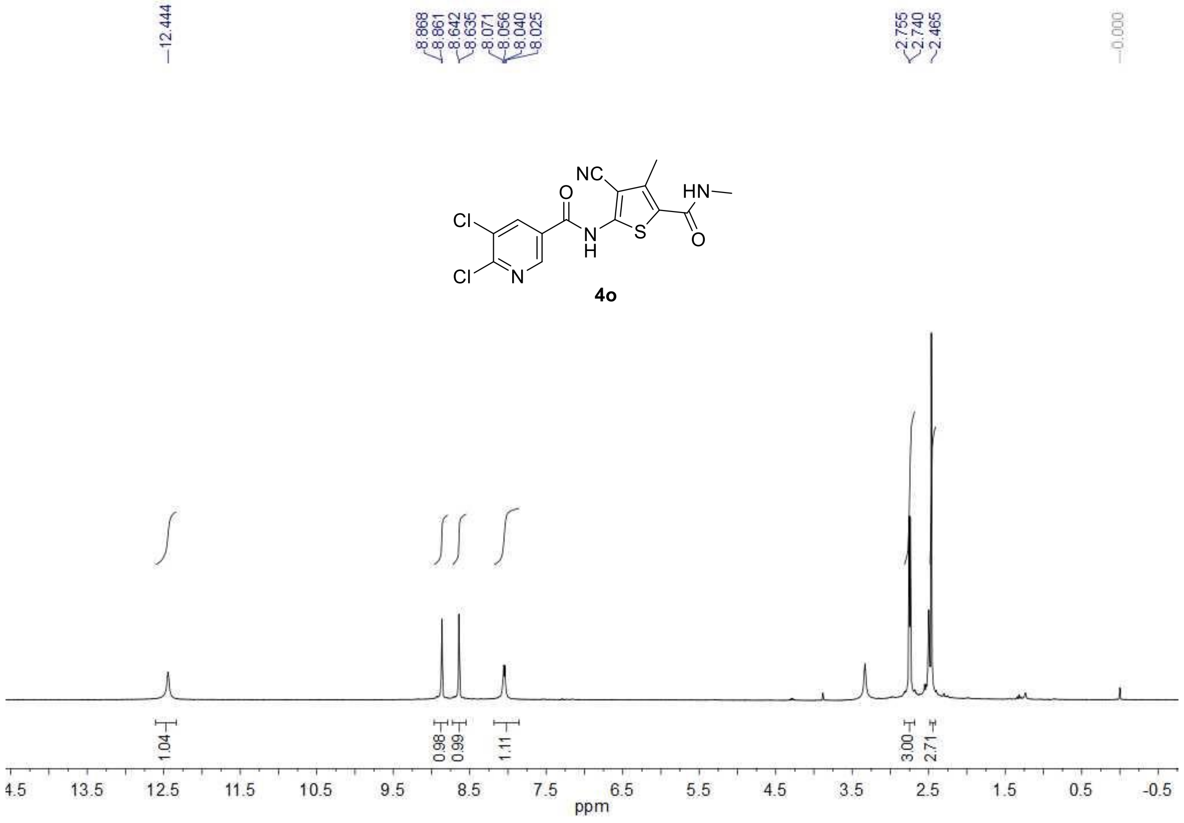
12.444
8.868
8.861
8.642
8.635
8.071
8.056
8.040
8.025
2.755
2.740
2.465
0.000
NC
HN
Cl
O
N
H
S
O
Cl
N
1.04
0.98
0.99
1.11
3.00
2.71
ppm

4o

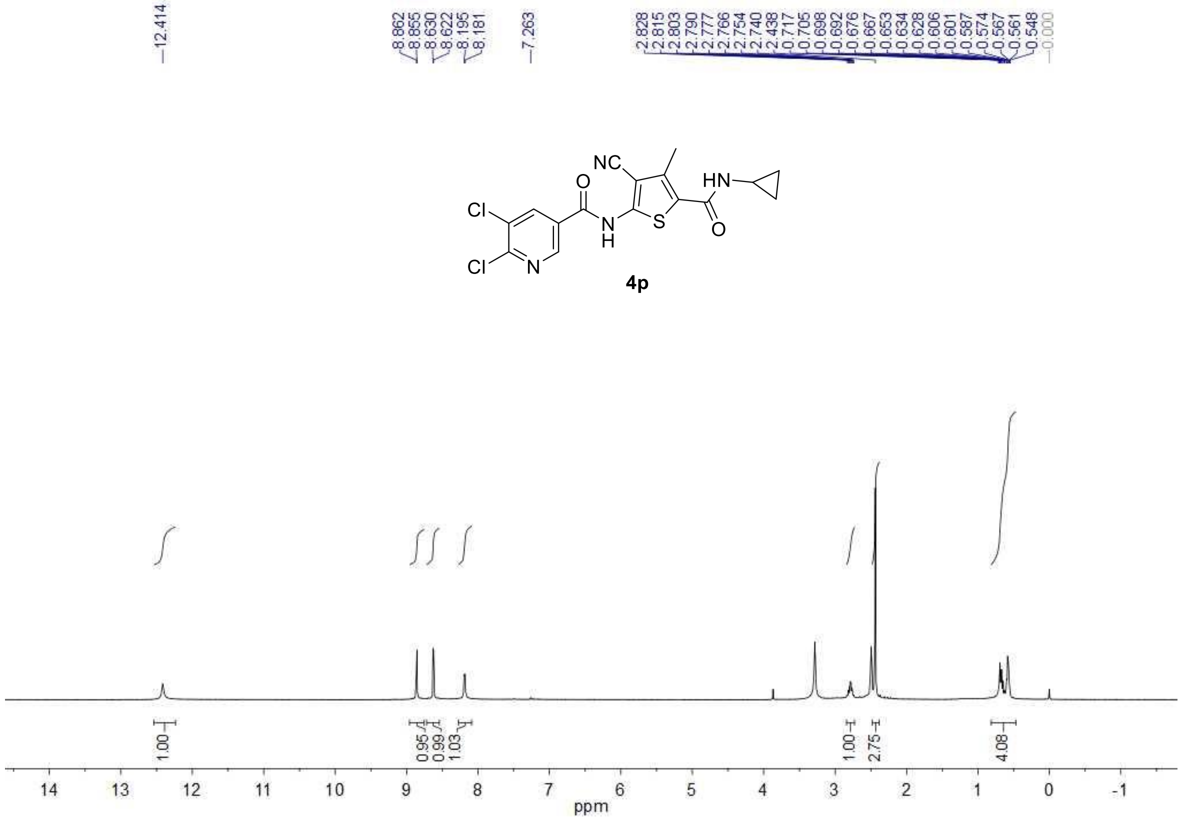
12.414
8.862
8.855
8.630
8.622
8.195
8.181
7.263
2.828
2.815
2.803
2.790
2.777
2.766
2.754
2.740
2.438
0.717
0.705
0.698
0.692
0.676
0.667
0.653
0.634
0.628
0.606
0.601
0.587
0.574
0.567
0.561
0.548
0.000
NC
HN
Cl
O
N
H
S
O
Cl
N
1.00
0.95
0.99
1.03
1.00
2.75
4.08
ppm

4p

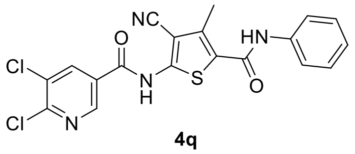

NC
HN
O
Cl
N
H
S
O
Cl
N

4q

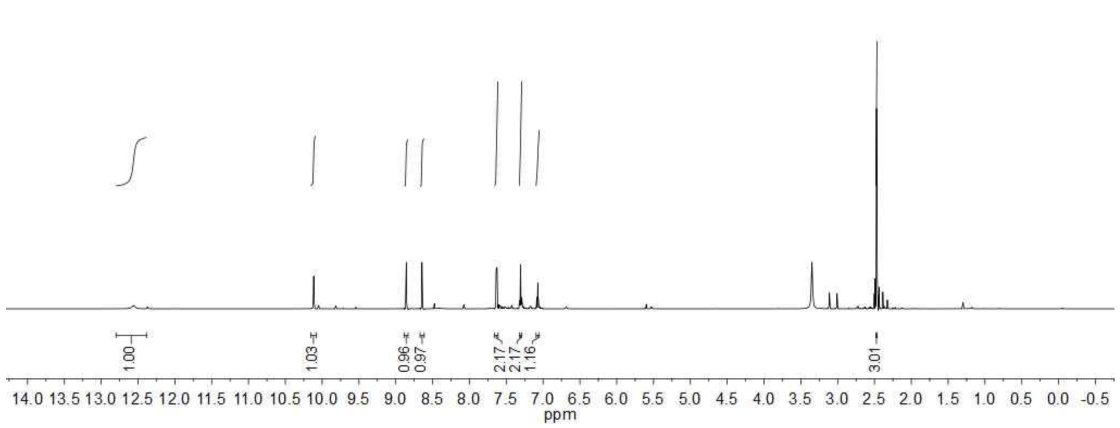

12.560
10.114
8.860
8.857
8.645
8.641
7.640
7.638
7.634
7.628
7.625
7.623
7.319
7.316
7.307
7.305
7.295
7.293
7.086
7.084
7.082
7.074
7.071
7.069
7.061
7.059
7.057
2.472
1.00
1.03
0.96
0.97
2.17
2.17
1.16
3.01
14.0 13.5 13.0 12.5 12.0 11.5 11.0 10.5 10.0 9.5 9.0 8.5 8.0 7.5 7.0 6.5 6.0 5.5 5.0 4.5 4.0 3.5 3.0 2.5 2.0 1.5 1.0 0.5 0.0 -0.5
ppm

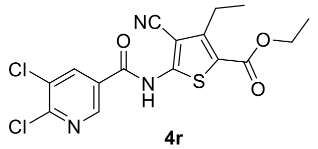

NC
O
O
Cl
N
H
S
O
Cl
N

4r

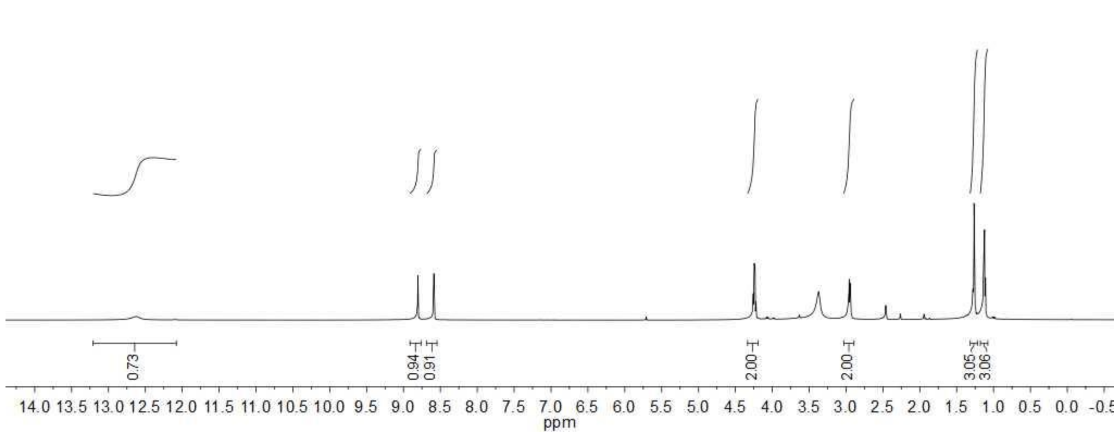

12.628
8.805
8.801
8.588
8.585
4.257
4.245
4.234
4.222
2.970
2.957
2.945
2.933
1.276
1.264
1.252
1.134
1.122
1.110
0.73
0.94
0.91
2.00
2.00
3.05
3.06
14.0 13.5 13.0 12.5 12.0 11.5 11.0 10.5 10.0 9.5 9.0 8.5 8.0 7.5 7.0 6.5 6.0 5.5 5.0 4.5 4.0 3.5 3.0 2.5 2.0 1.5 1.0 0.5 0.0
ppm

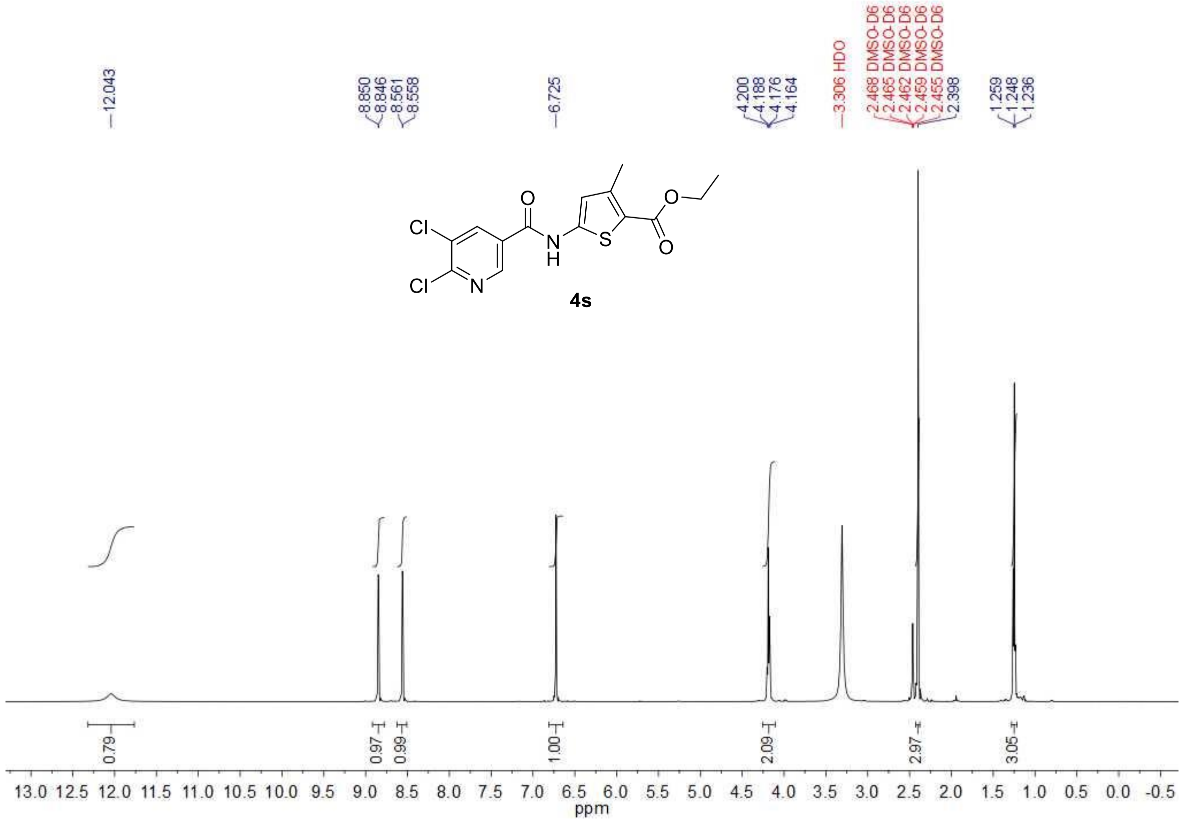

12.043
8.850
8.846
8.561
8.558
6.725
4.200
4.188
4.176
4.164
3.306 HDO
2.468 DMSO-D6
2.465 DMSO-D6
2.462 DMSO-D6
2.459 DMSO-D6
2.455 DMSO-D6
2.398
1.259
1.248
1.236
4s
0.79
0.97
0.99
1.00
2.09
2.97
3.05
13.0 12.5 12.0 11.5 11.0 10.5 10.0 9.5 9.0 8.5 8.0 7.5 7.0 6.5 6.0 5.5 5.0 4.5 4.0 3.5 3.0 2.5 2.0 1.5 1.0 0.5 0.0 -0.5
ppm

^{13}C NMR SPECTRAL GRAPH

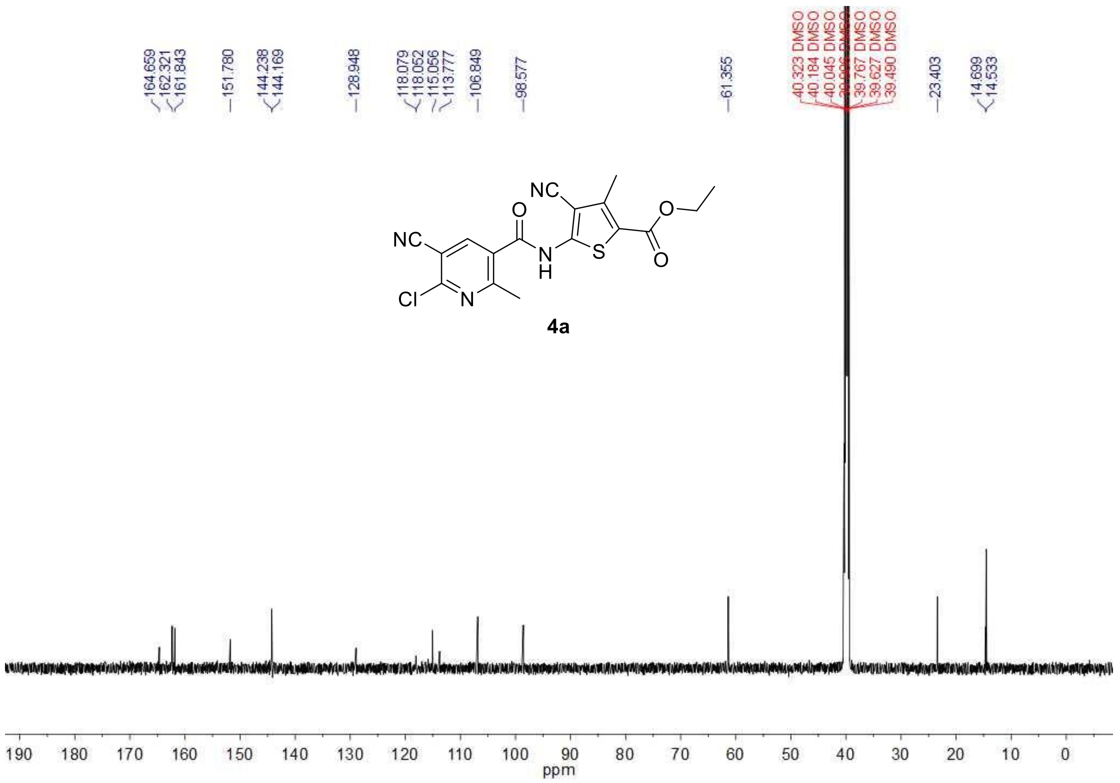

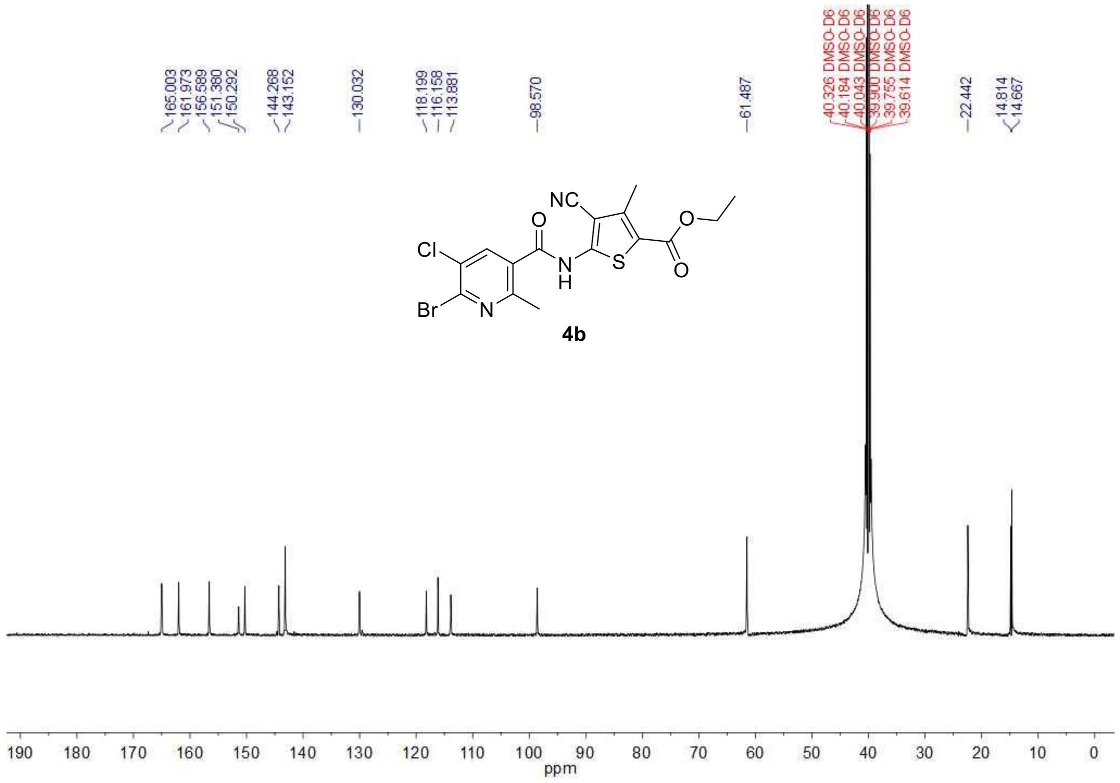

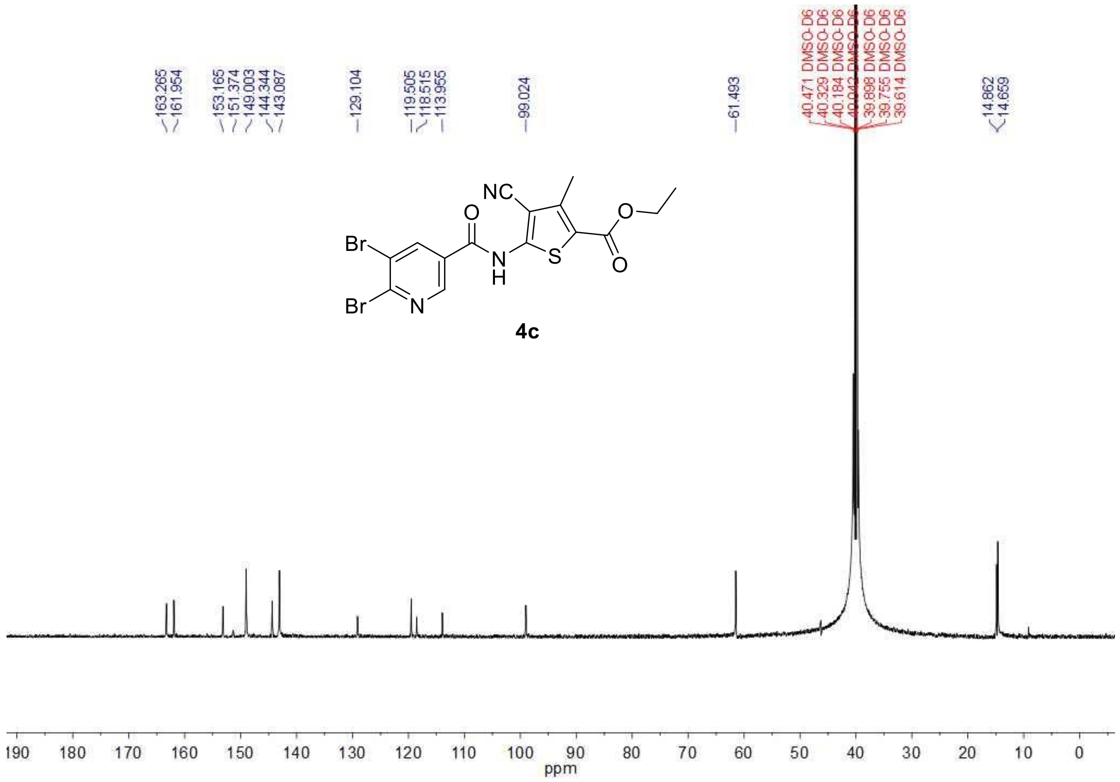
163.265
161.954
153.165
151.374
149.003
144.344
143.087
129.104
119.505
118.515
113.955
99.024
61.493
40.471 DMSO-D6
40.329 DMSO-D6
40.184 DMSO-D6
40.042 DMSO-D6
39.898 DMSO-D6
39.755 DMSO-D6
39.614 DMSO-D6
14.862
14.659
NC
O
O
Br
N
H
S
O
Br
N
ppm

4c

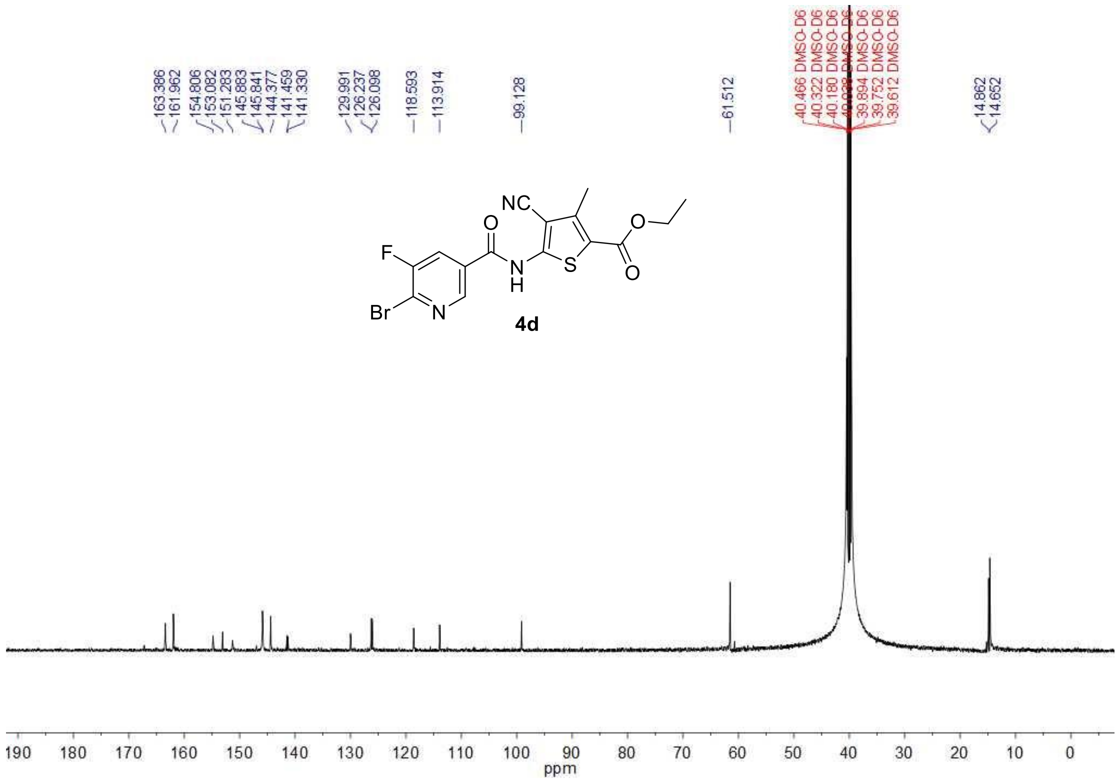
163.386
161.962
154.806
153.082
151.283
145.883
145.841
144.377
141.459
141.330
129.991
126.237
126.098
118.593
113.914
99.128
61.512
40.466 DMSO-D6
40.322 DMSO-D6
40.180 DMSO-D6
40.038 DMSO-D6
39.894 DMSO-D6
39.752 DMSO-D6
39.612 DMSO-D6
14.862
14.652
NC
O
O
F
N
H
S
O
Br
N
ppm

4d

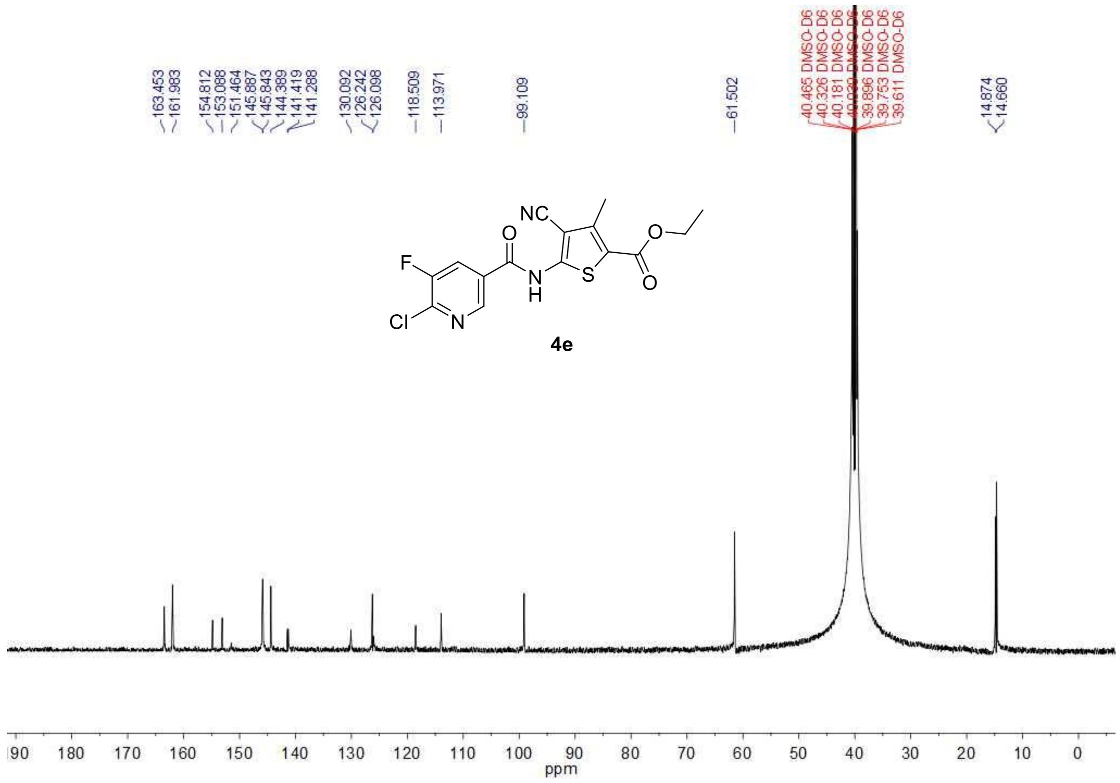
163.453
161.983
154.812
153.088
151.464
145.887
145.843
144.389
141.419
141.288
130.092
126.242
126.098
118.509
113.971
99.109
61.502
40.465 DMSO-D6
40.326 DMSO-D6
40.181 DMSO-D6
39.896 DMSO-D6
39.753 DMSO-D6
39.611 DMSO-D6
14.874
14.660
NC
O
F
NH
S
O
Cl
N
190 180 170 160 150 140 130 120 110 100 90 80 70 60 50 40 30 20 10 0
ppm

4e

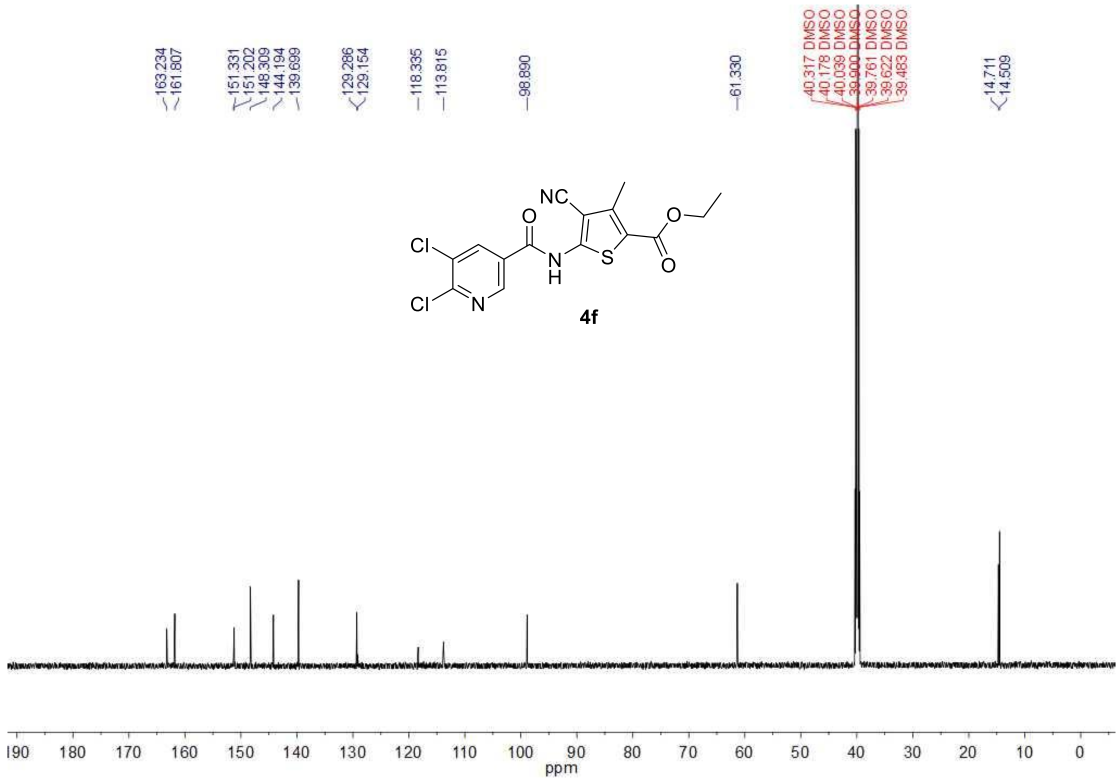
163.234
161.807
151.331
151.202
148.309
144.194
139.699
129.286
129.154
118.335
113.815
98.890
61.330
40.317 DMSO
40.178 DMSO
40.039 DMSO
39.900 DMSO
39.761 DMSO
39.622 DMSO
39.483 DMSO
14.711
14.509
NC
O
Cl
NH
S
O
Cl
N
190 180 170 160 150 140 130 120 110 100 90 80 70 60 50 40 30 20 10 0
ppm

4f

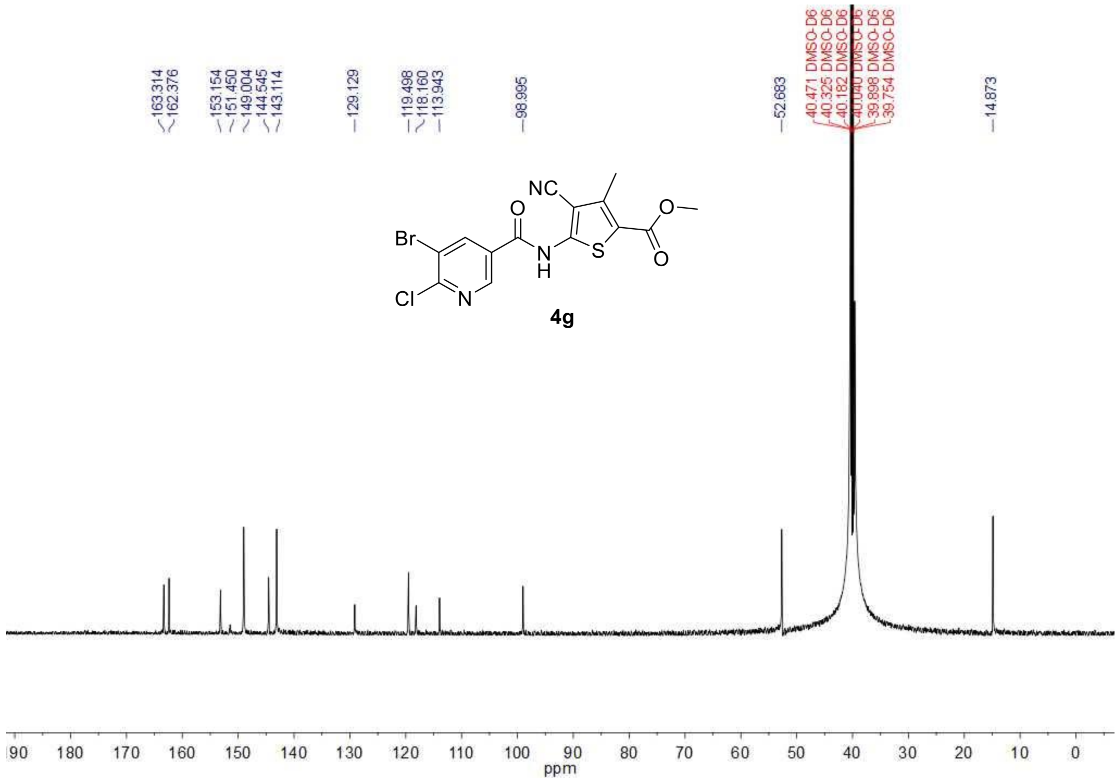
163.314
162.376
153.154
151.450
149.004
144.545
143.114
129.129
119.498
118.160
113.943
98.995
52.683
40.471 DMSO-D6
40.325 DMSO-D6
40.182 DMSO-D6
40.040 DMSO-D6
39.898 DMSO-D6
39.754 DMSO-D6
14.873
NC
O
O
Br
N
H
S
O
Cl
N
4g
190 180 170 160 150 140 130 120 110 100 90 80 70 60 50 40 30 20 10 0
ppm

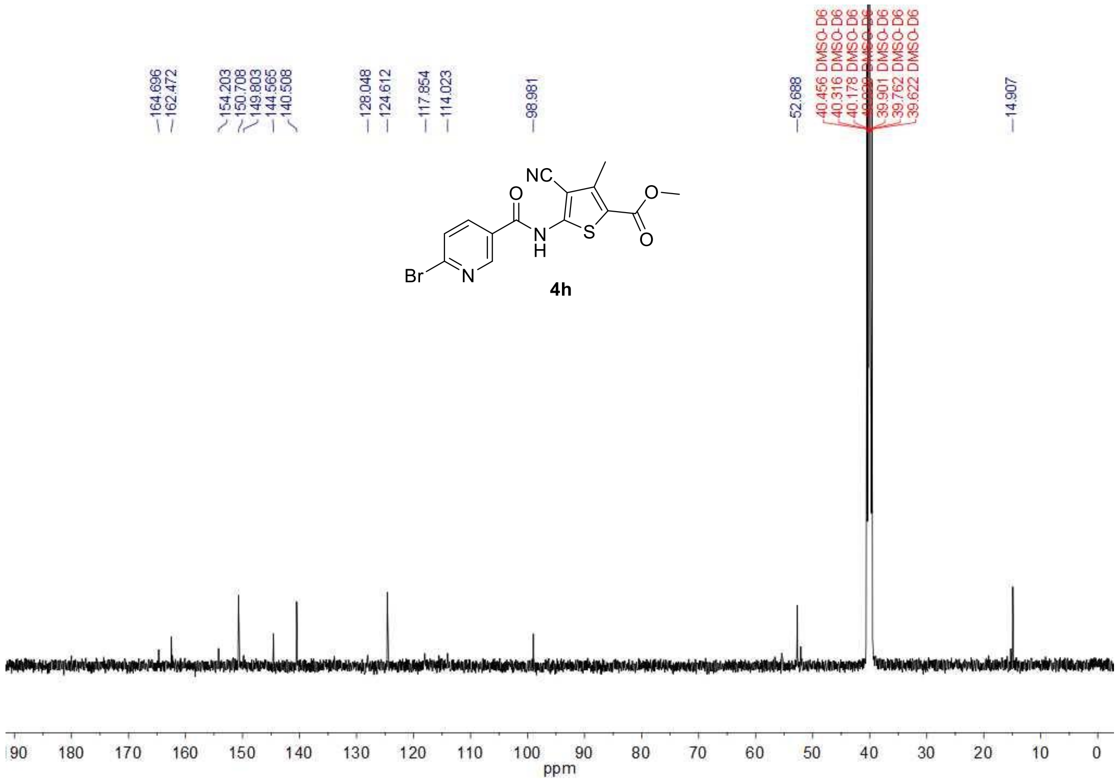
164.696
162.472
154.203
150.708
149.803
144.565
140.508
128.048
124.612
117.854
114.023
98.981
52.688
40.456 DMSO-D6
40.316 DMSO-D6
40.178 DMSO-D6
39.901 DMSO-D6
39.762 DMSO-D6
39.622 DMSO-D6
14.907
NC
O
O
N
H
S
O
Br
N
4h
190 180 170 160 150 140 130 120 110 100 90 80 70 60 50 40 30 20 10 0
ppm

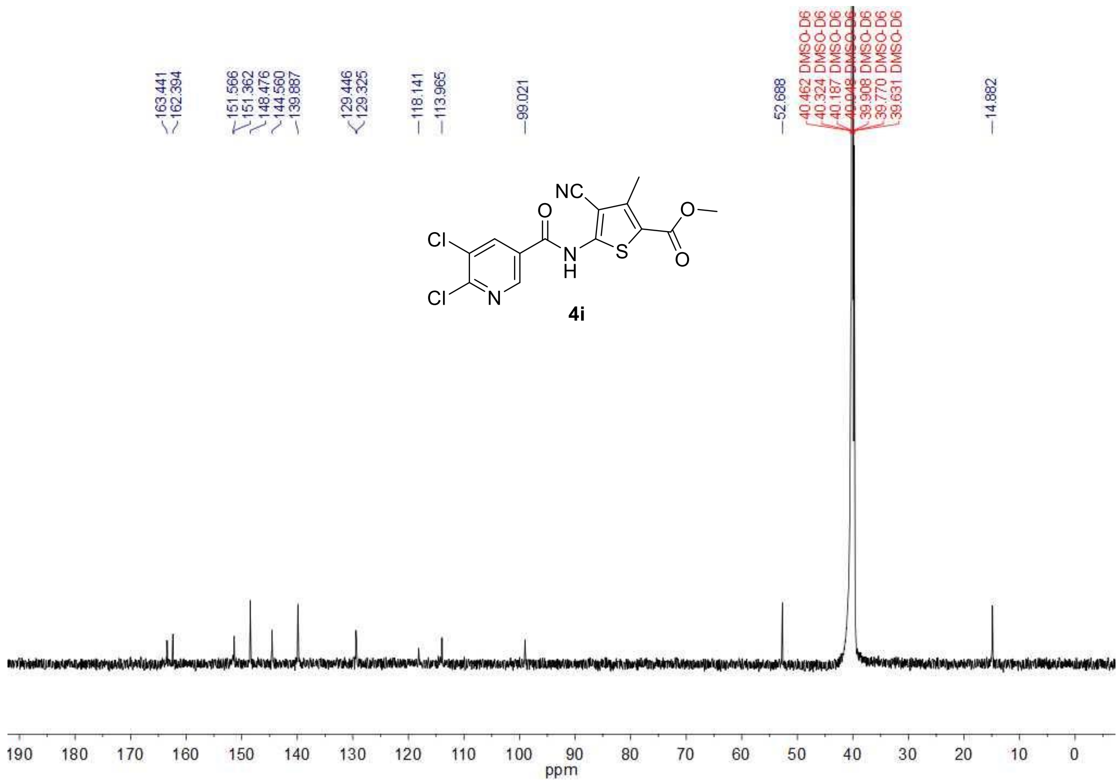
163.441
162.394
151.566
151.362
148.476
144.560
139.887
129.446
129.325
118.141
113.965
99.021
52.688
40.462 DMSO-D6
40.324 DMSO-D6
40.187 DMSO-D6
40.048 DMSO-D6
39.908 DMSO-D6
39.770 DMSO-D6
39.631 DMSO-D6
14.882
NC
O
Cl
N
H
S
O
O
Cl
N
4i
190 180 170 160 150 140 130 120 110 100 90 80 70 60 50 40 30 20 10 0
ppm

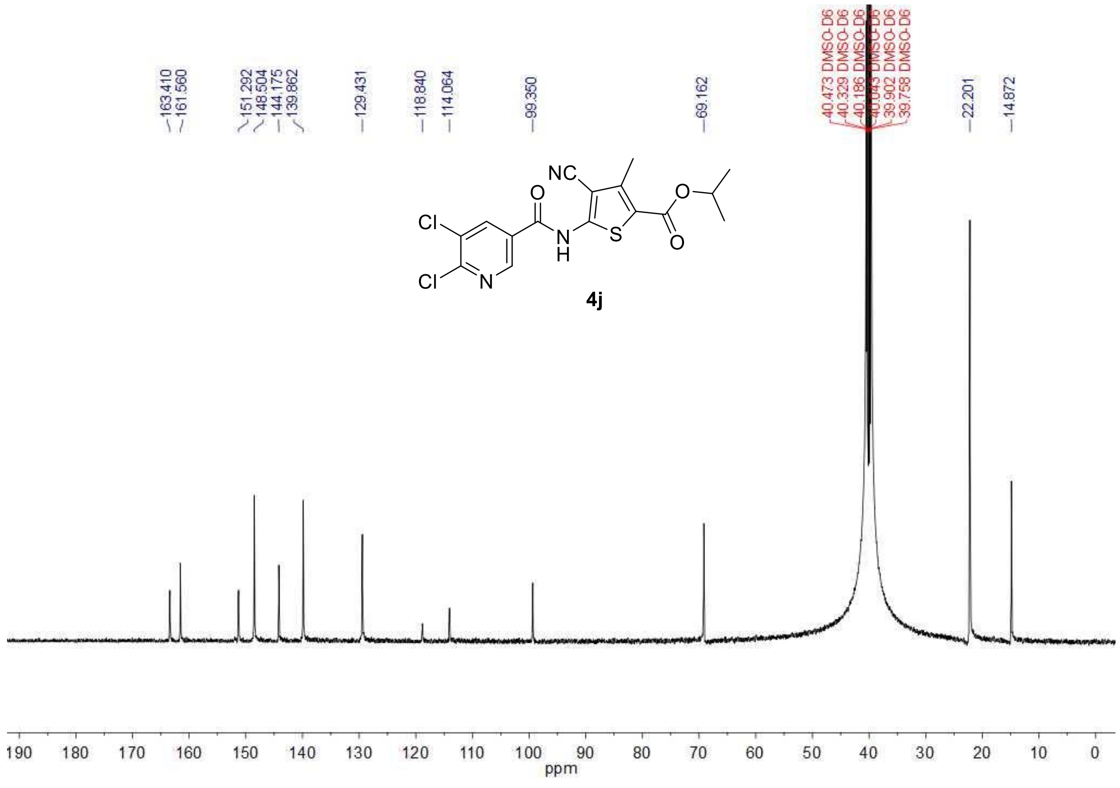
163.410
161.560
151.292
148.504
144.175
139.862
129.431
118.840
114.064
99.350
69.162
40.473 DMSO-D6
40.329 DMSO-D6
40.186 DMSO-D6
40.045 DMSO-D6
39.902 DMSO-D6
39.758 DMSO-D6
22.201
14.872
NC
O
Cl
N
H
S
O
O
Cl
N
4j
190 180 170 160 150 140 130 120 110 100 90 80 70 60 50 40 30 20 10 0
ppm

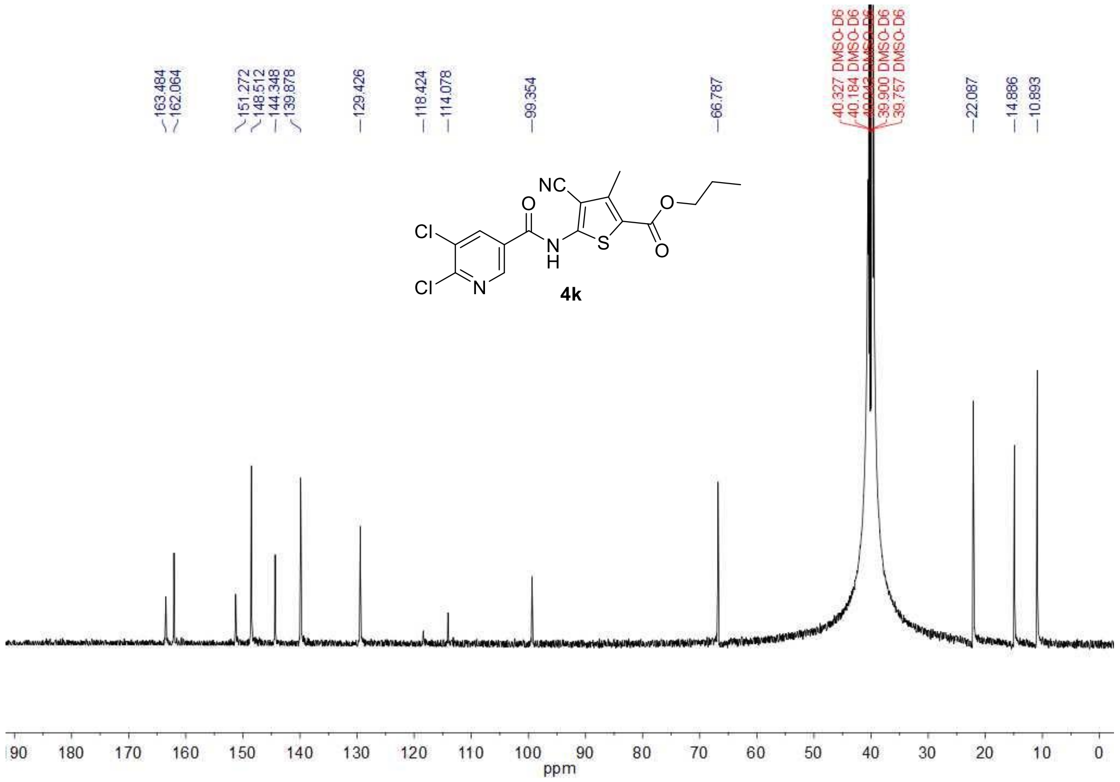
163.484
162.064
151.272
148.512
144.348
139.878
129.426
118.424
114.078
99.354
66.787
40.327 DMSO-D6
40.184 DMSO-D6
39.900 DMSO-D6
39.757 DMSO-D6
22.087
14.886
10.893
NC
Cl
Cl
N
O
N
H
S
O
O
4k
ppm

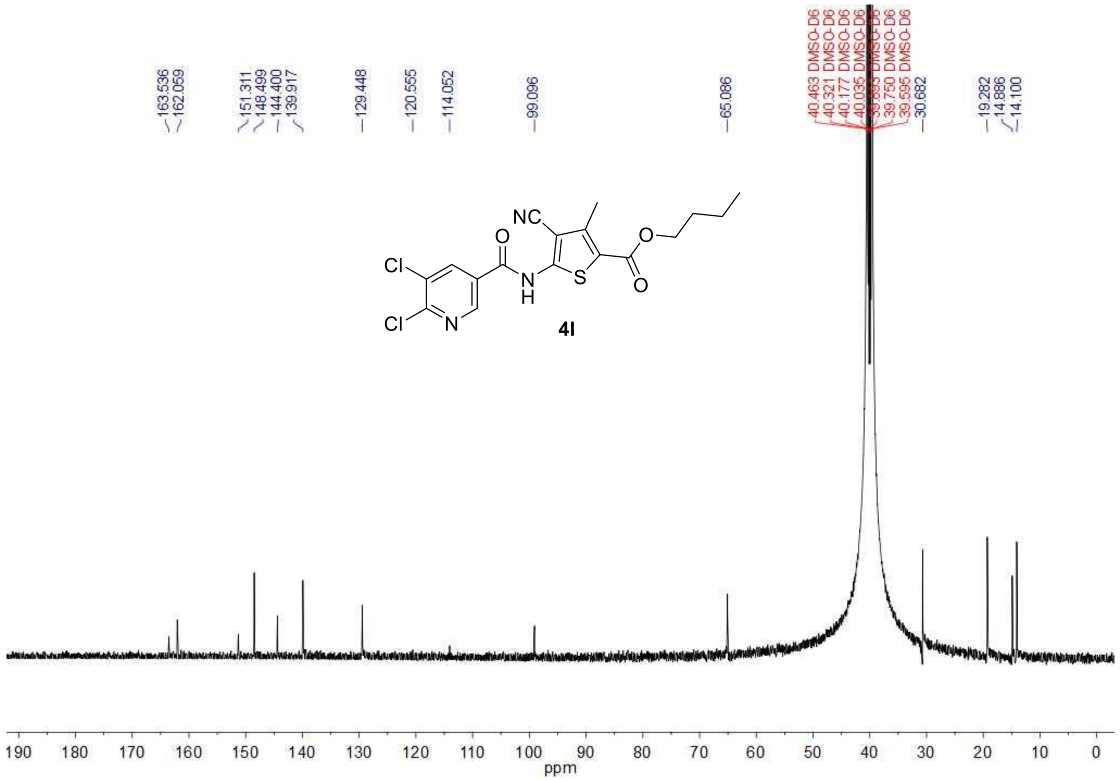
163.536
162.059
151.311
148.499
144.400
139.917
129.448
120.555
114.052
99.096
65.086
40.463 DMSO-D6
40.321 DMSO-D6
40.177 DMSO-D6
40.035 DMSO-D6
39.750 DMSO-D6
39.595 DMSO-D6
30.682
19.282
14.886
14.100
NC
Cl
Cl
N
O
N
H
S
O
O
4l
ppm

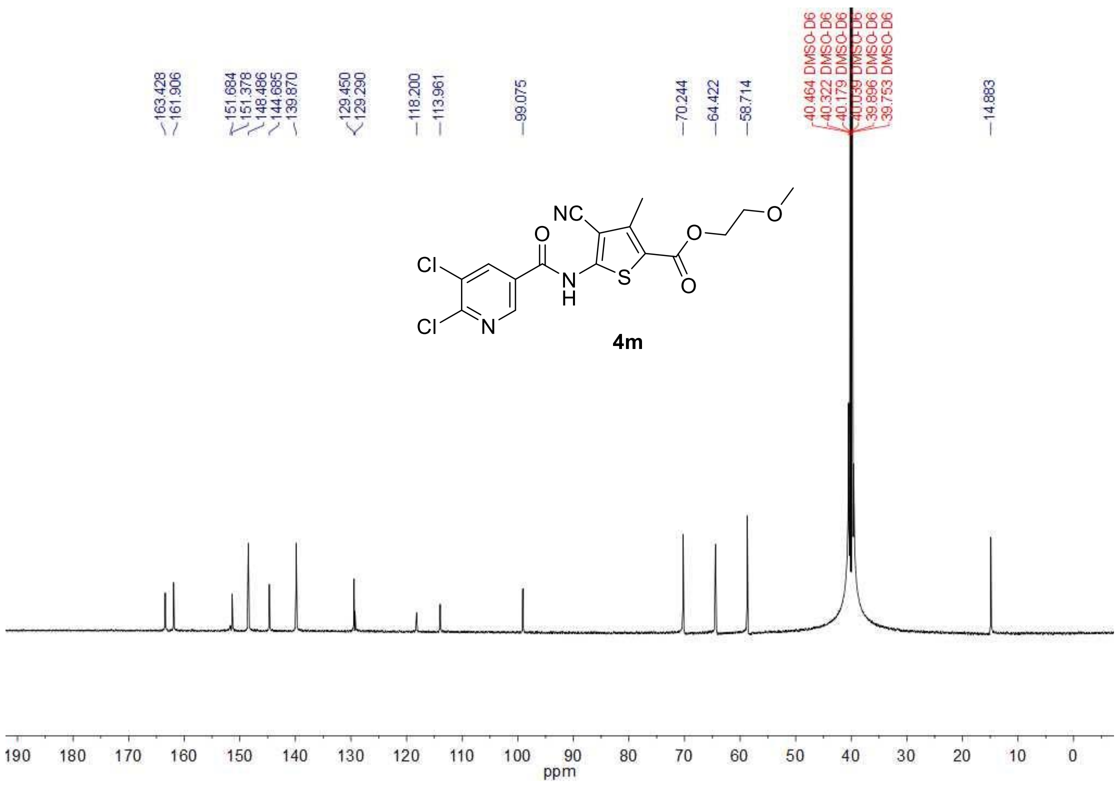
163.428
161.906
151.684
151.378
148.486
144.685
139.870
129.450
129.290
118.200
113.961
99.075
70.244
64.422
58.714
40.464 DMSO-D6
40.322 DMSO-D6
40.179 DMSO-D6
40.039 DMSO-D6
39.896 DMSO-D6
39.753 DMSO-D6
14.883
NC
O
O
Cl
N
H
S
O
Cl
N
190 180 170 160 150 140 130 120 110 100 90 80 70 60 50 40 30 20 10 0
ppm

4m

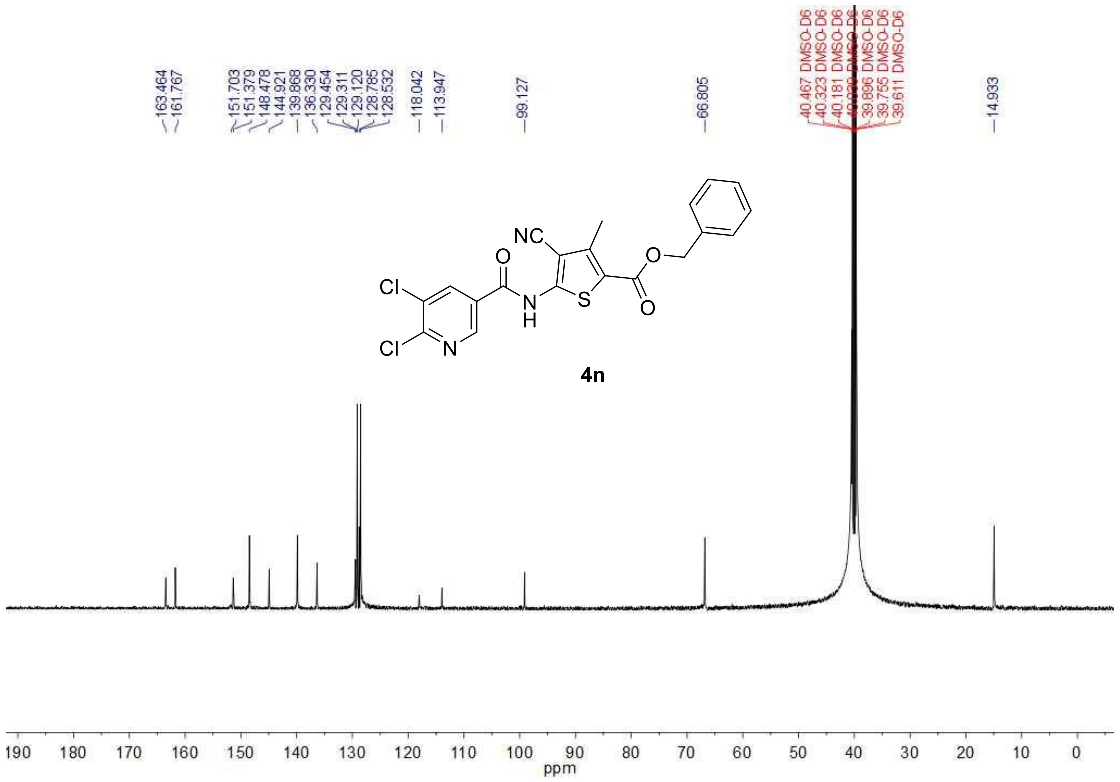
163.464
161.767
151.703
151.379
148.478
144.921
139.868
136.330
129.454
129.311
129.120
128.785
128.532
118.042
113.947
99.127
66.805
40.467 DMSO-D6
40.323 DMSO-D6
40.181 DMSO-D6
39.896 DMSO-D6
39.755 DMSO-D6
39.611 DMSO-D6
14.933
NC
O
O
Cl
N
H
S
O
Cl
N
190 180 170 160 150 140 130 120 110 100 90 80 70 60 50 40 30 20 10 0
ppm

4n

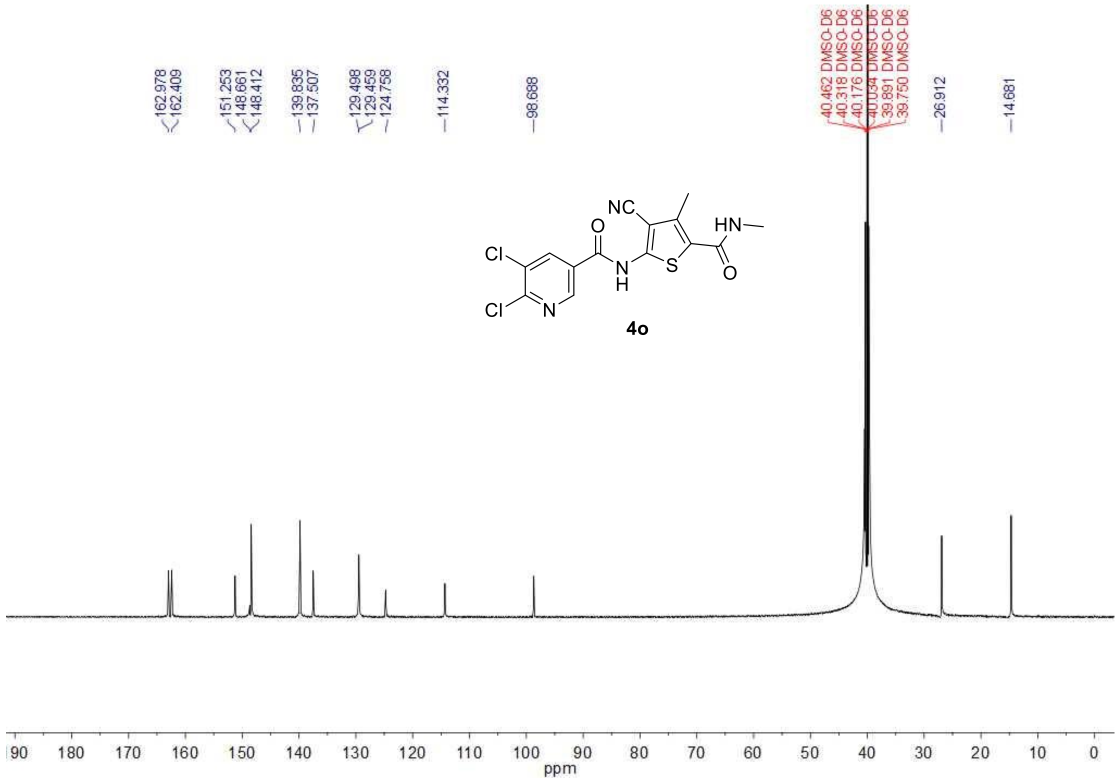
162.978
162.409
151.253
148.661
148.412
139.835
137.507
129.498
129.459
124.758
114.332
98.688
40.462 DMSO-D6
40.318 DMSO-D6
40.176 DMSO-D6
40.034 DMSO-D6
39.891 DMSO-D6
39.750 DMSO-D6
26.912
14.681
NC
HN
O
Cl
N
H
S
O
Cl
N
4o
190 180 170 160 150 140 130 120 110 100 90 80 70 60 50 40 30 20 10 0
ppm

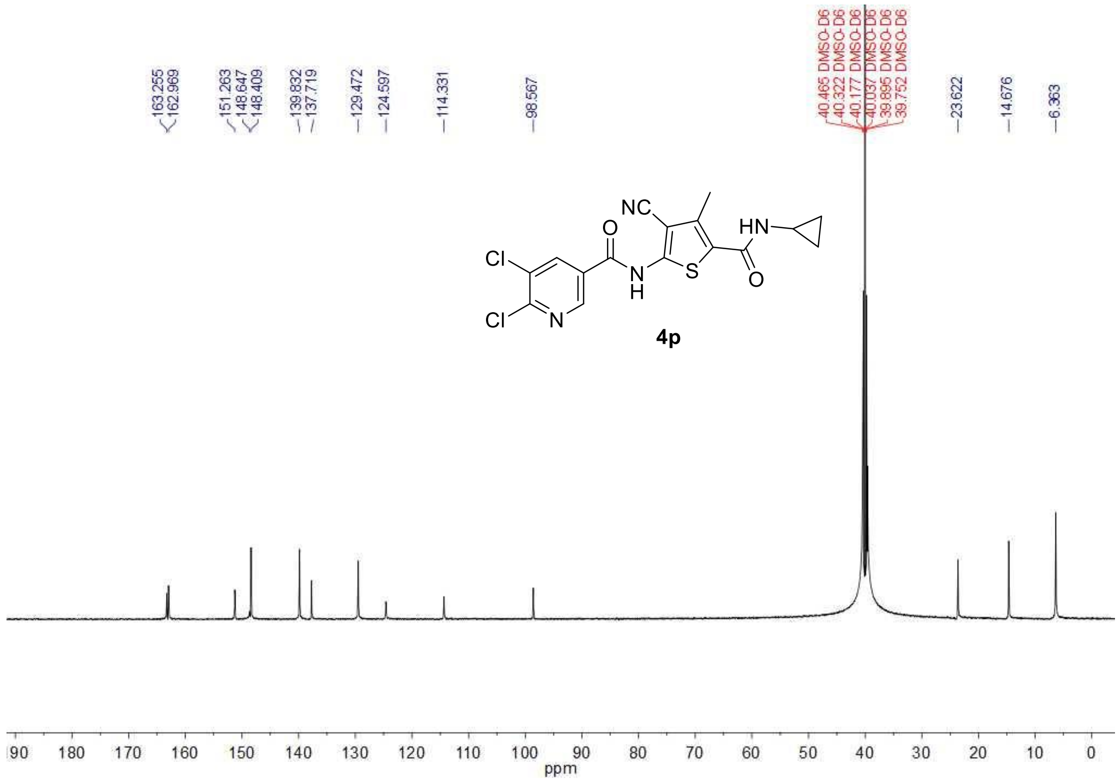
163.255
162.969
151.263
148.647
148.409
139.832
137.719
129.472
124.597
114.331
98.567
40.465 DMSO-D6
40.322 DMSO-D6
40.177 DMSO-D6
40.037 DMSO-D6
39.895 DMSO-D6
39.752 DMSO-D6
23.622
14.676
6.363
NC
HN
O
Cl
N
H
S
O
Cl
N
4p
190 180 170 160 150 140 130 120 110 100 90 80 70 60 50 40 30 20 10 0
ppm

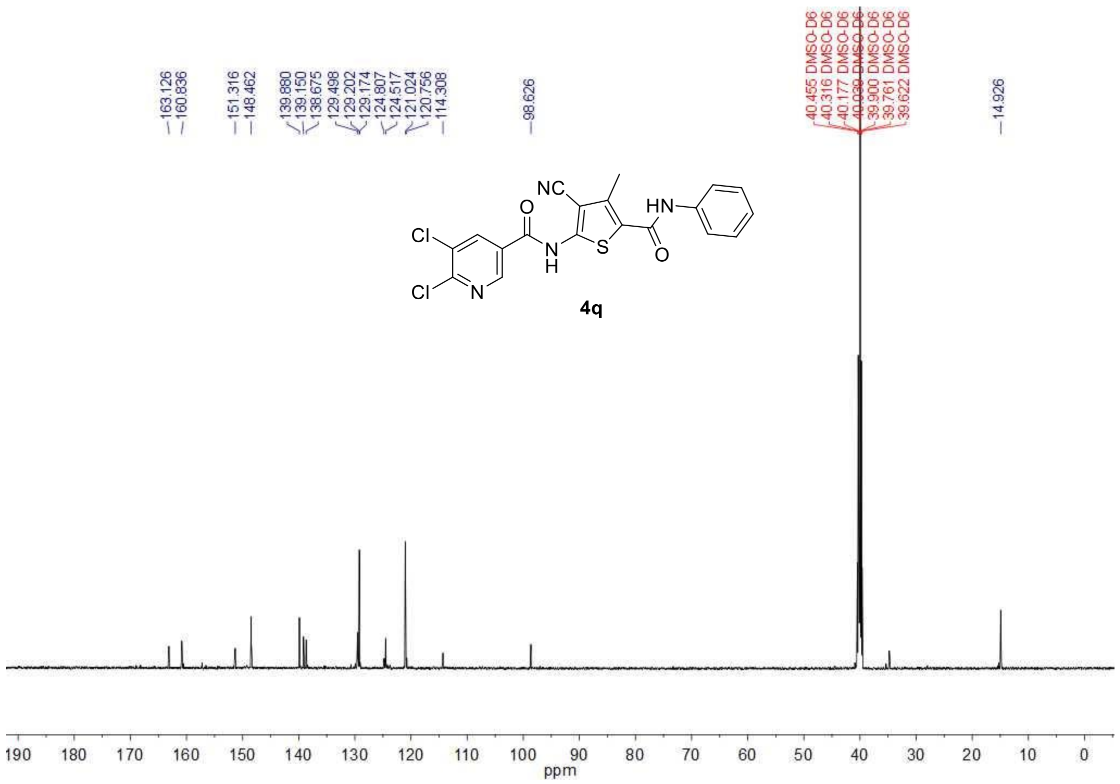
163.126
160.836
151.316
148.462
139.880
139.150
138.675
129.498
129.202
129.174
124.807
124.517
121.024
120.756
114.308
98.626
40.455 DMSO-D6
40.316 DMSO-D6
40.177 DMSO-D6
40.039 DMSO-D6
39.900 DMSO-D6
39.761 DMSO-D6
39.622 DMSO-D6
14.926
NC
HN
O
Cl
N
H
S
O
Cl
N
4q
190 180 170 160 150 140 130 120 110 100 90 80 70 60 50 40 30 20 10 0
ppm

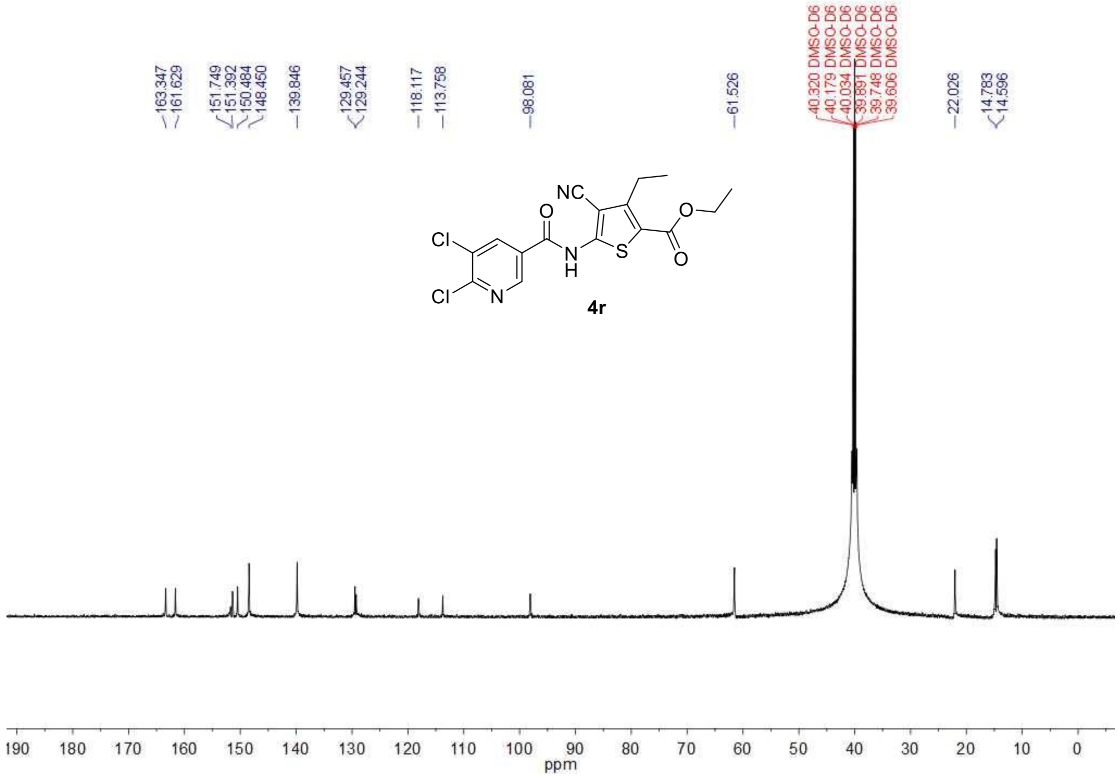
163.347
161.629
151.749
151.392
150.484
148.450
139.846
129.457
129.244
118.117
113.758
98.081
61.526
40.320 DMSO-D6
40.179 DMSO-D6
40.034 DMSO-D6
39.891 DMSO-D6
39.748 DMSO-D6
39.606 DMSO-D6
22.026
14.783
14.596
NC
O
O
Cl
N
H
S
O
Cl
N
4r
190 180 170 160 150 140 130 120 110 100 90 80 70 60 50 40 30 20 10 0
ppm

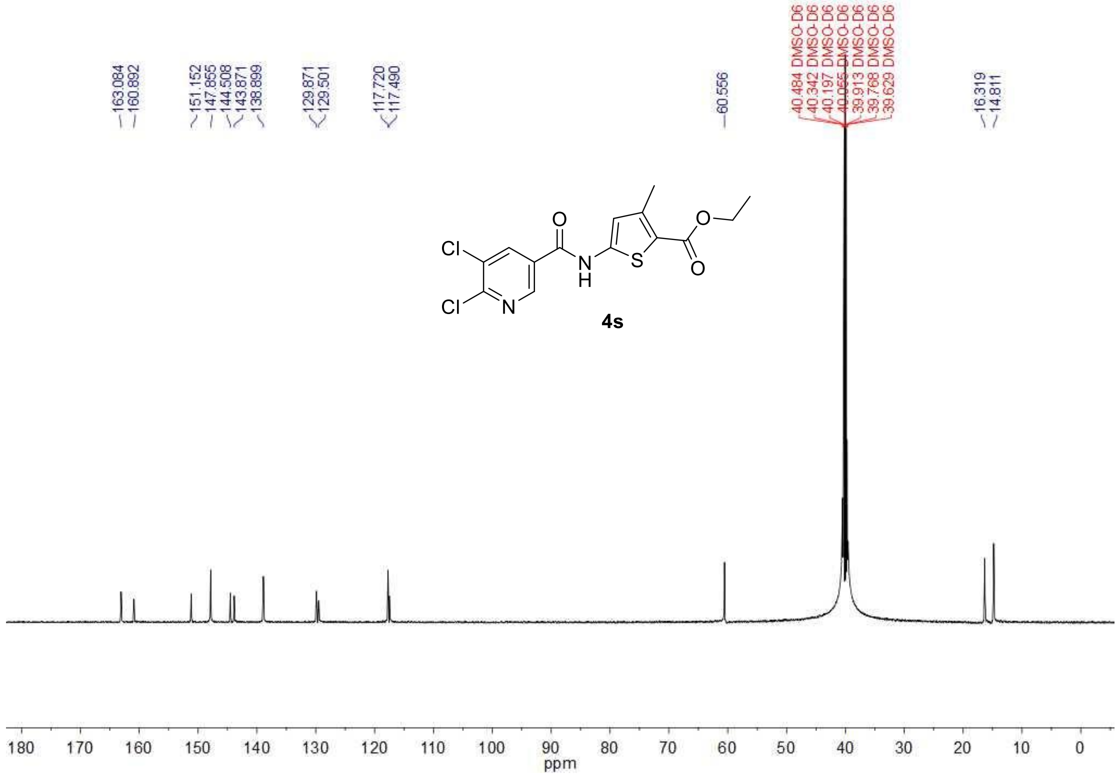
163.084
160.892
151.152
147.855
144.508
143.871
138.899
129.871
129.501
117.720
117.490
60.556
40.484 DMSO-D6
40.342 DMSO-D6
40.197 DMSO-D6
40.055 DMSO-D6
39.913 DMSO-D6
39.768 DMSO-D6
39.629 DMSO-D6
16.319
14.811
Cl
Cl
N
O
N
H
S
O
O
4s
180 170 160 150 140 130 120 110 100 90 80 70 60 50 40 30 20 10 0
ppm

HR-MS SPECTRAL GRAPH

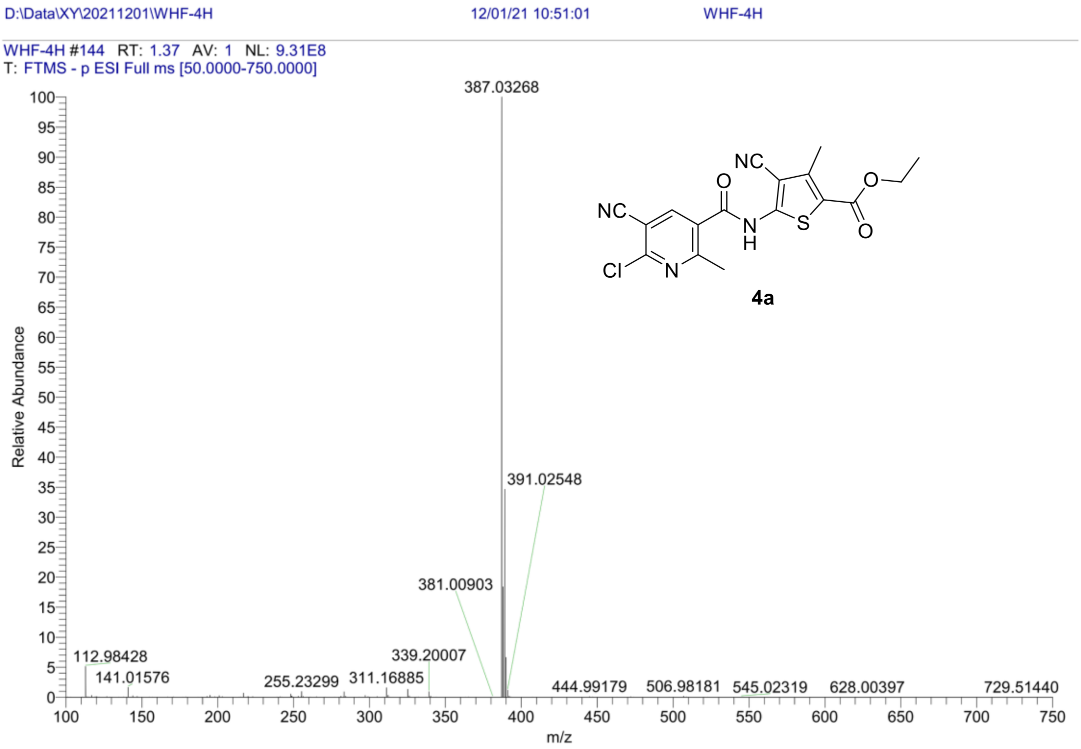

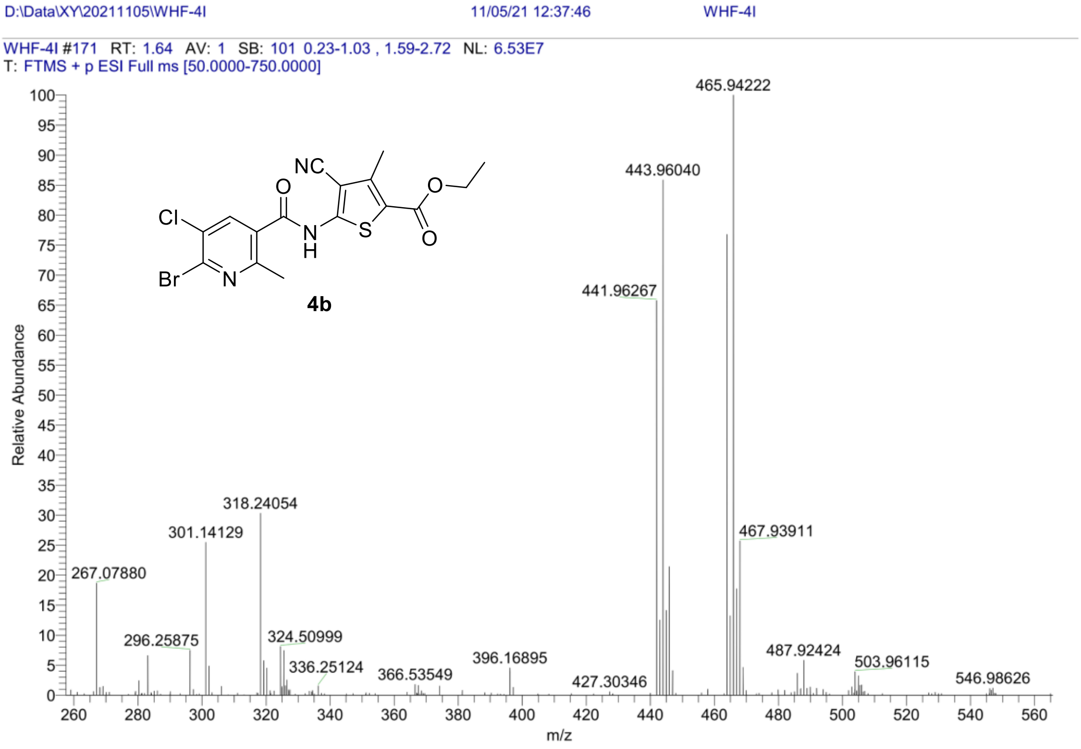

D:\Data\XY\20221104\WHF0671 11/04/22 12:44:14 WHF0671

WHF0671 #1230 RT: 11.98 AV: 1 NL: 2.21E8
T: FTMS - p ESI Full ms [100.0000-1200.0000]

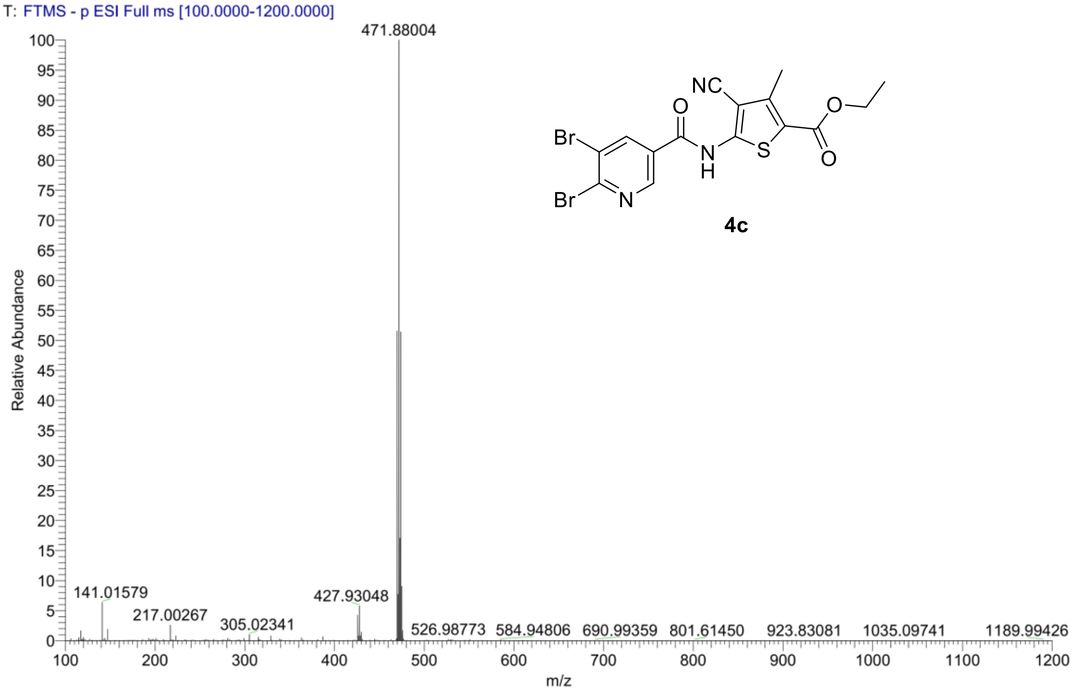

D:\Data\XY\20221104\WHF0672 11/04/22 13:08:26 WHF0672

WHF0672 #1050 RT: 10.24 AV: 1 NL: 1.24E8
T: FTMS - p ESI Full ms [100.0000-1200.0000]

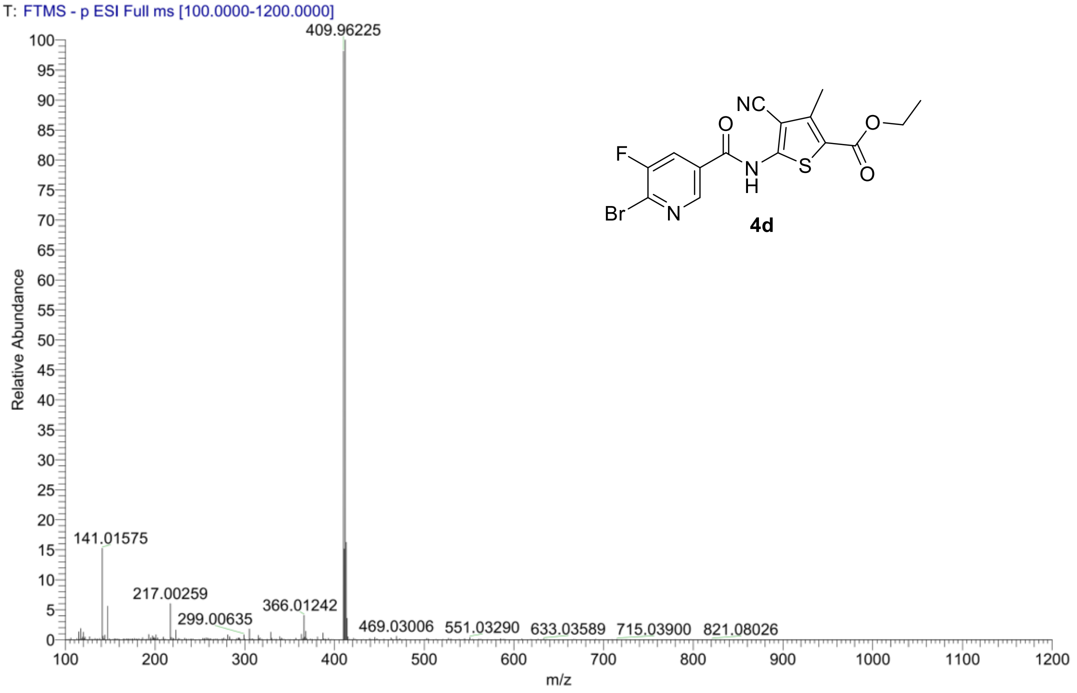

D:\Data\XY\20211201\WHF-4E 12/01/21 10:36:49 WHF-4E

WHF-4E #158 RT: 1.51 AV: 1 NL: 8.46E8
T: FTMS - p ESI Full ms [50.0000-750.0000]

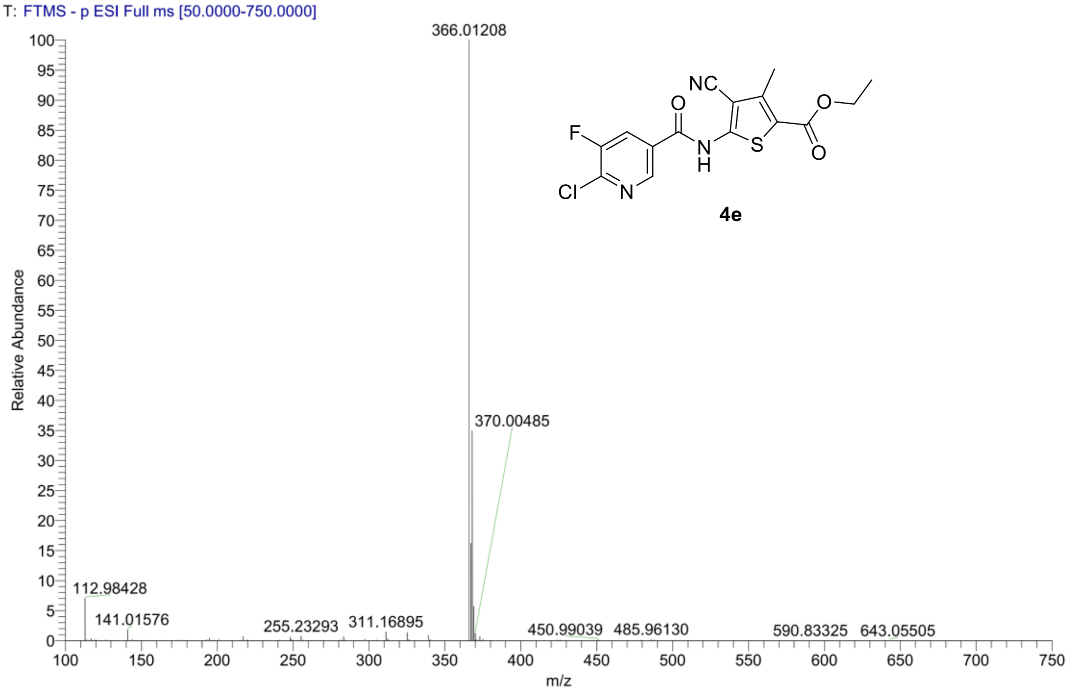

D:\Data\XY\20211105\WHF-4C-1 11/05/21 13:44:09 WHF-4C

WHF-4C-1 #173 RT: 1.66 AV: 1 SB: 102 0.23-1.03 , 1.59-2.72 NL: 5.49E7
T: FTMS + p ESI Full ms [50.0000-750.0000]

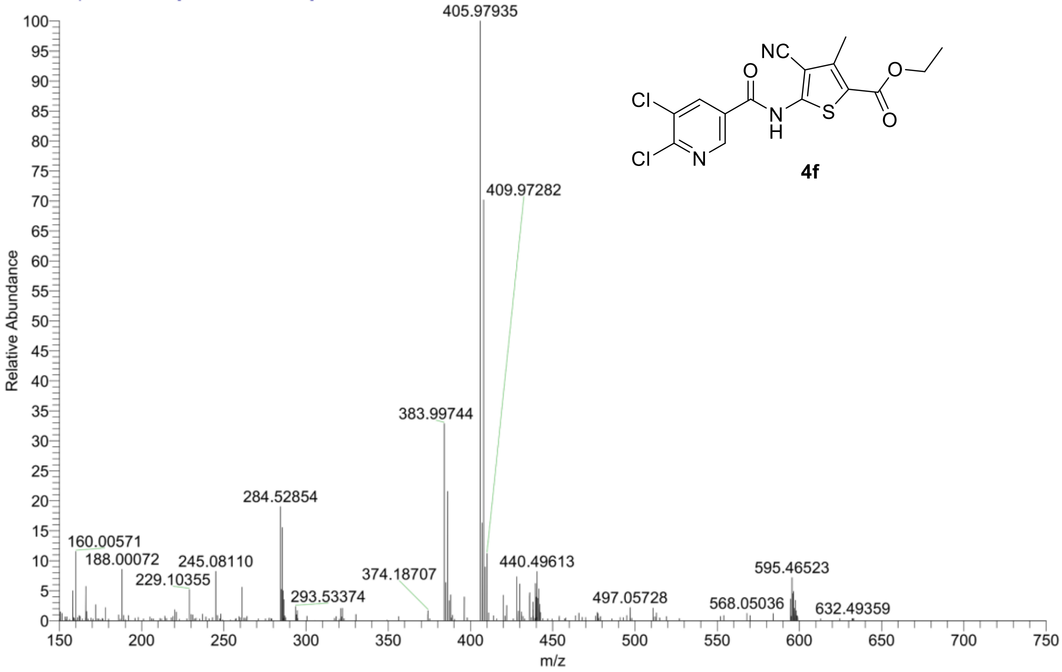

D:\Data\XY\20221104\WHF0669 11/04/22 11:57:02 WHF0669

WHF0669 #1003 RT: 9.76 AV: 1 SB: 99 0.23-1.03 , 1.59-2.72 NL: 4.91E7
T: FTMS + p ESI Full ms [100.0000-1200.0000]

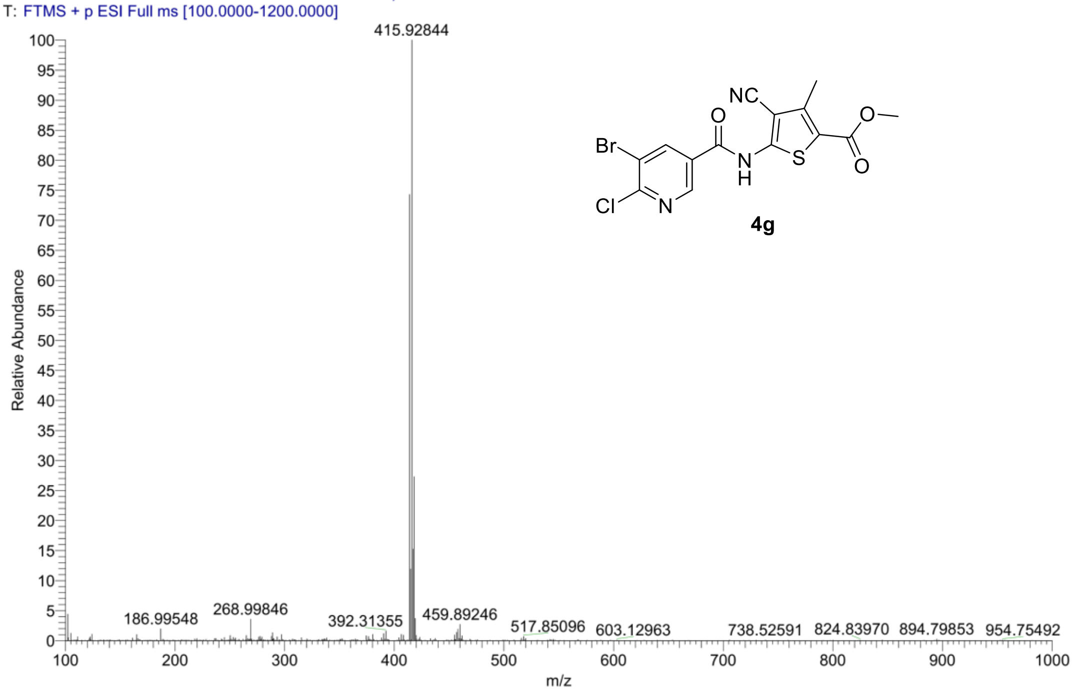

D:\Data\XY\20221104\WHF0670 11/04/22 12:21:14 WHF0670

WHF0670 #677 RT: 6.59 AV: 1 SB: 100 0.23-1.04 , 1.59-2.72 NL: 1.77E7
T: FTMS + p ESI Full ms [100.0000-1200.0000]

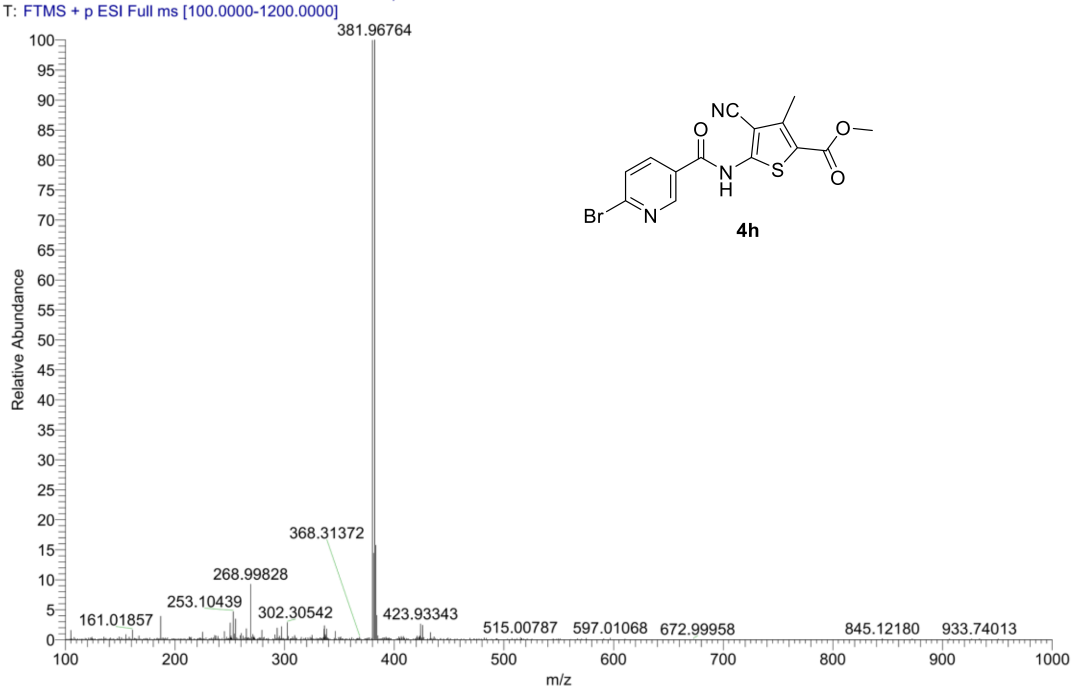

D:\Data\XY\20211201\WHF-6A 12/01/21 10:55:45 WHF-6A

WHF-6A #164 RT: 1.57 AV: 1 NL: 4.12E8
T: FTMS - p ESI Full ms [50.0000-750.0000]

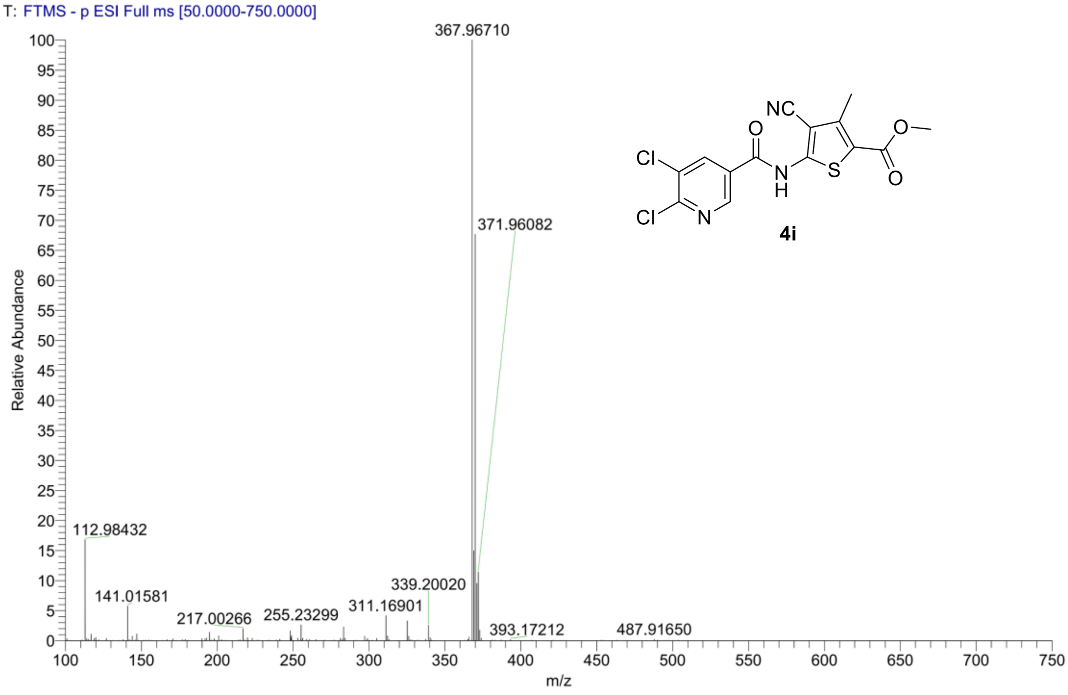

D:\Data\XY\20211201\WHF-6B 12/01/21 11:00:29 WHF-6B

WHF-6B #188 RT: 1.80 AV: 1 NL: 4.19E8
T: FTMS - p ESI Full ms [50.0000-750.0000]

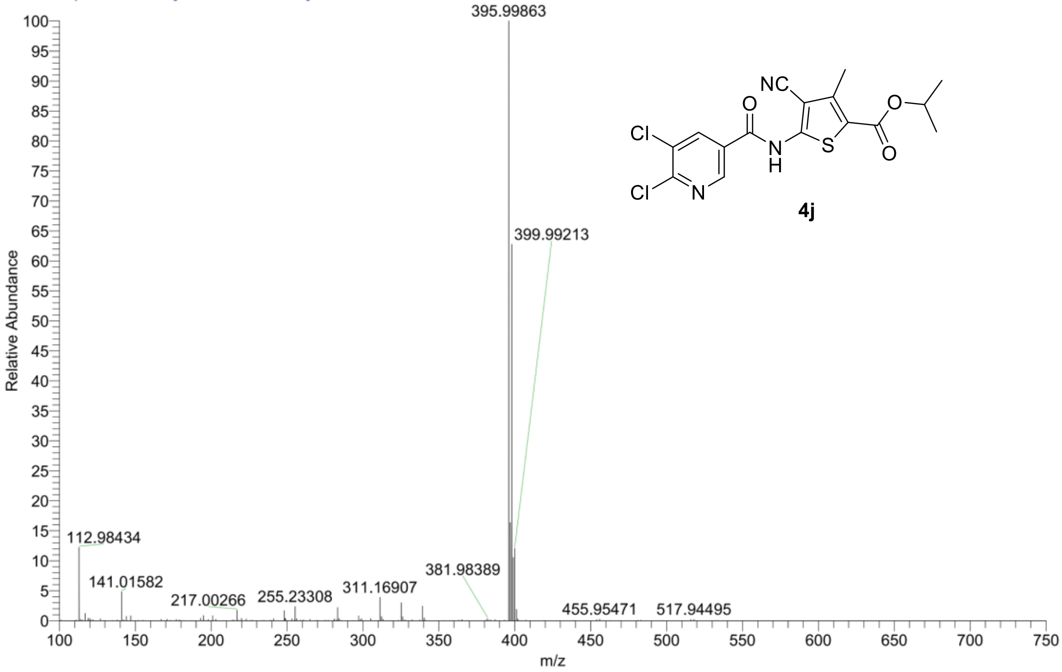

D:\Data\XY\20211201\WHF-6C 12/01/21 11:05:13 WHF-6C

WHF-6C #196 RT: 1.87 AV: 1 NL: 4.37E8
T: FTMS - p ESI Full ms [50.0000-750.0000]

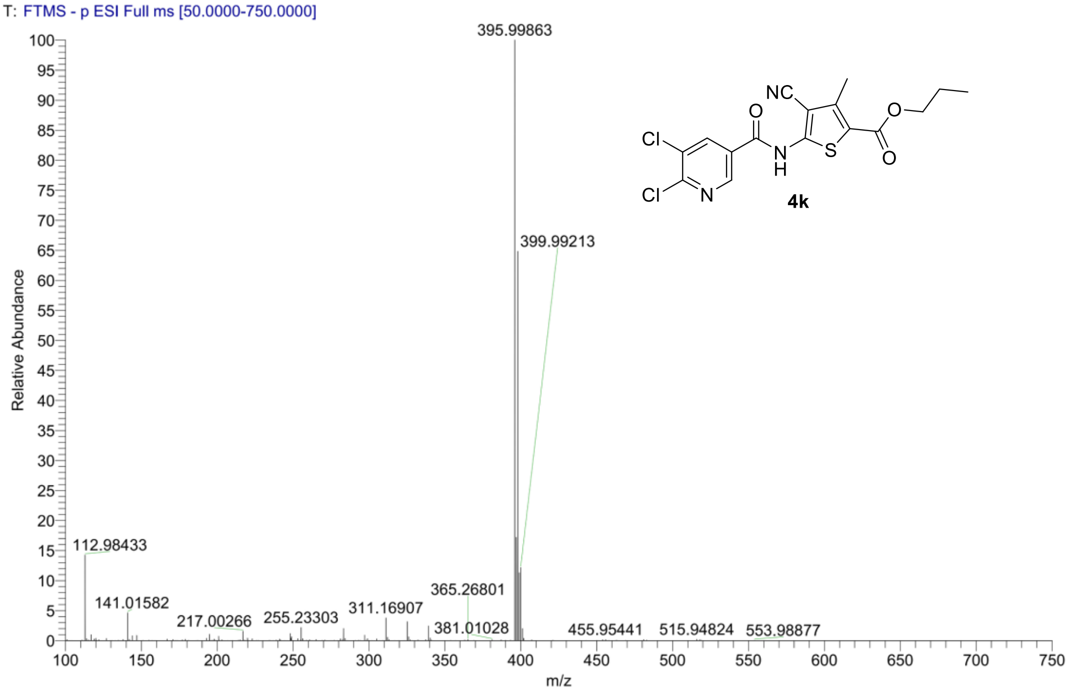

D:\Data\XY\20211201\WHF-6D 12/01/21 11:09:57 WHF-6D

WHF-6D #224 RT: 2.14 AV: 1 NL: 3.11E8
T: FTMS - p ESI Full ms [50.0000-750.0000]

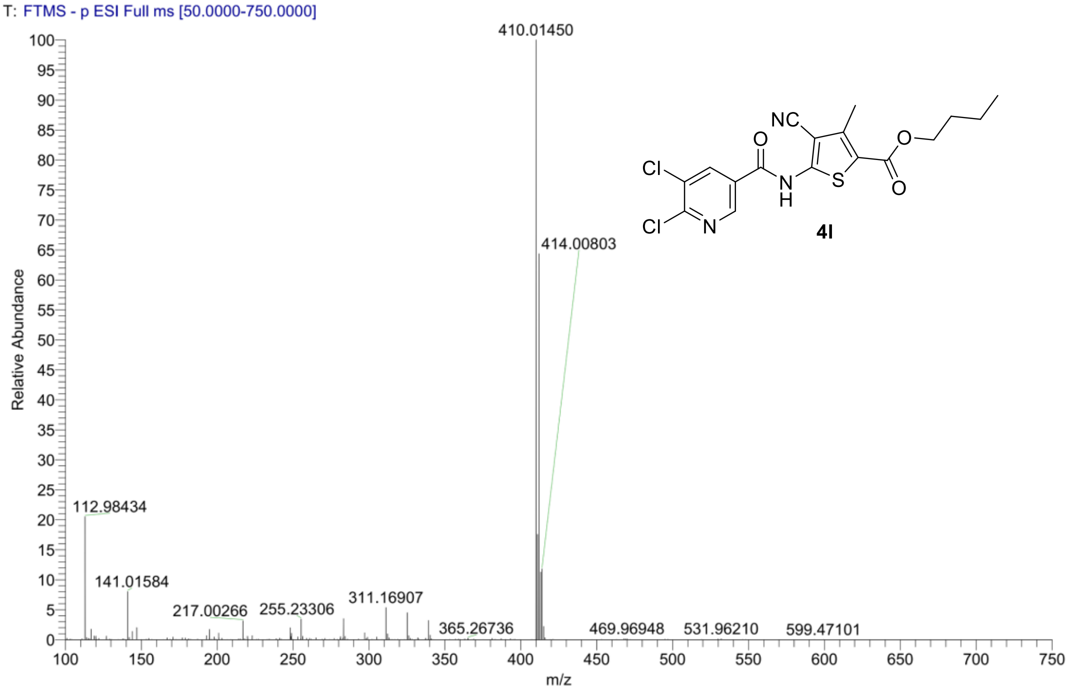

D:\Data\XY\20211201\WHF-6E 12/01/21 11:14:41 WHF-6E

WHF-6E #160 RT: 1.53 AV: 1 NL: 3.38E8
T: FTMS - p ESI Full ms [50.0000-750.0000]

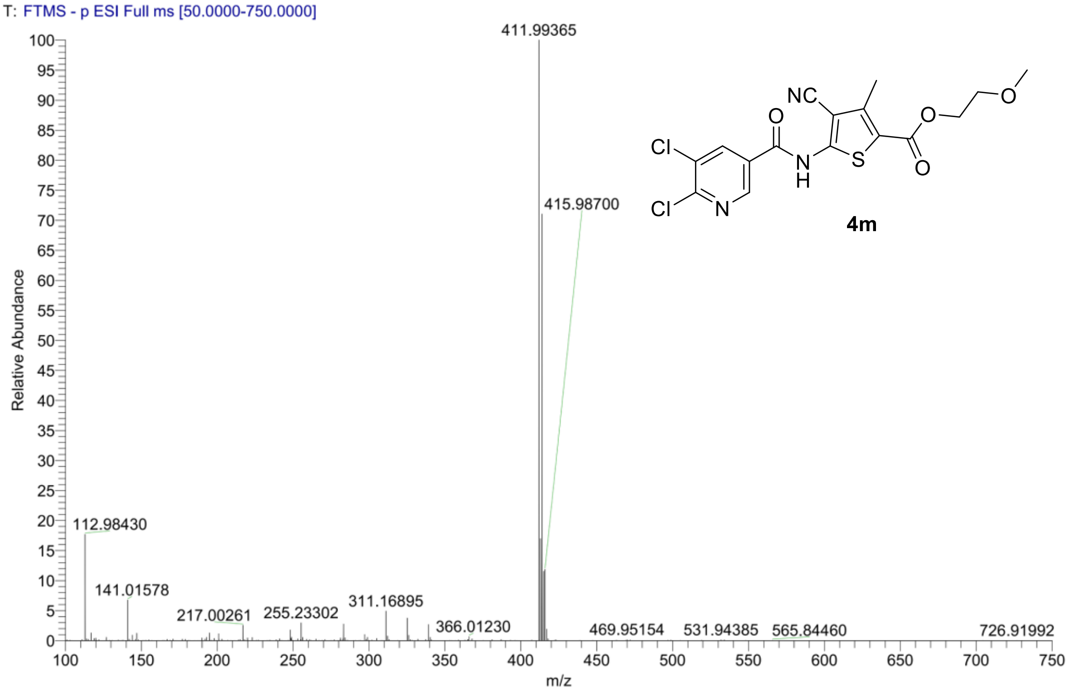

D:\Data\XY\20211201\WHF-6F 12/01/21 11:19:25 WHF-6F

WHF-6F #212 RT: 2.03 AV: 1 NL: 3.82E8
T: FTMS - p ESI Full ms [50.0000-750.0000]

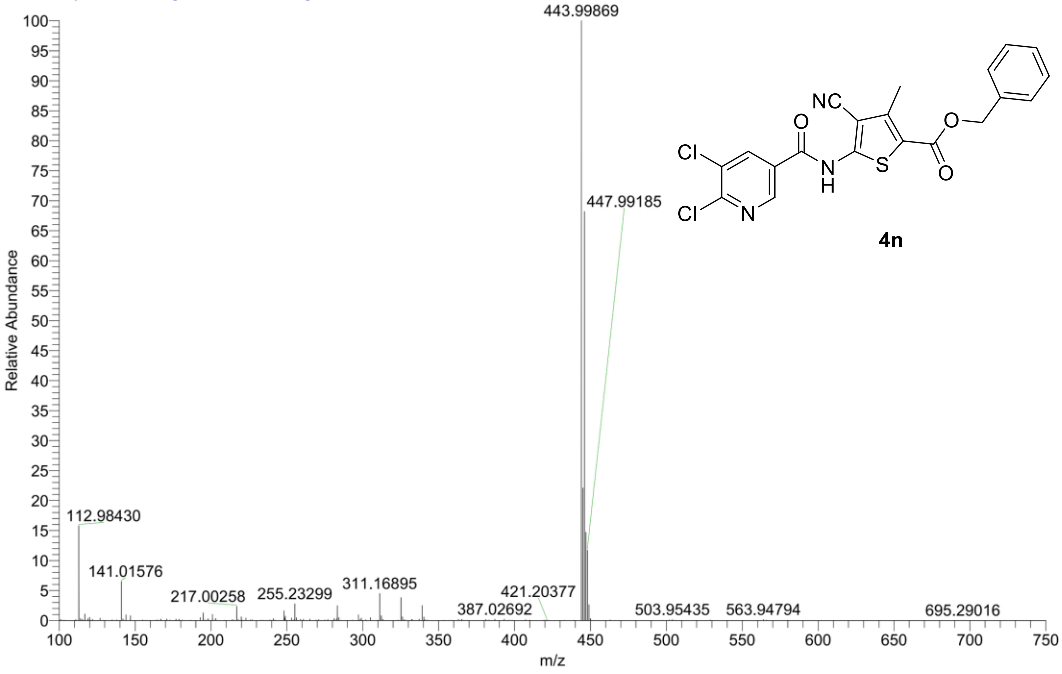

D:\Data\XY\20211201\WHF-6G 12/01/21 11:24:09 WHF-6G

WHF-6G #132 RT: 1.26 AV: 1 NL: 8.73E8
T: FTMS - p ESI Full ms [50.0000-750.0000]

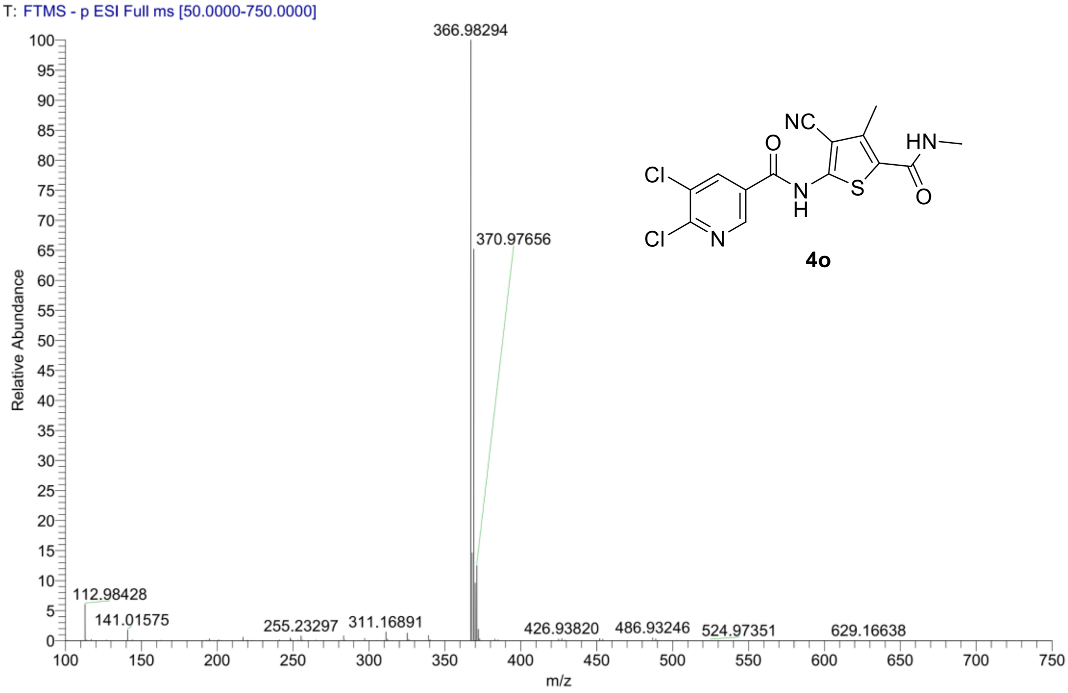

D:\Data\XY\20211201\WHF-6H 12/01/21 11:28:53 WHF-6H

WHF-6H #137 RT: 1.31 AV: 1 SB: 102 0.23-1.03 , 1.59-2.72 NL: 8.09E7
T: FTMS + p ESI Full ms [50.0000-750.0000]

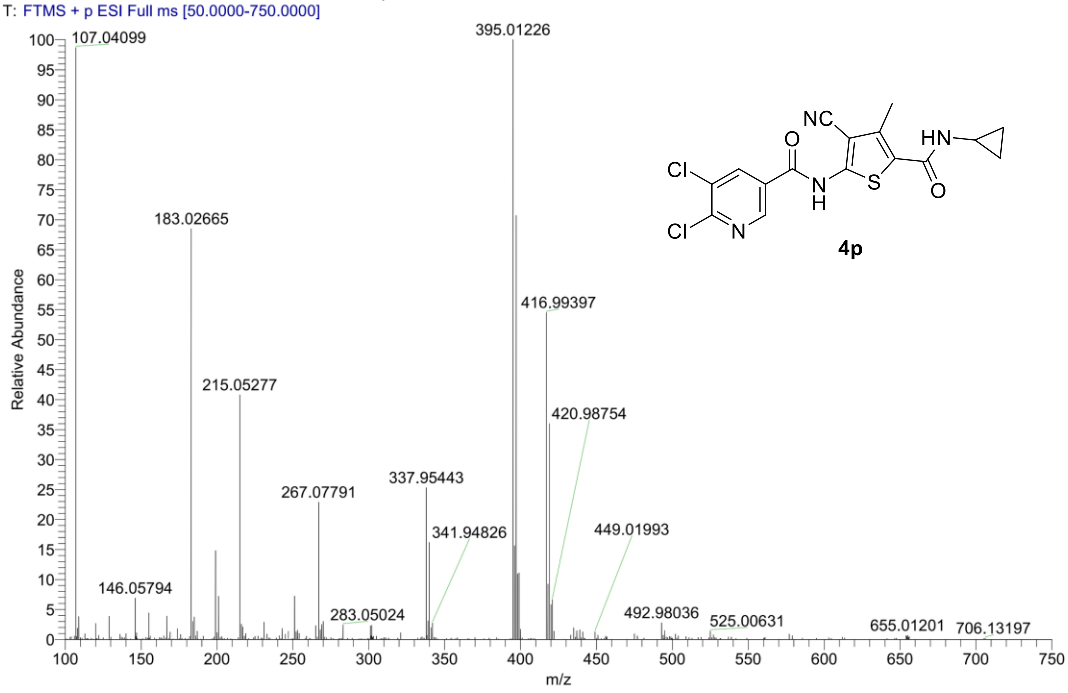

D:\Data\XY\20211201\WHF-6I 12/01/21 11:33:37 WHF-6I

WHF-6I #149 RT: 1.42 AV: 1 SB: 102 0.23-1.03 , 1.59-2.72 NL: 4.44E7
T: FTMS + p ESI Full ms [50.0000-750.0000]

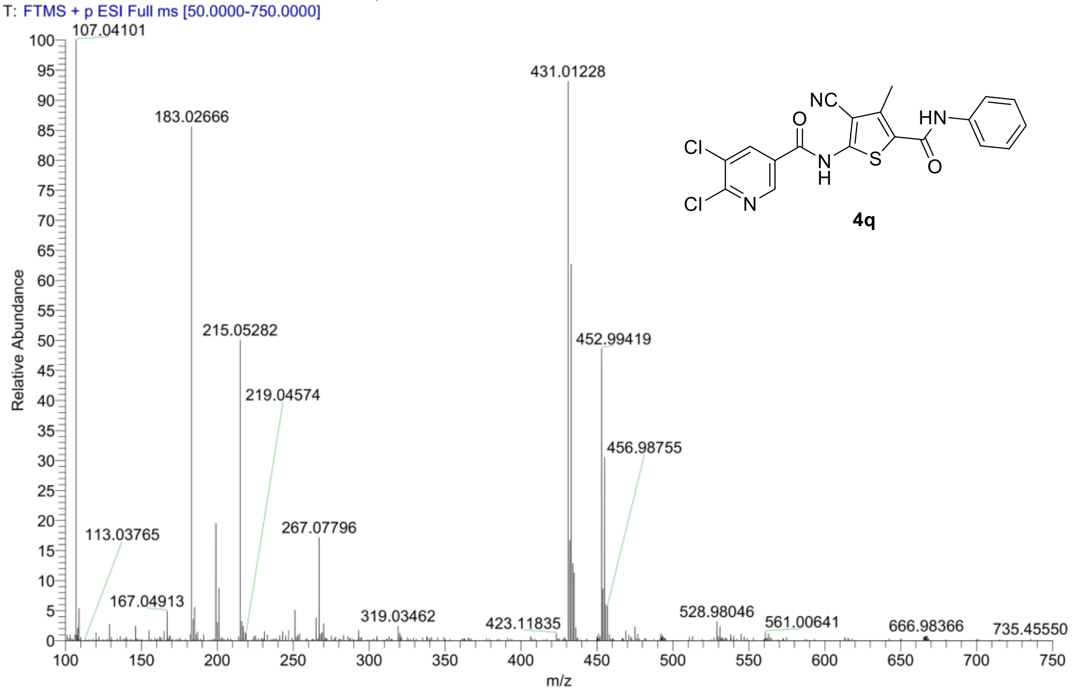

D:\Data\XY\20211201\WHF-6J 12/01/21 11:38:21 WHF-6J

WHF-6J #187 RT: 1.79 AV: 1 SB: 102 0.23-1.03 , 1.59-2.72 NL: 4.26E6
T: FTMS + p ESI Full ms [50.0000-750.0000]

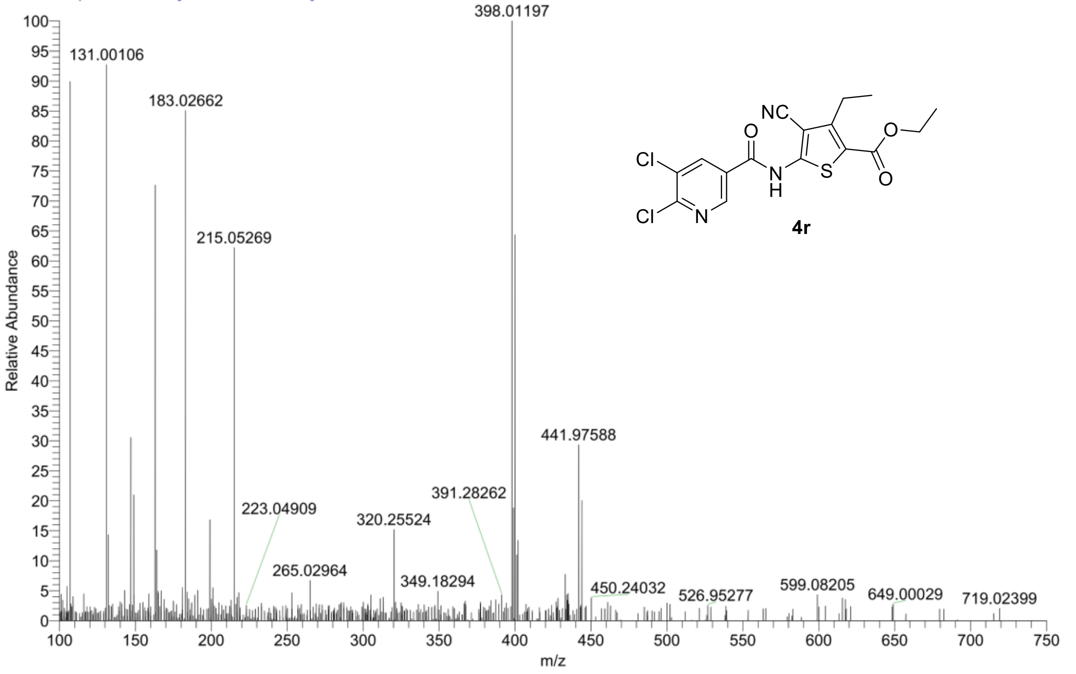

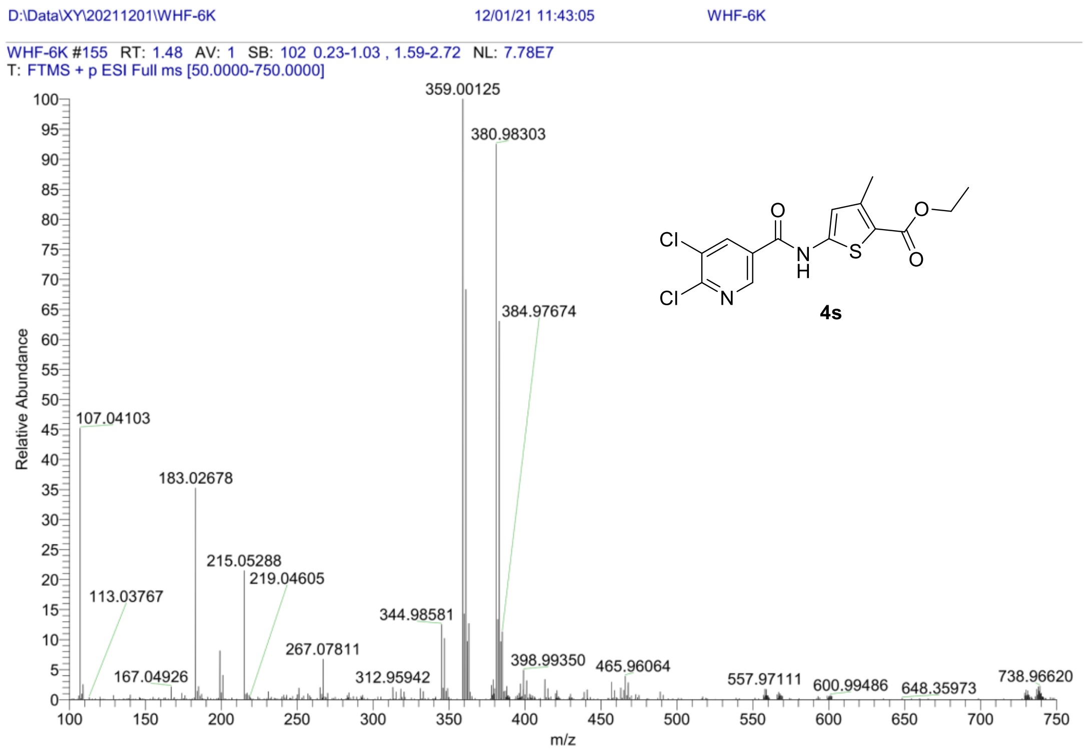

IR SPECTRAL GRAPH OF COMPOUND 4f

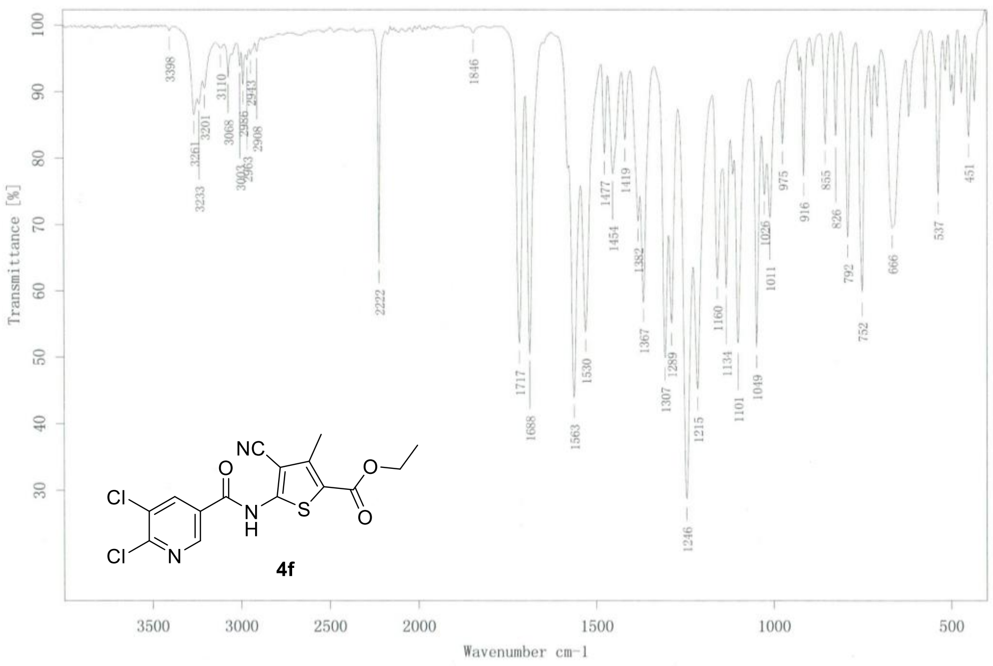

Supplement: Supplementary file 1 [file molecules-27-08700-s001.zip › molecules-2076303-supplementary.pdf]
